# Conformal Field Theory at the Lattice Level: Discrete Complex Analysis and Virasoro Structure

**DOI:** 10.1007/s00220-022-04475-x

**Published:** 2022-09-06

**Authors:** Clément Hongler, Kalle Kytölä, Fredrik Viklund

**Affiliations:** 1 Chair of Statistical Field Theory, Institute of Mathematics, EPFL Station 8, CH 1015 Lausanne, Switzerland; 2 Department of Mathematics and Systems Analysis, Aalto University, P.O. Box 11100, RI 00076 Aalto, Finland; 3 Department of Mathematics, KTH Royal Institute of Technology, 100 44 Stockholm, Sweden

## Abstract

Critical statistical mechanics and Conformal Field Theory (CFT) are conjecturally connected since the seminal work of Beliavin et al. (Nucl Phys B 241(2):333–380, 1984). Both exhibit exactly solvable structures in two dimensions. A long-standing question (Itoyama and Thacker in Phys Rev Lett 58:1395–1398, 1987) concerns whether there is a direct link between these structures, that is, whether the Virasoro algebra representations of CFT, the distinctive feature of CFT in two dimensions, can be found within lattice models of statistical mechanics. We give a positive answer to this question for the discrete Gaussian free field and for the Ising model, by connecting the structures of discrete complex analysis in the lattice models with the Virasoro symmetry that is expected to describe their scaling limits. This allows for a tight connection of a number of objects from the lattice model world and the field theory one. In particular, our results link the CFT local fields with lattice local fields introduced in Gheissari et al. (Commun Math Phys 367(3):771–833, 2019) and the probabilistic formulation of the lattice model with the continuum correlation functions. Our construction is a decisive step towards establishing the conjectured correspondence between the correlation functions of the CFT fields and those of the lattice local fields. In particular, together with the upcoming (Chelkak et al. in preparation), our construction will complete the picture initiated in Hongler and Smirnov (Acta Math 211:191–225, 2013), Hongler (Conformal invariance of ising model correlations, 2012) and Chelkak et al. (Annals Math 181(3):1087–1138, 2015), where a number of conjectures relating specific Ising lattice fields and CFT correlations were proven.

## Introduction

### Statistical mechanics and conformal field theory

Physical arguments suggest that 2D lattice models at continuous phase transitions have conformally invariant scaling limits that can be described by Conformal Field Theories (CFTs). The 2D CFTs are exactly solvable in the sense that they can be studied in terms of representations of the Virasoro algebra. This has led to exact formulae for the (conjectural) scaling limits of correlations, partition functions, and critical exponents of such lattice models. See, e.g., [BPZ84a, BPZ84b, DMS97, Mus09] and the references in the latter.

Despite the success of the application of CFT to lattice models, it usually constitutes a non-rigorous approach to statistical mechanics. Indeed, one needs to assume that the fields of the models have conformally invariant scaling limits and that they can be described within the framework of certain quantum field theories.

In the special case of the (discrete) Gaussian free field, the CFT approach can be carried out rigorously and Virasoro representations can be found in the continuum in terms of insertions [Gaw99, KaMa11]. For the Ising model, significant progress towards connecting its correlations with the correlation of the relevant CFTs has been made recently [CHI15, Hon10, HoSm13].

Schramm’s SLEs provide a different route towards a rigorous understanding of conformally invariant scaling limits [Sch00, Sch07]. These random curves describe the scaling limits of cluster interfaces in the lattice models. Moreover, SLE processes are amenable to calculations and SLE techniques have been successfully applied to produce interpretations and rigorous proofs of a number of the conjectures of CFT, see, e.g., the references in [KaMa11]. Studies of more direct and systematic connections between SLE and CFT have been carried out, leading to beautiful results. See, e.g., [BaBe03, DRV06, FrWe03, KaMa11, Kyt07, Dub15b, Dub15c, FlKl15].

Still, the connection between lattice models and CFT remains far from well-understood and, it seems fair to say, rather mysterious from a mathematical perspective. Thus it appears of significant interest to advance the mathematical understanding of CFT applied to lattice models. There are many fundamental difficulties, including in particular the correspondence between discrete and continuous quantities, the proofs of conformal invariance, the locality of the limits, and the positivity of the underlying representations, to name a few.

### Exact solvability

In two dimensions, a number of lattice models (see Sect. 1.3 below) are considered exactly solvable in a different sense than CFTs. Typically, exact solvability in this context means that certain lattice-level relations such as the Yang-Baxter equations are present and this often yields exact formulae for a number of interesting quantities of the models. In fact, this is how most of the exact results about lattice models were derived in the 20th century. See, e.g., [Bax89] and references therein.

Recently, discrete solvability has been formulated in terms of *discrete holomorphicity*. This has enabled the use of discrete complex analysis techniques leading to rigorous proofs of conformal invariance for a number of exactly solvable lattice models and to exact formulae for their limiting correlations, matching the predictions of the relevant CFTs. See, e.g., [Ken00, Ken01, Smi06, Smi10a, CHI15].

Once the lattice solvability has been used to establish conformal invariance, the latter should in principle help reveal the algebraic structures of CFT. Thus it is natural to expect lattice and CFT solvability to be (indirectly) connected via continuous conformally invariant objects such as SLE or the GFF or by the identification of lattice precursors of key CFT objects, see, e.g., [BeHo17, Dub15a, Dub11b, Dub15c, CGS16] for recent progress in this direction.

This leads to the following natural question formulated already in the late 1980s [ItTh87]:

- Is there a *direct* connection between exact solvability of lattice models and of conformal field theories?

In this paper we resolve this longstanding problem in the positive for the discrete Gaussian free field and the critical Ising model: the central algebraic structures of the CFTs describing their scaling limits are in fact already present at the lattice level.

This question has been investigated in the physics literature in the case of the 8-vertex model and the Ising model, see [ItTh87, KoSa94] and the references in the latter, as well as the recent development [GJRSV13]. However, our results are the first where the relevant lattice and continuous structures are directly and exactly connected (without deformation). Moreover, the action of the operators takes place on a space of lattice ancestors of the local fields, thus giving a transparent, probabilistic interpretation of the situation, and is formulated in terms of discrete complex analysis, enabling control of the limits.

### Lattice models

A lattice model is informally a probabilistic or quantum model which “lives” on a graph or lattice such as ${\mathbb {Z}}^2$: there are (random or quantum) degrees of freedom associated with each vertex, edge, or face of the lattice. Of particular interest are the large-scale features of such models, particularly when they result in randomness at large scale suggesting the existence of scaling limits, i.e., macroscopic random objects which describe the models as one “looks at them from far away.”

This paper focuses on two classical probabilistic lattice models: the discrete Gaussian free field (defined precisely in Sect. 3.2) and the Ising model (defined precisely in Sect. 3.3), both with one degree of freedom associated with each vertex. Their scaling limits are the most fundamental examples of CFTs.

#### Discrete Gaussian free field

The discrete Gaussian free field (dGFF) on a graph *G* is a random Gaussian vector $\left( {\varphi }_{x}\right)$ with entries indexed by the vertices *x* of *G* with density proportional to $\exp \big ( -\mathrm {const.}\sum _{x\sim y} \left( {\varphi }_{x}-{\varphi }_{y}\right) ^{2} \big )$ where the sum is over all pairs of adjacent vertices; see Sect. 3.2 for the precise conventions that we will use.

By taking an appropriate scaling limit of the dGFF, one recovers the Gaussian free field (also known as the massless free boson field) which plays a central rôle in Quantum Field Theory. The continuum GFF is well-understood mathematically, see, e.g., [Gaw99, GJ87, KaMa11, She07].

#### Ising model

The Ising model is perhaps the most well-studied model of equilibrium statistical mechanics. It consists of random $\pm 1$-valued spins $\left( {\sigma }_{x}\right)$ living on the vertices *x* of a graph *G*. The probability of a spin configuration is proportional to $\exp \big ( -{{\beta }}\, {\mathcal {H}}[{\sigma }] \big )$, where the energy ${\mathcal {H}}[{\sigma }] = - \sum _{x\sim y} {\sigma }_{x} {\sigma }_{y}$ is obtained by summing over all pairs of adjacent vertices and ${{\beta }}>0$ is called the inverse temperature. The large-scale behavior of the model depends strongly on ${{\beta }}$: if we consider the Ising model on a large subset of ${\mathbb {Z}}^2$, a long range alignment will occur if and only if ${{\beta }}> {{\beta }}_{\mathrm {cr.}}:= \frac{1}{2}\ln \left( \sqrt{2}+1\right)$, while the system will look disordered at large scale for ${{\beta }}< {{\beta }}_{\mathrm {cr.}}$. The *critical regime* ${{\beta }}={{\beta }}_{\mathrm {cr.}}$ has been the object of much attention in the last decades: in particular, one of the motivations for the study of CFT is to describe the scaling limit of this model.

### Conformal field theory and virasoro algebra

In this subsection we briefly outline a number key ideas of CFT, in particular the Virasoro algebra.

#### Statistical field theory

A (physical) theory aimed at describing a random system with infinitely many degrees of freedom is often called a *statistical field theory*. Of particular interest are the (conjectural) scaling limits of lattice models, i.e., the limits of lattice models living on ${\delta }$-meshed discretizations ${{\Omega }}_{{\delta }}$ of continuous domains ${\Omega }\subset {\mathbb {R}}^{n}$, as ${\delta }\rightarrow 0$. It is expected, but unproven except in a few cases, that most lattice models with an infinite correlation length converge to non-trivial scaling limits that can be described by statistical field theories.

A statistical field theory on ${\Omega }\subset {\mathbb {R}}^{n}$ is usually *thought of *as a random process ${\phi }$ with a measure ${\mathsf {P}}\left\{ {\phi }\right\} \propto \exp \big ( -{{\mathcal {S}}}\left[ {\phi }\right] \big )$, where ${{\mathcal {S}}}$ is a functional called the *action*. The main objects of interest are the correlations of *local fields* ${{\mathcal {O}}}_{j}$, roughly speaking functions of ${\phi }$ in an infinitesimal neighborhood of their point of insertion, for example derivatives of ${\phi }$. The correlations $\big \langle \, { {{\mathcal {O}}}_{1}(z_{1}) \cdots {{\mathcal {O}}}_{n}(z_{n}) } \, \big \rangle _{{\Omega }}$ are thought of as functional integrals

1.1 $$\begin{aligned} \frac{\int {{\mathcal {O}}}_{1}(z_{1}) \left[ {\phi }\right] \cdots {{\mathcal {O}}}_{n}(z_{n}) \left[ {\phi }\right] e^{-{{\mathcal {S}}}\left[ {\phi }\right] } \; {{\mathcal {D}} {\phi }}}{ \int e^{-{{\mathcal {S}}}\left[ {\phi }\right] } \; {{\mathcal {D}} {\phi }}}, \end{aligned}$$

over all the possible realizations of the field ${\phi }$ defined on ${\Omega }$. This is natural, e.g., by analogy with the definition of lattice models such as the dGFF and the Ising model.

Unfortunately, all of the above is difficult to make precise. Instead, a common approach is to *define* local fields as being *objects one can take (abstract) correlations of*. One hence considers a space ${\mathcal {F}}$ of local fields, equipped with multilinear operations

$$\begin{aligned} {\mathcal {F}}^{n} \ni \left( {{\mathcal {O}}}_{1}, \ldots , {{\mathcal {O}}}_{n} \right) \mapsto \big \langle \, { {{\mathcal {O}}}_{1}(z_{1}) \cdots {{\mathcal {O}}}_{n}(z_{n}) } \, \big \rangle _{{\Omega }} \in {\mathbb {C}}, \end{aligned}$$

defined for distinct points $z_{1}, \ldots , z_{n} \in {\Omega }$. A number of axioms are then added corresponding to what the abstract correlations are expected to satisfy (positivity, etc.), were they to arise from functional integrals as in (1.1). This is one of the standard approaches to CFT [Seg88, Seg04]. One of the eventual outcomes of this paper is an alternative route to understanding (at least) certain field theories, which restores part of this probabilistic picture, and brings the original spirit of functional integrals much closer.

#### Conformal field theory

A Euclidean *Conformal Field Theory *on ${\Omega }\subset {\mathbb {C}}$ is informally a statistical field theory with conformal symmetry. Conformal symmetry is thought of as a symmetry of the action functional ${{\mathcal {S}}}$. Conformal symmetry can then be *defined* by postulating the existence of a special local field *T*, called the *holomorphic Stress-Energy Tensor*.^1^ Its correlations

$$\begin{aligned} {\Omega }\setminus \left\{ z_{j} \right\} \ni z \mapsto \big \langle \, { T(z) \prod _{j} {{\mathcal {O}}}_{j}(z_{j}) } \, \big \rangle \end{aligned}$$

are holomorphic and have prescribed poles as $z \rightarrow z_{j}$.

The poles of *T*(*z*)*T*(*w*) as $z \rightarrow w$ are in particular given by the so-called *Conformal Ward Identity*^2^

1.2 $$\begin{aligned} T(z) T(w) =\frac{c/2}{\left( z-w\right) ^{4}}+\frac{2T(w) }{ \left( z-w\right) ^{2}}+\frac{\partial T(w) }{z-w}+\mathrm {reg}, \end{aligned}$$

where the number $c\in {\mathbb {R}}$ is an important parameter, characteristic of the CFT in question, and is called its *central charge.*

#### Virasoro algebra

A key insight of 2D field theory is that the modes of the stress-energy tensor *T* can act as operators on other local fields: for each $n\in {\mathbb {Z}}$ and ${{\mathcal {O}}}\in {\mathcal {F}}$, one defines a field^3^${\mathsf {L}}_{n} {{\mathcal {O}}}\in {\mathcal {F}}$ by

1.3 $$\begin{aligned} {\mathsf {L}}_{n}{{\mathcal {O}}}(z) := \lim _{\epsilon \rightarrow 0_{+}} \; \oint _{|\zeta -z|=\epsilon } T(\zeta ) {{\mathcal {O}}}(z) \left( \zeta -z\right) ^{n+1} \mathrm {d}\zeta . \end{aligned}$$

From (1.2), the operators $\left( {\mathsf {L}}_{n}\right) _{n\in {\mathbb {Z}}}$ can then be shown to form a representation of the Virasoro algebra of central charge *c*, i.e., their commutation relations are

1.4 $$\begin{aligned} \left[ {\mathsf {L}}_{n} , {\mathsf {L}}_{m} \right] = \left( n-m\right) {\mathsf {L}}_{n+m} + \frac{c}{12}\left( n^{3}-n\right) \delta _{n,-m} . \end{aligned}$$

The Virasoro algebra is the cornerstone for the algebraic exact solution of CFT: the ${\mathsf {L}}_{n}$ operators can be studied in terms of Virasoro representation theory; further down this road, by their definition in terms of *T*, the ${\mathsf {L}}_{n}$’s yield precise geometric information about the correlations which, e.g., can be cast as linear partial differential equations for correlation functions.

#### Vertex operator algebras and Sugawara construction

The key feature that enables the construction of linear operators on the space of local fields is the holomorphicity of the stress tensor (and it is the geometric importance of the latter which then allows one to derive results about correlations). Large classes of CFTs possess a number of holomorphic fields besides the stress energy tensor and its descendants (the Virasoro subrepresentation generated by *T*). These CFTs, whose algebraic axiomatization is *Vertex Operator Algebras* (VOAs), are the object of many beautiful insights of mathematical physics, representation theory, and string theory (see [Bor86, FLM88, Kac82]).

The CFTs of the GFF and the Ising model both possess a VOA structure, based on the current field (for the GFF) and the fermion field (for the Ising CFT) respectively. The modes of these holomorphic fields and their commutation relations can be studied in a fashion that is similar to the way the modes of the stress-tensor are studied. Furthermore, the stress tensor of these theories can be constructed in terms of the currents and fermions, and as result, the modes of the stress tensor can be constructed in terms of the modes of the current and fermion, through what is known as the Sugawara-Sommerfield construction [Sug68, Som68]. The main result of this paper relies crucially on this construction.

### Strategy

The approach suggested in [GHP19] and in the present paper is quite different from the usual axiomatic approach to CFT: we look at lattice models as precursors of the field theories. The (often ill-defined) process ${\phi }:{\Omega }\rightarrow {\mathbb {R}}$ is replaced by a random function ${\phi }_{{\delta }} :{{\Omega }}_{{\delta }}\rightarrow {\mathbb {R}}$, where ${{\Omega }}_{{\delta }}$ is a discretization of ${\Omega }$. The functional integral formalism is then perfectly well defined. Indeed, the lattice analogues of local fields ${{\mathcal {O}}}_{{\delta }}$ are defined as functions of values of the process ${\phi }_{{\delta }}$ on finitely many neighbors of the insertions, and correlation functions are just expected values.

The obvious drawback is that lattice models have no conformal symmetry: indeed, such models are not invariant under scaling or (most) rotations, let alone more general conformal mappings. Nevertheless, some lattice models, such as the dGFF and the Ising model, possess a number of discrete holomorphic fields, i.e., fields whose correlations satisfy lattice analogues of the Cauchy–Riemann equations. If we could find a suitable lattice ancestor of *T*—a discrete holomorphic lattice local field satisfying a discrete version of the Conformal Ward Identity (1.2)—we might hope to be able to realize the Virasoro algebra at the lattice level.

However, the discrete holomorphic fields of the dGFF and the Ising Model are not lattice ancestors of the stress tensor, but of (in some sense) more primitive objects; the current and the fermion, respectively. Since discrete holomorphicity is a rather fragile property (for instance, it is not even preserved by squaring), it is not obvious how to construct more sophisticated discrete holomorphic fields from them.

The approach of this paper relies on revealing, at the lattice level, the extended Vertex Operator Algebra structure that both models carry, which involves the current and fermion modes. The Virasoro generators can then be constructed on relevant lattice local field spaces as bilinear products of these modes, through the Sugawara construction. Remarkably, the whole construction can be carried out at the lattice level, yielding the same exact commutation relations as in the scaling limit, while acting on probabilistically transparent objects.

#### Remark 1.1

In [CGS16], a discretized counterpart of the stress-energy tensor *T* has been proposed on the hexagonal lattice, and its correlation in the scaling limit have been identified. While the identification of this lattice field stems from considerations similar to ours (finding lattice precursors of CFT objects), it does not lead to the Virasoro algebra structure at the lattice level: the field identified there is indeed not discrete holomorphic, there is no obvious way to define consistent lattice analogues of its modes acting on the space of fields, and no reason to expect exact Virasoro relations to arise from it. Nevertheless, it remains an interesting question to explore the connections between this field and our construction.

### Main result and applications

As explained in Sect. 1.4, the Virasoro algebra in CFT acts on a space of local fields. In this paper, we consider the lattice analogue of local fields proposed in [GHP19], and we define relevant operators on that space, allowing for a construction of the full Virasoro symmetry on it.

#### Lattice local fields

A lattice local field is a natural generalization of fields of the form $x\mapsto {\varphi }_{x}$, $x\mapsto {\varphi }_{x}^{2}$, $x\mapsto {\sigma }_{x}{\sigma }_{x+{\delta }}$, $x\mapsto {\sigma }_{x+{\delta }}-{\sigma }_{x}$, etc., namely a translation-invariant functional that depends on a finite number of variables applied to the dGFF and Ising basic fields ${\varphi }_{x}$ and ${\sigma }_{x}$. See Definition 3.3 in Sect. 3.1 for a more precise definition. We call a lattice local field *null* if its correlations against other lattice local fields (taken at large enough distance) are zero. Note that null fields are not necessarily zero in a given realization, for example the discrete Laplacian of the dGFF has vanishing correlations. The connection between lattice local fields and CFT local fields (which can serve as a probabilistic definition of the latter, since none has been given) has been conjectured in, e.g., [GHP19].

#### Main result

Let ${\mathfrak {F}}_{{\mathcal {G}}}$ denote the space of lattice local fields of the dGFF, modulo its null fields, and let ${\mathfrak {F}}_{{\mathcal {I}}}$ denote the space of lattice local fields of the critical Ising model, modulo its null fields.

Our main theorem can then be informally phrased as follows.

##### Theorem

The Sugawara constructions of the Virasoro modes of the dGFF and of the critical Ising model can be naturally and exactly realized at the lattice level on the space ${\mathfrak {F}}_{{\mathcal {G}}}$ and ${\mathfrak {F}}_{{\mathcal {I}}}$ respectively, by considering discrete complex Laurent modes of the lattice current and lattice fermion, respectively.

The precise form of the theorem, as well as its proof, is given in Sect. 4, in the form of Theorem 4.10 (dGFF case) and Theorem 4.24 (Ising case).

By the link between the discrete and continuous structures that it establishes, our main theorem yields an improved understanding of both:

- On the one hand, the construction demonstrates how the lattice solvability can be directly expressed in terms of the algebraic structures of CFT. This gives a convincing answer to the classical question of their connection [ItTh87] and opens the possibility to understand more structures related to Vertex Operator Algebras in a similar manner.
- On the other hand, by giving a natural lattice construction of the objects of CFT, it gives the possibility of understanding CFTs in probabilistic terms. As mentioned in Sect. 1.5, this can be achieved by finding the proper (manifestly probabilistic) discrete analogues of the field-theoretic concepts, and (later) establishing their convergence in the scaling limit.

These two directions, and some applications, are detailed in the next two paragraphs.

#### Application: algebraic structures

The study of the CFTs in terms of their Vertex Operator Algebra structures is a major branch of CFT [FLM88], which has ramifications in string theory, condensed matter physics, and representation theory. Most of the mathematical works on such theories rely on a formal and abstract axiomatic construction of field theories, and as such often appear daunting. It now appears that a significant part of the relevant structures can be constructed very concretely at the lattice level, using the techniques introduced in this paper, thus significantly facilitating the understanding of these structures. While these developments will be studied in a subsequent paper, we briefly outline two particular (related) constructions of CFT, which appear to be amenable to lattice constructions such as the ones proposed in this paper: the Coulomb gas formalism and the Affine Kac-Moody algebra CFTs. The main idea that emerges is: *many of the important algebraic structures of CFT can emerge from lattice solvability phrased as discrete holomorphicity.*

The so-called Dotsenko-Fateev Coulomb gas construction is a fundamental idea of CFT [DoFa84, Fel89, DMS97], which informally relies on considering complex exponentials of the GFF. Within this framework it is possible to vary the central charge of the theory, and thus to construct explicitly a large number of CFTs modeled on a Gaussian structure. The constructions of the Coulomb gas theory can be phrased in terms of deformations of the Sugawara construction (see, e.g., [Fel89, KaMa11, Mic89]). Using a modified version of our dGFF construction (corresponding to other central charges) it is possible to reveal them exactly at the lattice level. This will allow for constructions of lattice precursors of a number of objects of central importance in CFT, which moreover appear connected to other lattice models, such as dimers.

A number of important examples of CFTs are those endowed with extensions of the Virasoro algebra symmetry called Affine Kac-Moody (AKM) algebras. These have found applications in condensed matter physics and in string theory, and are at the heart of coset field theories, which are among the most general classes of CFTs (in particular, coset CFTs include all the minimal models). The most basic example of AKM CFT is the Gaussian free field, which is endowed with its Heisenberg current algebra, which is precisely the structure that we reveal at the discrete level. Thanks to the so-called Wakimoto construction, AKM CFTs can be constructed by taking several independent copies of the GFF, through a scheme similar to that of the Coulomb Gas construction. Further down this road, a number of constructions involving several copies of the Ising fermions, in particular the theory of the framed Vertex Operator Algebras, have recently yielded important results in representation theory of finite groups [Miy04], and it appears that realizing them on the lattice level would give new insights and allow for a number of simplifications.

#### Application: probabilistic field theories

The other promising line of research emerging from this paper is the possibility to give clear and precise probabilistic meaning to CFT objects, thus enabling one to restore the “original” point of view of such theories in terms of functional integrals. While discretizing quantum field theories as a way to regularize them is an old idea, promoted in particular by Kenneth Wilson [Car13, WiKo74] and now viewed as the best way to mathematically understand them, the following is new: the possibility to identify transparentely the operator content of CFTs such as the one describing the Ising model, at the lattice level. Relatedly, a dual point of view, looking at the correlations as linear functionals on the space of fields, allows one to bridge the classical thermodynamical point of view of statistical mechanics, in terms of Gibbs measures, with the point of view of CFT correlations, hence allowing one to view the Virasoro algebra as an action on space of measures. The main idea that emerges is: *the whole operator content and algebraic structure of certain CFTs can be explicitly constructed at the lattice level, and hence given a probabilistic meaning*.

The dual point of view of realizing the Virasoro algebra consists in looking at correlation functionals $\mu$ defined by ${{\mathcal {O}}}\mapsto \big \langle \, { {{\mathcal {O}}}(0) \prod {{\mathcal {O}}}_{j}(z_{j}) } \, \big \rangle _{{\Omega }}$, defined for any data of a domain ${\Omega }$, boundary conditions, with insertions ${{\mathcal {O}}}_{j}(z_{j})$ at $z_{j}\ne 0$, and in defining an adjoint (contragredient) action on such functionals, by defining ${\mathsf {L}}_{n}^{\dagger } \mu \left( {{\mathcal {O}}}\right) := \mu \left( {\mathsf {L}}_{-n}{{\mathcal {O}}}\right)$. Our main result yields a lattice analogue of this, as follows. Consider a sequence of discrete domains $\left( {\Omega }_{{\delta }_{k}}\right) _{k}$ with mesh sizes ${\delta }_{k}=1/k$, together with boundary conditions and insertions of lattice local fields ${{\mathcal {O}}}_{j}^{{\delta }_{k}}(z_{j})$ at points $z_{j}\ne 0$. We can then form the *sequence* of correlation functionals $\left( \mu _{k}\right) _{k}$ defined by ${{\mathcal {O}}}^{{\delta }} \mapsto {\mathbb {E}}_{{\Omega }_{{\delta }_{k}}} \left[ { {{\mathcal {O}}}^{{\delta }_{k}}(0) \prod {{\mathcal {O}}}_{j}^{{\delta }_{k}} (z_{j}) } \right]$ (for each ${{\mathcal {O}}}^{{\delta }}$, $\mu _{k}{{\mathcal {O}}}^{{\delta }}$ is defined for large enough *k*). The dual action gives rise to the sequence $\left( {\mathsf {L}}_{n}^{\dagger }\mu _{k}\right) _{k}$ by ${\mathsf {L}}_{n}^{\dagger }\mu _{k}({{\mathcal {O}}}^{{\delta }}) := \mu _{k}\left( {\mathsf {L}}_{-n}{{\mathcal {O}}}^{{\delta }}\right)$, where ${\mathsf {L}}_{-n}{{\mathcal {O}}}^{{\delta }}$ is the Virasoro action on the lattice local field ${{\mathcal {O}}}^{{\delta }}$ (for each ${{\mathcal {O}}}^{{\delta }}$ and $n\in {\mathbb {Z}}$, this is defined for large enough *k*). This is a natural generalization of the Gibbs measure, where instead of just looking at the (unnormalized) limits of $\mu _{k}$ as $k \rightarrow \infty$ (which is the definition of a Gibbs measure), one looks at the entire sequence itself (or more precisely, its tail).^4^ This point of view naturally bridges the Gibbs measure picture with the one of CFT.^5^

In [GHP19], a conjecture linked the local field picture of the Ising model with the operator content of the corresponding CFT: each lattice local field is conjectured to converge, with some proper normalization, to a CFT local field, and all CFT local fields can be obtained as such limits. The second part of this conjecture is particularly interesting as it allows one to give a probabilistic meaning to the operator content of the CFTs. This seems in particular to make sense of the operator content of the massive field theories emerging from perturbed CFTs (e.g. the one describing the critical Ising model with an infinitesimal magnetic field), where the axiomatic formalism breaks down. Our main result is a key step for establishing this second part: the operator content of the Ising CFT consists of descendants of three primary fields (the identity, the spin and the energy). Since the correlations of these fields have been established to converge to their continuous counterparts (the identity is trivial, see [CHI15] for the spin and [Hon10] for the energy), it remains to prove that the lattice descendants, as constructed by our main result, indeed converge.

In a subsequent paper, this will be proven, by combining the results of the present paper with the upcoming paper [CHI19], where it is proven that multipoint correlations of spins, energies and fermions, taken at far apart points, converge. A deformation of the discrete contour integrals appearing in the present paper will indeed allow one to reduce the correlations of any lattice descendant field, to those of the spin, energy and fermions and hence yield the result.

### Organization of the paper

In Sect. 2, we introduce the discrete complex analytic tools we need, in particular discrete holomorphic functions, discrete contours integrals, lattice integer and half-integer monomials. The proofs of the statements of this section are postponed to Sect. 5.

In Sect. 3, we introduce the relevant objects for the lattice models that we consider, in particular the lattice local fields, the discrete Gaussian free field current and the Ising fermion. The technical proofs of this section, pertaining to the Ising fermion, are postponed to Sect. 6.

In Sect. 4, we combine the objects and results of Sects. 2 and 3 to define the Virasoro algebra actions, and hence prove the main theorem.

## Discrete Complex Analysis

In two dimensions, conformal symmetry is deeply linked to complex analysis. On the lattice level, the combinatorial structures of the models we consider in this paper are linked with *discrete* complex analysis and this is what has allowed for proofs of conformal invariance of their scaling limits.

### Lattices and discrete domains

**Fig. 1:**
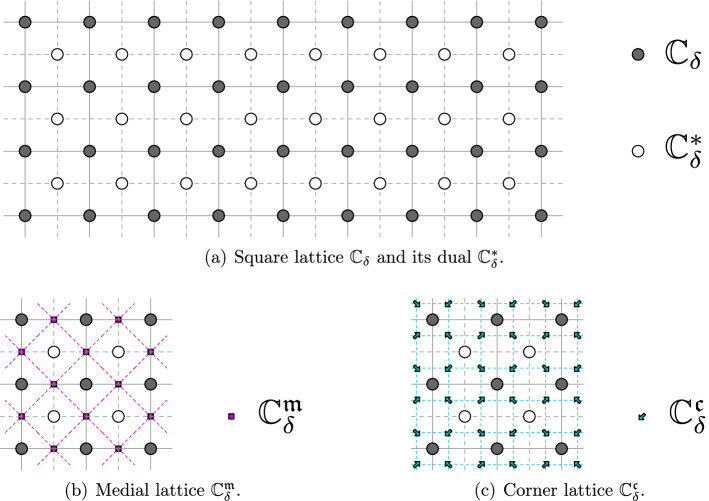
Illustrations of the lattices

We will work with a number of lattices associated with the square lattice ${\delta }{\mathbb {Z}}^2$ of mesh size ${\delta }$, and in particular use the following (see Fig. 1):

- Let $({{\mathbb {C}}_{{\delta }}},{{\mathcal {E}}_{{\delta }}})$ denote the *discrete complex plane*, i.e., the graph ${\delta }{\mathbb {Z}}^2$.
- Let $({{\mathbb {C}}_{{\delta }}^*}, {{\mathcal {E}}_{{\delta }}^*})$ be the *dual* of ${\delta }{\mathbb {Z}}^2$.
- Let $({{\mathbb {C}}_{{\delta }}^{\diamond }}, {{\mathcal {E}}_{{\delta }}^{\diamond }})$ be the *diamond graph* whose vertices are ${{\mathbb {C}}_{{\delta }}}\cup {{\mathbb {C}}_{{\delta }}^*}$ and with an edge connecting each pair of vertices at distance ${\delta }/\sqrt{2}$.
- Let $({{\mathbb {C}}_{{\delta }}^{{\mathfrak {m}}}}, {{\mathcal {E}}_{{\delta }}^{{\mathfrak {m}}}})$ be the *medial lattice* with respect to ${\delta }{\mathbb {Z}}^2$, with a vertex for each edge of ${{\mathcal {E}}_{{\delta }}}$; two medial vertices are adjacent if the corresponding edges share an endpoint.
- Let $({{\mathbb {C}}_{{\delta }}^{{\mathfrak {c}}}}, {{\mathcal {E}}_{{\delta }}^{{\mathfrak {c}}}})$ denote the *bi-medial lattice (corner lattice)*: each vertex of the bi-medial lattice is called a corner. A corner lies between a vertex and a dual vertex, and two corners are adjacent if they are at distance $\frac{{\delta }}{2}$ from each other. An edge $e \in {{\mathcal {E}}_{{\delta }}^{{\mathfrak {c}}}}$ of the bi-medial lattice lies between a vertex of the diamond lattice and a vertex of the medial lattice, denoted $e_{\diamond }$ and $e_{{\mathfrak {m}}}$.

Adjacency is denoted by $\sim$ on any of the above graphs: we denote $v \sim w$ if vertices *v* and *w* are the two endpoints of an edge. Moreover, for two points of different lattices, we still use the symbol $\sim$ to denote that the pair of points are nearest neighbors—e.g., for $z \in {{\mathbb {C}}_{{\delta }}^{\diamond }}$, $\zeta \in {{\mathbb {C}}_{{\delta }}^{{\mathfrak {m}}}}$ we have $z \sim \zeta$ if and only if $|z-\zeta | = \frac{{\delta }}{2}$.

Occasionally we work simultaneously with two of the above lattices, and for this purpose we use the shorthand notations ${{\mathbb {C}}_{{\delta }}^{{\mathfrak {m}}\diamond }}:= {{\mathbb {C}}_{{\delta }}^{{\mathfrak {m}}}}\cup {{\mathbb {C}}_{{\delta }}^{\diamond }}$ and ${{\mathbb {C}}_{{\delta }}^{{\mathfrak {c}}{\mathfrak {m}}}}:= {{\mathbb {C}}_{{\delta }}^{{\mathfrak {c}}}}\cup {{\mathbb {C}}_{{\delta }}^{{\mathfrak {m}}}}$.

### Discrete differential operators

**Fig. 2:**
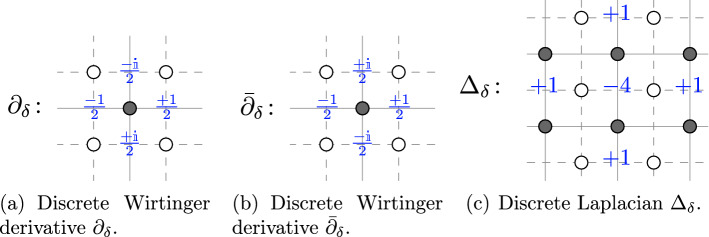
Discrete differential operators

Below, we introduce the lattice analogues of differential operators that we use. The coefficients of these finite difference operators ${\partial }_{{\delta }}, {{{\bar{\partial }}}}_{{\delta }}, {\Delta }_{{\delta }}$ are illustrated also in Fig. 2. Throughout the paper, whenever needed, we will extend functions *f* defined on subsets (subgraphs) ${{\Omega }}_{{\delta }}^{\diamond }$ of ${{\mathbb {C}}_{{\delta }}^{\diamond }}$ by setting $f|_{{{\mathbb {C}}_{{\delta }}^{\diamond }}\setminus {{\Omega }}_{{\delta }}^{\diamond }}\equiv 0$, and similarly for functions defined on subgraphs ${{\Omega }}_{{\delta }}^{{\mathfrak {m}}}$ of ${{\mathbb {C}}_{{\delta }}^{{\mathfrak {m}}}}$.

- For $f :{{\mathbb {C}}_{{\delta }}^{\diamond }}\rightarrow {\mathbb {C}}$, we define discrete *Wirtinger derivatives* ${\partial }_{{\delta }}f, {{{\bar{\partial }}}}_{{\delta }}f :{{\mathbb {C}}_{{\delta }}^{{\mathfrak {m}}}}\rightarrow {\mathbb {C}}$ by
  $$\begin{aligned} {\partial }_{{\delta }}f(z)&= \frac{1}{2} \left( f \big ( z+\frac{{\delta }}{2} \big ) - f\big ( z-\frac{{\delta }}{2} \big ) \right) - \frac{\mathbb {i}}{2} \left( f \big ( z+\frac{\mathbb {i}{\delta }}{2} \big ) - f \big ( z-\frac{\mathbb {i}{\delta }}{2} \big ) \right) \\ {{{\bar{\partial }}}}_{{\delta }}f(z)&= \frac{1}{2} \left( f \big ( z+\frac{{\delta }}{2} \big ) - f\big ( z-\frac{{\delta }}{2} \big ) \right) + \frac{\mathbb {i}}{2} \left( f \big ( z+\frac{\mathbb {i}{\delta }}{2} \big ) - f \big ( z-\frac{\mathbb {i}{\delta }}{2} \big ) \right) \end{aligned}$$
  and for $f :{{\mathbb {C}}_{{\delta }}^{{\mathfrak {m}}}}\rightarrow {\mathbb {C}}$, we define ${\partial }_{{\delta }}f,{{{\bar{\partial }}}}_{{\delta }}f :{{\mathbb {C}}_{{\delta }}^{\diamond }}\rightarrow {\mathbb {C}}$ by the same formulae.
- We define the discrete *Laplacian* as ${\Delta }_{{\delta }}= 4 \, {\partial }_{{\delta }}{{{\bar{\partial }}}}_{{\delta }}= 4 \, {{{\bar{\partial }}}}_{{\delta }}{\partial }_{{\delta }}$, so that for $f :{{\mathbb {C}}_{{\delta }}^{\diamond }}\rightarrow {\mathbb {C}}$ we have ${ {\Delta }_{{\delta }}f :{{\mathbb {C}}_{{\delta }}^{\diamond }}\rightarrow {\mathbb {C}}}$ given by
  $$\begin{aligned} {\Delta }_{{\delta }}f (z) = \sum _{x \in \left\{ \pm {\delta }, \pm \mathbb {i}{\delta } \right\} } f \big ( z+x \big ) -4 \, f \big ( z \big ) \end{aligned}$$
  and similarly for $f :{{\mathbb {C}}_{{\delta }}^{{\mathfrak {m}}}}\rightarrow {\mathbb {C}}$.
- A function *f* from ${{\mathbb {C}}_{{\delta }}^{\diamond }}$ or ${{\mathbb {C}}_{{\delta }}^{{\mathfrak {m}}}}$ to ${\mathbb {C}}$ is said to be *discrete holomorphic* (on a region of ${{\mathbb {C}}_{{\delta }}}$) if ${{{\bar{\partial }}}}_{{\delta }}f=0$ (on that region of ${{\mathbb {C}}_{{\delta }}}$). If *f* is discrete holomorphic, then it can be locally integrated, i.e., there exists *F* (at least locally defined) such that ${\partial }_{{\delta }}F=f$.

Note that we are not scaling the right-hand sides, so the continuum differential operators are approximated as ${\delta }\rightarrow 0$ by, e.g., ${\delta }^{-1} {\partial }_{{\delta }}\rightarrow {\partial }$ and ${\delta }^{-2} {\Delta }_{{\delta }}\rightarrow {\Delta }$.

### Discrete integration

**Fig. 3:**
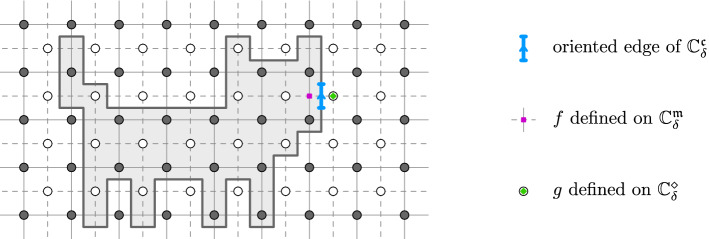
Discrete contour $\gamma$ on the corner lattice ${{\mathbb {C}}_{{\delta }}^{{\mathfrak {c}}}}$. Each (oriented) edge $\vec {e} \in \gamma$ of the contour separates a vertex $e_{\diamond } \in {{\mathbb {C}}_{{\delta }}^{\diamond }}$ of the diamond lattice from a vertex $e_{{\mathfrak {m}}} \in {{\mathbb {C}}_{{\delta }}^{{\mathfrak {m}}}}$ of the medial lattice

In the lattice setting, we will need to integrate products of two functions over discrete contours. By a *discrete contour*, we mean an oriented path $\gamma$ of edges on the corner lattice ${{\mathbb {C}}_{{\delta }}^{{\mathfrak {c}}}}$, see Fig. 3. For two functions $f :{{\mathbb {C}}_{{\delta }}^{{\mathfrak {m}}}}\rightarrow {\mathbb {C}}$ and $g :{{\mathbb {C}}_{{\delta }}^{\diamond }}\rightarrow {\mathbb {C}}$, we then define the *discrete contour integral* of *f* times *g* along $\gamma$ bywhere the sum is over all oriented edges $\vec {e} = (\vec {e}_- , \vec {e}_+)$ of $\gamma$, and where $e_{{\mathfrak {m}}}$ and $e_{\diamond }$ denote the medial and diamond vertices separated by $\vec {e}$. Note that the continuum contour integral approximation as ${\delta }\rightarrow 0$ again requires a scaling, .

If the discrete contour $\gamma$ is closed, we denote by $\mathrm {Int}[{\gamma }]$ the *interior of* $\gamma$, i.e., the set of points surrounded by $\gamma$. For closed counterclockwise discrete contours $\gamma$, we have the following discrete Stokes-like formula2.1In particular, if both *f* and *g* are discrete holomorphic in the symmetric difference $\mathrm {Int}[{\gamma }] \oplus \mathrm {Int}[{{\tilde{\gamma }}}]$ of two closed counterclockwise contours $\gamma , {\tilde{\gamma }}$, then we have the contour deformation property2.2Moreover, if $f,g :{{\mathbb {C}}_{{\delta }}^{{\mathfrak {m}}}}\rightarrow {\mathbb {C}}$ are discrete holomorphic in a lattice neighborhood of a closed integration contour $\gamma$ (i.e. ${{{\bar{\partial }}}}_{{\delta }}f(e_{\diamond }) = {{{\bar{\partial }}}}_{{\delta }}g(e_{\diamond }) = 0$ for any $e\in \gamma$), it is elementary to check (using Abel’s resummation) that we have the integration by parts formula2.3

### Discrete integer monomials

We now summarize basic facts about the discrete analogues $z\mapsto {z^{[{k}]}}$ of the monomial functions $z\mapsto z^{k}$ (for $k\in {\mathbb {Z}}$), leaving proofs for Sect. 5.5. These functions will later be used to construct the lattice counterparts of the holomorphic modes of discrete holomorphic fields both on ${{\mathbb {C}}_{{\delta }}^{\diamond }}$ and ${{\mathbb {C}}_{{\delta }}^{{\mathfrak {m}}}}$. In the following statement, we use the notation

$$\begin{aligned} {\mathbf {1}}_w(z) = {\left\{ \begin{array}{ll} 1 &{} \quad \text {if } z = w \\ 0 &{} \quad \text {if } z \ne w \end{array}\right. } \end{aligned}$$

for the Kronecker delta function.

#### Proposition 2.1

There exists a unique family of functions $({z^{[{k}]}})_{k \in {\mathbb {Z}}}$, defined on ${{\mathbb {C}}_{{\delta }}^{\diamond }}\cup {{\mathbb {C}}_{{\delta }}^{{\mathfrak {m}}}}$, for which the following properties hold:

1. For all $k \in {\mathbb {Z}}$, the function ${z^{[{k}]}}$ has the same 90 degree rotational symmetry around 0 as the continuous function $z \mapsto z^{k}$ does.
2. We have ${z^{[{0}]}} \equiv 1$ on ${{\mathbb {C}}_{{\delta }}^{\diamond }}\cup {{\mathbb {C}}_{{\delta }}^{{\mathfrak {m}}}}$.
3. For all $k\ge 0$, we have ${{{\bar{\partial }}}}_{{\delta }}{z^{[{k}]}} \equiv 0$. For all $k<0$, there exists $R>0$ such that for any $z\in {{\mathbb {C}}_{{\delta }}^{\diamond }}\cup {{\mathbb {C}}_{{\delta }}^{{\mathfrak {m}}}}$ with $\left| z\right| \ge R$, we have ${{{\bar{\partial }}}}_{{\delta }}{z^{[{k}]}} \equiv 0$.
4. For all $k \in {\mathbb {Z}}$, we have ${\partial }_{{\delta }}{z^{[{k}]}} = k \, {z^{[{k-1}]}}$.
5. We have  on ${{\mathbb {C}}_{{\delta }}^{\diamond }}$ and  on ${{\mathbb {C}}_{{\delta }}^{{\mathfrak {m}}}}$.
6. For all $k\le -1$, we have ${z^{[{k}]}} \rightarrow 0$ as $z \rightarrow \infty$.
7. For any fixed $z\in {{\mathbb {C}}_{{\delta }}^{\diamond }}\cup {{\mathbb {C}}_{{\delta }}^{{\mathfrak {m}}}}$ there exists $N\ge 0$ such that ${z^{[{k}]}} =0$ for all $k\ge N$.
8. As ${\delta }\rightarrow 0$, we have that ${\delta }^{k} {z^{[{k}]}}$ (extended, e.g., by linear interpolation) converges to the function $z \mapsto z^{k}$ uniformly on compact sets for $k\ge 0$ and uniformly away from the origin for $k<0$.
9. For any $k,\ell \in {\mathbb {Z}}$, we have  if $\gamma$ is a sufficiently large closed counterclockwise contour surrounding the origin.
10. Setting
  $$\begin{aligned} {z_{{\mathfrak {m}}}^{\{{k}\}}} := \frac{1}{4}\sum _{x\in \left\{ \pm 1, \pm \mathbb {i} \right\} } { \Big ( z-\frac{{\delta }x}{2} \Big )_{\diamond }^{[{k}]}} \end{aligned}$$
  for each $k \in {\mathbb {Z}}$, we have for all $k,\ell \in {\mathbb {Z}}$ if $\gamma$ is a sufficiently large closed counterclockwise contour surrounding the origin.

#### Proof

See Sect. 5.5. $$\square $$

### Discrete half-integer monomials

In this subsection we discuss discrete analogues $z\mapsto {z^{[{p}]}}$ to the functions $z\mapsto z^{p}$ for *half-integer* exponents $p \in {\mathbb {Z}}+ \frac{1}{2}$. As is the case for their continuous analogues, these functions are not naturally defined on ${{\mathbb {C}}_{{\delta }}}$, but rather on the *double cover* of ${{\mathbb {C}}_{{\delta }}}$, ramified at 0, denoted ${\left[ {{\mathbb {C}}_{{\delta }}},0 \right] }$. Above each vertex of ${{\mathbb {C}}_{{\delta }}}\setminus \left\{ 0\right\}$, there are now two vertices $v_{1},v_{2} \in {\left[ {{\mathbb {C}}_{{\delta }}}, 0 \right] }$, each one with a well-defined square root $\sqrt{v}_{1}=-\sqrt{v_{2}}$. For a vertex of ${\left[ {{\mathbb {C}}_{{\delta }}}, 0 \right] }$, there is a unique vertex of ${{\mathbb {C}}_{{\delta }}}\setminus \left\{ 0\right\}$ called the *base point*, and another vertex of ${\left[ {{\mathbb {C}}_{{\delta }}}, 0 \right] }$ called *the point on the opposite sheet*. Two vertices $v,w \in {\left[ {{\mathbb {C}}_{{\delta }}}, 0 \right] }$ are *adjacent* if their respective base points are adjacent and they are on the same sheet (i.e. $\Re {\mathfrak {e}}\left( \sqrt{v}/\sqrt{w}\right) >0$): the graph ${\left[ {{\mathbb {C}}_{{\delta }}}, 0 \right] }$ is hence connected. We define analogously dual vertices, medial vertices, and diamond vertices, and denote the relevant sets ${\left[ {{\mathbb {C}}_{{\delta }}^*}, 0 \right] }$, ${\left[ {{\mathbb {C}}_{{\delta }}^{{\mathfrak {m}}}}, 0 \right] }$ and ${\left[ {{\mathbb {C}}_{{\delta }}^{\diamond }}, 0 \right] }$, respectively, and we continue to denote adjacency by $\sim$. For a simple path $\lambda \subset {{\mathbb {C}}_{{\delta }}}\setminus \left\{ 0 \right\}$, we say that two points $a,b \in {\left[ {{\mathbb {C}}_{{\delta }}}, 0 \right] }$ with base points $a_{0},b_{0}\in \lambda$ are *on the same sheet* *of* $\lambda$ if following the square root branch along $\lambda$ one gets from $\sqrt{a}$ to $\sqrt{b}$.

For a function $f :{\left[ {{\mathbb {C}}_{{\delta }}^{\diamond }}, 0 \right] } \rightarrow {\mathbb {C}}$, we will always set $f(0) := 0$ and define ${\partial }_{{\delta }}f, {{{\bar{\partial }}}}_{{\delta }}f : {\left[ {{\mathbb {C}}_{{\delta }}^{{\mathfrak {m}}}}, 0 \right] } \rightarrow {\mathbb {C}}$ in the natural manner (i.e. taking $z\pm \frac{ {\delta }}{2},z\pm \mathbb {i}\frac{ {\delta }}{2}$ on the same sheet as *z*). We say that such a function has monodromy $-1$ around 0 if its values at points on opposite sheets are opposite (e.g. the square root function has $-1$ monodromy), and we say that it is single-valued if the values are equal.

The following lemma is easily verified.

#### Lemma 2.2

Let $f :{\left[ {{\mathbb {C}}_{{\delta }}^{{\mathfrak {m}}}}, 0 \right] } \rightarrow {\mathbb {C}}$ and $g :{\left[ {{\mathbb {C}}_{{\delta }}^{\diamond }}, 0 \right] } \rightarrow {\mathbb {C}}$ be two functions with monodromy $-1$ around 0. Then the function on the bi-medial edges $e\mapsto f(e_{{\mathfrak {m}}})g(e_{\diamond })$ is single-valued.

The existence, uniqueness and basic properties of the discrete half-integer monomials are summarized in the following proposition.

#### Proposition 2.3

There exists a unique family of functions $\left( {z^{[{p}]}} \right) _{p\in {\mathbb {Z}}+\frac{1}{2}}$ defined on the double cover of ${{\mathbb {C}}_{{\delta }}^{\diamond }}\cup {{\mathbb {C}}_{{\delta }}^{{\mathfrak {m}}}}$ ramified at 0 for which the following statements hold:

1. For all *p*, the function $${z^{[{p}]}} $$ has the same 90 degree rotational symmetry around 0 as the continuous function $$z\mapsto z^{p}$$ does.
2. ${z_{{\mathfrak {m}}}^{[{-\frac{1}{2}}]}}$ is given by Definition 5.5.
3. ${z_{{\mathfrak {m}}}^{[{\frac{1}{2}}]}}$ is given by Definition 5.11.
4. For all $p\ge \frac{1}{2}$ and $z \in {\left[ {{\mathbb {C}}_{{\delta }}}^{{\mathfrak {m}}\diamond }, 0 \right] }$, we have ${{{\bar{\partial }}}}_{{\delta }}{z^{[{p}]}} =0$. For each $p<0$, there exists $K>0$ such that ${{{\bar{\partial }}}}_{{\delta }}{z^{[{p}]}} = 0$ for all $z \in {\left[ {{\mathbb {C}}_{{\delta }}}^{{\mathfrak {m}}\diamond }, 0 \right] }$ with $\left| z\right| \ge K$.
5. For all $p \in {\mathbb {Z}}+ \frac{1}{2}$, we have ${\partial }_{{\delta }}{z^{[{p}]}} = p {z^{[{p-1}]}}$.
6. For all $p\le -\frac{1}{2}$, we have ${z^{[{p}]}} \rightarrow 0$ as $z \rightarrow \infty$.
7. For any fixed $z\in {{\mathbb {C}}_{{\delta }}^{\diamond }}\cup {{\mathbb {C}}_{{\delta }}^{{\mathfrak {m}}}}$ there exists $N\ge 0$ such that ${z^{[{p}]}} =0$ for all $p\ge N$.
8. As ${\delta }\rightarrow 0$, we have that ${\delta }^{p} {z^{[{p}]}}$ converges to the function $z\mapsto z^{p}$ uniformly on compact sets for $p\ge 0$ and uniformly away from the origin for $p<0$.
9. For any $p,q\in {\mathbb {Z}}+ \frac{1}{2}$, we have  if $\gamma$ is a sufficiently large closed counterclockwise contour surrounding the origin.
10. Setting
  $$\begin{aligned} {z_{{\mathfrak {m}}}^{\{{p}\}}} := \frac{1}{4}\sum _{x\in \left\{ \pm 1,\pm \mathbb {i}\right\} }\left( z-\frac{ {\delta }x}{2}\right) _{\diamond }^{[p]} , \end{aligned}$$
  we have for all $p,q \in {\mathbb {Z}}+ \frac{1}{2}$ if $\gamma$ is a sufficiently large closed counterclockwise contour surrounding the origin.

#### Proof

See Sect. 5.5. $$\square $$

#### Remark 2.4

It is possible to prove that the properties 1, 4, 5, together with ${z^{[{-1/2}]}} {z^{[{1/2}]}} \rightarrow 1$ as $z \rightarrow \infty$ imply the other ones.

## Gaussian Free Field and Ising Model

### Lattice models and field theory

As discussed in the introduction, a lattice model ${\mathcal {M}}$ associates to a discretization ${{\Omega }}_{{\delta }}\subset {{\mathbb {C}}_{{\delta }}}$ of a domain ${\Omega }\subset {\mathbb {C}}$ a random field ${\phi }_{{\delta }} :{{\Omega }}_{{\delta }}\rightarrow {\mathbb {C}}$ living on the discrete domain, i.e., a collection of (complex valued) random variables ${\phi }_{{\delta }}(z)$ indexed by the vertices *z* of the discrete domain. We now introduce (as in [GHP19]) a lattice model analogue to the fundamental notion of local field in Conformal Field Theory. Informally, the value of a lattice local field at a point *z* is the output of a translation invariant rule applied to the values that ${\phi }_{{\delta }}$ takes on a fixed finite neighborhood of *z*. For this, we assume furthermore that the field ${\phi }_{{\delta }}$ is extended to the complement ${{\mathbb {C}}_{{\delta }}}\setminus {{\Omega }}_{{\delta }}$ of the domain in some prescribed way.

#### Definition 3.1

(*Lattice local field*). Fix a lattice model ${\mathcal {M}}$. For $V \subset {\mathbb {Z}}^2$ a finite subset and $F :{\mathbb {C}}^{V} \rightarrow {\mathbb {C}}$ a polynomial function, the random fields given by

$$\begin{aligned} {{\mathcal {O}}}_{ {\delta }}(z) = F \left[ \Big ({\phi }_{ {\delta }} (z + x {\delta }) \Big )_{x\in V}\right] \end{aligned}$$

(for all possible choices of the discrete domain ${{\Omega }}_{{\delta }}\subset {{\mathbb {C}}_{{\delta }}}$ and of boundary conditions) constitute a (polynomial) lattice local field for the model ${\mathcal {M}}$. We denote by ${\mathfrak {F}}^\mathrm {loc}_{{\mathcal {M}}}$ the ${\mathbb {C}}$-vector space of such lattice local fields.

#### Remark 3.2

The condition that *F* is polynomial does not entail any loss of generality in the case of the Ising model. For the GFF, on the other hand, more general fields (e.g. $L^{2}$, such as exponentials) could be handled by density.

Examples of local fields are the field ${\phi }_{ {\delta }}$ itself, its square ${\phi }_{ {\delta }}^{2}$, its lattice derivative ${\phi }_{ {\delta }}\left( \cdot - {\delta }\right) -{\phi }_{ {\delta }}$, the product ${\phi }_{ {\delta }}( \cdot ) {\phi }_{ {\delta }}\left( \cdot + {\delta }\right)$, etc. The correlations of lattice local fields are simply defined by taking the expectation with respect to the measure of the model.

For critical lattice models such as the Gaussian free field and the Ising model, it is natural to:

- expect that every lattice local field converges to some CFT local field;
- expect that every CFT local field can be recovered as a limit of a suitably chosen lattice local field.

This convergence should hold in the sense that the (suitably renormalized) correlations of the lattice local fields converge to those of the CFT local fields, when taken at far apart points: fields in QFT are *defined* by their correlations. As a result, fields with the same correlations should be identified. This motivates the following:

#### Definition 3.3

(*Null field*). A lattice local field ${{\mathcal {O}}}_{ {\delta }}$ is called *null* (for a given model ${\mathcal {M}}$) if its correlations against any other lattice local fields vanish (for that model) as soon as the domain is large enough and the other insertions are far enough from *z*, i.e., there exists $R>0$ such that if *z* is at distance at least ${\delta }R$ from $z_{1}, \ldots , z_{n}$ and from ${{\mathbb {C}}_{{\delta }}}\setminus {{\Omega }}_{{\delta }}$, then we have

$$\begin{aligned} {\mathbb {E}}_{{{\Omega }}_{{\delta }}}\left[ {{\mathcal {O}}}_{ {\delta }}(z) {\phi }_{ {\delta }}(z_{1}) \cdots {\phi }_{ {\delta }}(z_{n}) \right] = 0 . \end{aligned}$$

Two lattice local fields are said to be *(correlation-)equivalent* if their difference is null. The subspace of null fields within the space of all local fields of a model is denoted by ${\mathfrak {F}}^\mathrm {null}_{{\mathcal {M}}}\subset {\mathfrak {F}}^\mathrm {loc}_{{\mathcal {M}}}$.

A more precise formulation of the conjectural correspondence of lattice local fields to CFT local fields is:

- we expect that for any local fields ${{\mathcal {O}}}_{1}, \ldots , {{\mathcal {O}}}_{n}$ of the CFT describing the scaling limit of the lattice model in question, there exist lattice local fields ${{\mathcal {O}}}_{1}^{ {\delta }}, \ldots , {{\mathcal {O}}}_{n}^{ {\delta }} \in {\mathfrak {F}}^\mathrm {loc}_{{\mathcal {M}}}$ and scaling dimensions $D_{1}, \ldots , D_{n} \in [0,\infty )$ (with each $D_{i}$ and ${{\mathcal {O}}}_{i}^{ {\delta }}$ depending on ${{\mathcal {O}}}_{i}$ only) such that if $z_{j}^{ {\delta }} \rightarrow z_{j}$ as ${\delta }\rightarrow 0$ (with $z_1 , \ldots , z_n$ distinct), we have
  $$\begin{aligned} \frac{1}{{\delta }^{\sum _{i=1}^{n}D_{i}}} {\mathbb {E}}_{{{\Omega }}_{{\delta }}}\left[ {{\mathcal {O}}}_{1}^{ {\delta }} \left( z_{1}^{{\delta }}\right) \cdots {{\mathcal {O}}}_{n}^{{\delta }} \left( z_{n}^{{\delta }}\right) \right] \underset{{\delta }\rightarrow 0}{\longrightarrow } \big \langle \, { {{\mathcal {O}}}_{1}(z_{1}) \cdots {{\mathcal {O}}}_{n}(z_{n}) } \, \big \rangle _{{\Omega }}. \end{aligned}$$

The construction of the present paper is a decisive tool to establish this conjecture for the discrete Gaussian free field and the Ising model. In particular, it gives an explicit way to construct the lattice precursors of all the Ising CFT descendant fields (*a fortiori*, since the algebraic structure of the descendant fields is already present at the lattice level).

### Discrete gaussian free field

The discrete Gaussian free field (dGFF) on a (finite) discrete domain ${{\Omega }}_{{\delta }}\subset {{\mathbb {C}}_{{\delta }}}$ (with Dirichlet boundary conditions) is a random field ${\varphi }:{{\mathbb {C}}_{{\delta }}}\rightarrow {\mathbb {R}}$ with ${\varphi }\big |_{{{\mathbb {C}}_{{\delta }}}\setminus {{\Omega }}_{{\delta }}}\equiv 0$ and density proportional to $\exp \big ( -\frac{1}{16\pi } E\left[ {\varphi }\right] \big )$, where the discrete Dirichlet energy *E* is defined by $E\left[ {\varphi }\right] :=\sum _{x\sim y}\left( {\varphi }\left( x\right) -{\varphi }\left( y\right) \right) ^{2}$. Equivalently, the dGFF ${\varphi }$ is a centered Gaussian field with covariance given by a multiple of the discrete Laplacian Green’s function of ${{\Omega }}_{{\delta }}$ with 0 boundary conditions:

$$\begin{aligned} {\mathbb {E}}\! \left[ { {\varphi }(z) {\varphi }(w) } \right] = 8 \pi \; {\mathbf {G}}_{{{\Omega }}_{{\delta }}}(z,w) , \end{aligned}$$

where the Green’s function is determined by ${\Delta }_{{\delta }}{\mathbf {G}}_{{{\Omega }}_{{\delta }}}(\cdot ,w) = - {\mathbf {1}}_w(\cdot )$ and ${\mathbf {G}}_{{{\Omega }}_{{\delta }}}(z,w) = 0$ unless $z,w \in {{\Omega }}_{{\delta }}$. The dGFF is a natural discretization of the continuous Gaussian free field on ${\Omega }$ (which is a random generalized function ${\Omega }\rightarrow {\mathbb {R}}$). Like any centered Gaussian field, the dGFF satisfies the bosonic Wick’s formula:

$$\begin{aligned} {\mathbb {E}}\! \left[ { {\varphi }(x_{1}) \cdots {\varphi }(x_{2n}) } \right] = \sum _{\left\{ \ell _{j},r_{j}\right\} } \prod _{j=1}^{n} {\mathbb {E}}\! \left[ { {\varphi }(x_{\ell _{j}} ) {\varphi }(x_{r_{j}}) } \right] , \end{aligned}$$

where the sum is over all pairings $\left\{ \ell _{1},r_{1}\right\} , \ldots , \left\{ \ell _{n},r_{n}\right\}$ of $\left\{ 1, \ldots ,2n \right\}$. The dGFF is a discrete harmonic field in the following sense.

#### Lemma 3.4

We have

$$\begin{aligned} {\mathbb {E}}\! \left[ { ({\Delta }_{{\delta }}{\varphi }) (x) \prod _{j=1}^{n} {\varphi }(x_{j}) } \right] = \sum _{i=1}^n (-8 \pi \, {\mathbf {1}}_{x_i}(x) ) \times {\mathbb {E}}\! \left[ { \prod _{j\ne i} {\varphi }(x_{j}) } \right] \end{aligned}$$

and in particular ${\mathbb {E}}\! \left[ { ({\Delta }_{{\delta }}{\varphi }) (x) \prod _{j=1}^{n} {\varphi }(x_{j}) } \right] = 0$ for $x \in {{\Omega }}_{{\delta }}\setminus \left\{ x_{1}, \ldots , x_{n} \right\}$.

#### Proof

This follows directly from Wick’s formula and the covariance being $8\pi$ times the discrete Laplacian Green’s function. $\square$

In particular, it follows from Lemma 3.4 that the discrete Laplacian of the discrete Gaussian free field is a null local field in the sense of Definition 3.3: ${\Delta }_{{\delta }}{\varphi }\in {\mathfrak {F}}^\mathrm {null}_{{\mathcal {G}}}$.

One of the most natural lattice local fields associated with the dGFF is the following *lattice holomorphic current* ${J}:{{\mathbb {C}}_{{\delta }}^{{\mathfrak {m}}}}\rightarrow {\mathbb {C}}$. We first extend ${\varphi }$ to ${{\mathbb {C}}_{{\delta }}^{\diamond }}$ by setting it to zero outside of ${{\Omega }}_{{\delta }}$ and on the dual lattice. Then we may define the current as

$$\begin{aligned} {J}\left( z \right) = \mathbb {i}\, {\partial }_{{\delta }}{\varphi }(z) . \end{aligned}$$

As defined, the current is not exactly the discrete analogue of the continuous current, as it is purely real on midpoints of vertical edges and purely imaginary on midpoints of horizontal edges. This is however not important for our approach, as the objects we will build out of the current ${J}$ are contour integrals, which do approximate the continuous integrals.

We have that the current ${J}$ is discrete holomorphic in the sense of correlations.

#### Lemma 3.5

Let ${{\Omega }}_{{\delta }}\subset {{\mathbb {C}}_{{\delta }}}$ be a discrete domain, let $V\subset {{\Omega }}_{{\delta }}$ be a finite set and $F :{\mathbb {R}}^{V} \rightarrow {\mathbb {C}}$ a polynomial function. Then we have that the function $G :{{\Omega }}_{{\delta }}\rightarrow {\mathbb {C}}$ defined by $G(z) := {\mathbb {E}}\! \left[ { F\big ({\varphi }\big |_{V}\big ) {J}(z) } \right]$ is discrete holomorphic for *z* away from ${{\mathbb {C}}_{{\delta }}}\setminus {{\Omega }}_{{\delta }}$ and from *V*.

#### Proof

This directly follows from the harmonicity of the dGFF since ${{{\bar{\partial }}}}_{{\delta }}{\partial }_{{\delta }}= \frac{1}{4} {\Delta }_{{\delta }}$. $\square$

The assumption that *F* is polynomial is chosen as it is general enough for our purposes, and specific enough so that the integrals exist.

At coinciding points, the lattice current has singularities, yielding nonzero contour integrals. The following elementary lemma will in particular be most useful:

#### Lemma 3.6

Let ${{\Omega }}_{{\delta }}\subset {{\mathbb {C}}_{{\delta }}}$ be a discrete domain, let $V\subset {{\Omega }}_{{\delta }}$ be a finite set and $F :{\mathbb {R}}^{V} \rightarrow {\mathbb {C}}$ be a polynomial function. Let $w\in {{\mathbb {C}}_{{\delta }}^{{\mathfrak {m}}}}$ be a point away from *V*. Consider the dGFF on a domain ${{\Omega }}_{{\delta }}$ that includes a neighborhood of *w*. Then for any closed counterclockwise contour $\gamma$ such that $w\in \mathrm {Int}[{\gamma }]$, $\mathrm {Int}[{\gamma }]\subset {{\Omega }}_{{\delta }}$ and $\mathrm {Int}[{\gamma }]\cap V = \emptyset$, and any function $f :{{\mathbb {C}}_{{\delta }}^{\diamond }}\rightarrow {\mathbb {C}}$ that is discrete holomorphic on $\mathrm {Int}[{\gamma }]$ we have

#### Proof

Observe first that for any $x\in {{\mathbb {C}}_{{\delta }}^{\diamond }}$ that is adjacent to *w*, Stokes’ formula (2.1) combined with Wick’s formula for the dGFF ${\varphi }$ with the explicit covariance $8 \pi \, {\mathbf {G}}_{{{\Omega }}_{{\delta }}}$ yieldBy taking a linear combination of the above over the four $x\in {{\mathbb {C}}_{{\delta }}^{\diamond }}$ adjacent to *w*, the assertion of the lemma follows. $\square$

### Ising model

We consider the Ising model on finite square grid domains ${{\Omega }}_{{\delta }}\subset {{\mathbb {C}}_{{\delta }}}$, and we allow for general boundary conditions. The boundary conditions are implemented by a choice of a fixed configuration ${\overline{{\sigma }}} :{{\mathbb {C}}_{{\delta }}}\setminus {{\Omega }}_{{\delta }}\rightarrow \left\{ -1,0,+1 \right\}$ outside the domain, and the sample space of allowed configurations of the model is then

$$\begin{aligned} \left\{ {\sigma }:{{\mathbb {C}}_{{\delta }}}\rightarrow \left\{ -1,0,1 \right\} \; \Big | \; {\sigma }_x \in \left\{ -1,+1 \right\} \text { for }x \in {{\Omega }}_{{\delta }}\text {, and } {\sigma }_x = {\overline{{\sigma }}}_x \text { for }x \notin {{\Omega }}_{{\delta }} \right\} . \end{aligned}$$

The constant function ${\overline{{\sigma }}} \equiv +1$ is known as *plus boundary conditions*, the constant function ${\overline{{\sigma }}} \equiv -1$ as *minus boundary conditions*, and the constant function ${\overline{{\sigma }}} \equiv 0$ as *free boundary conditions*. Our general boundary conditions can thus be combinations of these. The energy of a configuration ${\sigma }$ is defined as

$$\begin{aligned} {\mathcal {H}}[{\sigma }] = - \sum _{x\sim y}{\sigma }_{x}{\sigma }_{y} , \end{aligned}$$

where the sum is over nearest neighbor pairs such that at least one of the vertices *x*, *y* belongs to the (finite) domain ${{\Omega }}_{{\delta }}$. Given an inverse temperature parameter $\beta >0$, the probability measure of the model assigns probability proportional to $e^{-{{\beta }}{\mathcal {H}}[{\sigma }] }$ to each allowed configuration ${\sigma }$. The critical value for ${{\beta }}$ that is of interest for CFT is ${{\beta }}_{\mathrm {cr.}}=\frac{1}{2}\ln \left( \sqrt{2}+1\right)$.

Among the most natural local fields of the Ising model are the spin field ${\varsigma }_{{\delta }}\left( x\right) :={\sigma }_{x}$ and the energy field ${\varepsilon }_{{\delta }}\left( x\right) := {\sigma }_{x}{\sigma }_{x+{\delta }}-\frac{\sqrt{2}}{2}$. A number of results about the convergence and conformal invariance of the correlations of ${\delta }^{-1/8} {\varsigma }_{{\delta }}$ and ${\delta }^{-1} {\varepsilon }_{{\delta }}$ as ${\delta }\rightarrow 0$ with various boundary conditions have been established in [CHI15, Hon10, HoSm13, CHI19].

#### Disorder operators

The connection between the Ising model and complex analysis is more involved than for the GFF: it involves non-local fields, i.e., objects which have a point of insertion, which are functions of the spin configuration, and which have correlations, but which cannot be represented as lattice local fields. The most basic non-local fields are the *disorder *operators.

##### Definition 3.7

By a *disorder line between p and* *q* we mean a simple path ${\varrho }$ on the dual lattice ${{\mathbb {C}}_{{\delta }}^*}$ with endpoints $p,q \in {{\mathbb {C}}_{{\delta }}^*}$. We denote this ${\varrho }: p \leftrightarrow q$. For an Ising configuration $\left( {\sigma }_{x}\right) _{x\in {{\mathbb {C}}_{{\delta }}}}$ define the *disorder energy* of ${\varrho }$ by ${{\mathcal {E}}}_{{\varrho }}[{\sigma }] = \sum _{x\sim y: \langle xy \rangle ^{*} \in {\varrho }} {\sigma }_{x}{\sigma }_{y}$. For a disorder line ${\varrho }$ between *p* and *q* we define the *disorder pair* $({\mu }_{p}{\mu }_{q})_{{\varrho }}$ as the random variable

3.1 $$\begin{aligned} ({\mu }_{p}{\mu }_{q})_{{\varrho }} = \exp \Big (-2{{\beta }}_{\mathrm {cr.}}{{\mathcal {E}}}_{{\varrho }}[{\sigma }] \Big ) . \end{aligned}$$

**Fig. 4:**
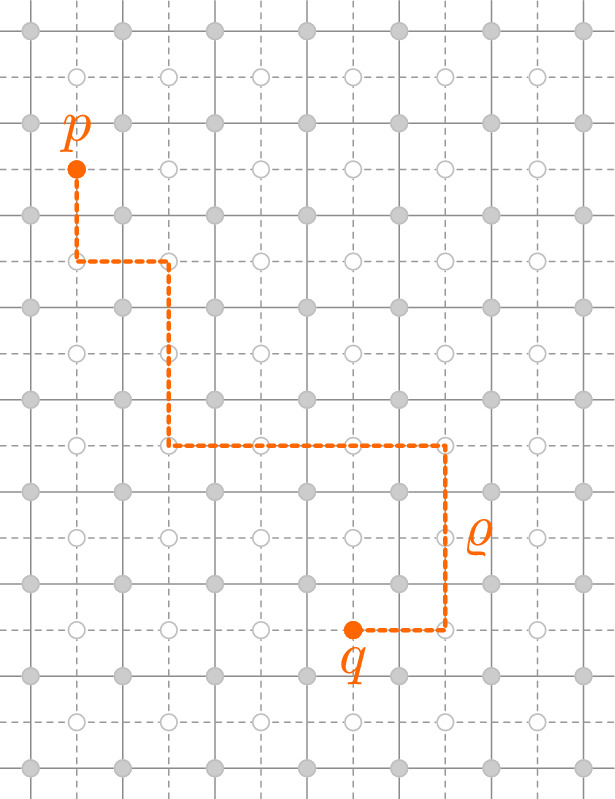
A disorder line ${\varrho }$ is a path between two dual vertices $p,q \in {{\mathbb {C}}_{{\delta }}^*}$

Note that for a fixed disorder line ${\varrho }$, a disorder pair $({\mu }_{p}{\mu }_{q})_{{\varrho }}$ defines a lattice local field of the Ising model, whereas a single disorder operator “${\mu }_p$” could not be defined as such (Fig. 4).

**Fig. 5:**
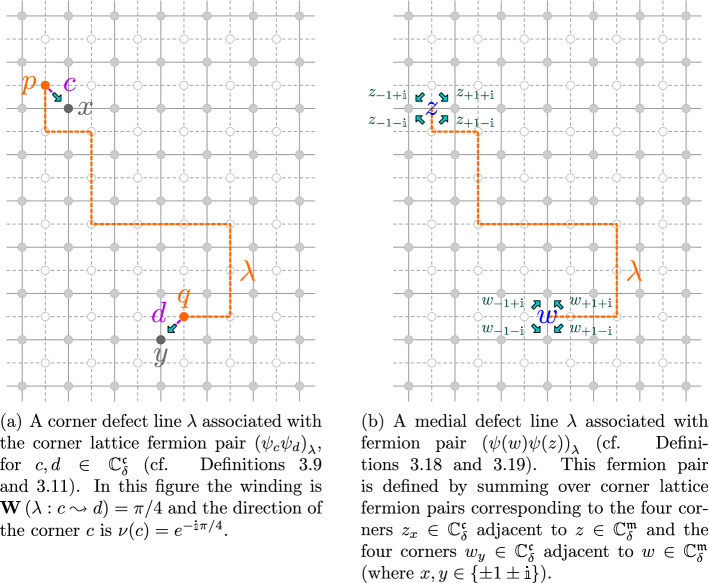
Corner lattice fermions and medial lattice fermions

Intuitively, reweighting a correlation by a disorder pair $({\mu }_{p}{\mu }_{q})_{{\varrho }}$, i.e., considering the reweighted measure

$$\begin{aligned} F \mapsto \frac{{\mathbb {E}}\! \left[ { ({\mu }_{p}{\mu }_{q})_{{\varrho }} \, F[{\sigma }] } \right] }{{\mathbb {E}}\! \left[ {({\mu }_{p}{\mu }_{q})_{{\varrho }} } \right] } , \end{aligned}$$

corresponds to an Ising model where the spins ‘pretend’ that their neighbors across ${\varrho }$ are equal to the opposite of their actual values. The following lemma (due to [KaCe71], see also [Dub11a, Dub11b, CCK17]) tells us that disorder pair correlations are essentially dependent on the endpoints of the path only (and hence they are sometimes called quasi-local fields) (Fig. 5).

##### Lemma 3.8

Let $\Gamma _{1},\Gamma _{2}$ be two collections of *k* disjoint disorder lines such that the sets of 2*k* endpoints of both collections are the same. Let $\Gamma _{1}\oplus \Gamma _{2}$ denote the collection of loops made of the symmetric difference of the sets of dual edges $\cup _{{\varrho }_{1}\in \Gamma _{1}} {\varrho }_{1}$ and $\cup _{{\varrho }_{2}\in \Gamma _{2}} {\varrho }_{2}$. Let $V\subset {{\mathbb {C}}_{{\delta }}}$ be a finite set. Consider the Ising model on a large enough domain ${{\Omega }}_{{\delta }}$, with arbitrary boundary conditions. We have

3.2 $$\begin{aligned} {\mathbb {E}}\! \left[ { \left( \prod _{v\in V} {\sigma }_{v} \right) \prod _{{\varrho }: p \leftrightarrow q \in \Gamma _{1}} ({\mu }_{p}{\mu }_{q})_{{\varrho }} } \right] = \left( -1\right) ^{{\mathcal {N}}} {\mathbb {E}}\! \left[ { \left( \prod _{v \in V}{\sigma }_{v} \right) \prod _{{\varrho }:p\leftrightarrow q \in \Gamma _{2}} ({\mu }_{p}{\mu }_{q})_{{\varrho }} } \right] ,\qquad \end{aligned}$$

where the ${\mathcal {N}}$ is the number of pairs $\left( v,\ell \right)$ where $v\in V$ and $\ell \in \Gamma _{1}\oplus \Gamma _{2}$ is a loop surrounding *v*.

##### Proof

**Fig. 6:**
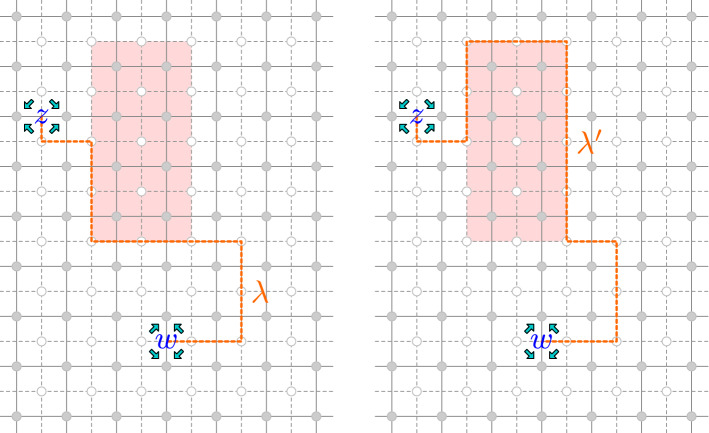
The defect line ${\lambda }'$ on the right is obtained from the defect line ${\lambda }$ on the left by performing the gauge transformation which flips the spins in the shaded area

**Fig. 7:**
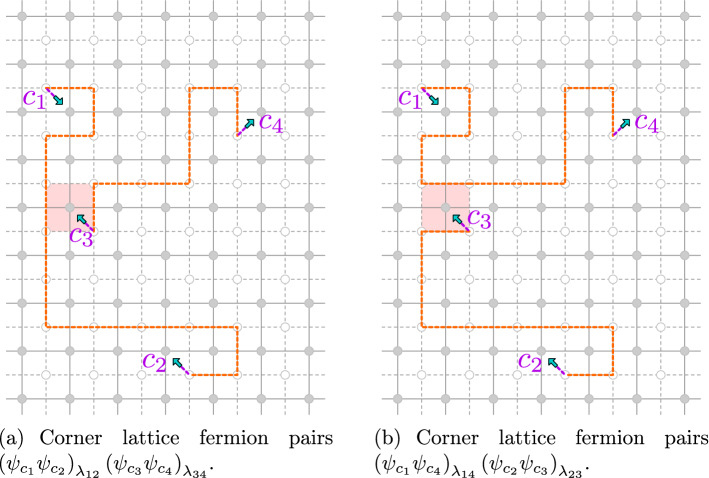
A gauge transformation involving two fermion pairs, and consisting of a flip of the spin at the center of the shaded square

For each loop $\ell \in \Gamma _{1}\oplus \Gamma _{2}$, let ${\mathsf {S}}_{\ell } :\left\{ \pm 1 \right\} ^{{{\Omega }}_{{\delta }}} \rightarrow \left\{ \pm 1 \right\} ^{{{\Omega }}_{{\delta }}}$ be the involution that flips all the spins contained inside of $\ell$ and leaves the other ones unchanged (see Figs. 6 and 7 in Sect. 3.3.2 for examples of similar gauge transformations). Let ${\mathsf {S}}$ denote the gauge transform consisting of composition of all the (commuting) ${\mathsf {S}}_{\ell }$ for $\ell \in \Gamma _{1}\oplus \Gamma _{2}$. For a configuration ${{\sigma }\in \left\{ \pm 1 \right\} ^{{{\Omega }}_{{\delta }}}}$, and $j=1,2$, consider

$$\begin{aligned} {\mathcal {H}}_{j}[{\sigma }] := {\mathcal {H}}[{\sigma }] + 2 \sum _{{\varrho }\in \Gamma _{j}} {{\mathcal {E}}}_{{\varrho }}[{\sigma }] . \end{aligned}$$

Proving (3.2) hence amounts to showing that

$$\begin{aligned} \sum _{{\sigma }\in \left\{ \pm 1 \right\} ^{{{\Omega }}_{{\delta }}}} \left( \prod _{v\in V} {\sigma }_{v}\right) e^{-{{\beta }}{\mathcal {H}}_{1}[{\sigma }] } = \left( -1\right) ^{{\mathcal {N}}} \sum _{{\widetilde{{\sigma }}}\in \left\{ \pm 1 \right\} ^{{{\Omega }}_{{\delta }}}} \left( \prod _{v\in V}{\widetilde{{\sigma }}}_{v}\right) e^{-{{\beta }}{\mathcal {H}}_{2}\left[ {\widetilde{{\sigma }}}\right] } , \end{aligned}$$

which simply follows by observing that if ${\widetilde{{\sigma }}}={\mathsf {S}}[{\sigma }]$, then $\prod _{v\in V}{\sigma }_{v} = \left( -1\right) ^{{\mathcal {N}}}\prod _{v \in V}{\widetilde{{\sigma }}}_{v}$ and ${\mathcal {H}}_{2}\left[ {\widetilde{{\sigma }}}\right] = {\mathcal {H}}_{1}[{\sigma }]$. $\square$

#### Corner lattice fermions

Informally, a fermion operator ${\psi }$ consists of a spin (living on the primal lattice ${{\mathbb {C}}_{{\delta }}}$) next to a disorder (living on the dual lattice ${{\mathbb {C}}_{{\delta }}^*}$); a natural location for a fermion is hence at a corner (between a vertex and and a dual vertex). Again, due to the non-locality of the disorder operator, we define correlations of pairs of fermions with a defect path between them; later, we show that only the sign of correlations is affected by the choice of the path.

##### Definition 3.9

Let *c* be a corner between $x \in {{\mathbb {C}}_{{\delta }}}$ and $p \in {{\mathbb {C}}_{{\delta }}^*}$ and let *d* be a corner between $y \in {{\mathbb {C}}_{{\delta }}}$ and $q \in {{\mathbb {C}}_{{\delta }}^*}$. We define a *corner defect line* ${\lambda }$ *with corner-ends* $c,d\in {{\mathbb {C}}_{{\delta }}^{{\mathfrak {c}}}}$ as the concatenation $\left[ cp\right] \oplus {\varrho }\oplus \left[ qd\right]$ of a disorder line ${\varrho }$ with endpoints *p*, *q* with the two *corner segments* $\left[ cp\right]$ and $\left[ qd\right]$. We denote ${\lambda }: c \leftrightarrow d$, and we call ${\varrho }: p \leftrightarrow q$ the *main part* of ${\lambda }$. We call *x*, *y* the *spin-ends* and *p*, *q* the *disorder-ends* of ${\lambda }$. We denote by ${\mathbf {W}}\left( {\lambda }:c\leadsto d\right)$ the cumulative angle of turns by ${\lambda }$ (also known as *winding*) traversed from *c* to *d*.

##### Remark 3.10

While the definition of ${\mathbf {W}}$ is the same as that in related works [CHI15, ChSm12, Hon10, HoSm13], the defect line is not the same object as the path appearing in the low-temperature expansion of the fermionic observables of these works. That path should interpreted as a line of frustration and should be viewed as a configuration-dependent object, unlike the defect line, which is fixed.

We now introduce the lattice fermion pair that we will work with, a complexification of that introduced by Kadanoff and Ceva [KaCe71], see also [Dub11a, Dub11b].

##### Definition 3.11

Let ${\lambda }$ be a corner defect line with corner-ends *c*, *d*, spin-ends *x*, *y* and disorder-ends *p*, *q*. Let

$$\begin{aligned} {\nu }(c) := \frac{x-p}{\left| x-p\right| } \in \big \{ e^{\pm \frac{\pi \mathbb {i}}{4}},e^{\pm \frac{3\pi \mathbb {i}}{4}} \big \} \end{aligned}$$

denote the direction of the corner *c*. We define the fermion pair $\left( {\psi }_{c}{\psi }_{d}\right) _{{\lambda }}$ as

##### Remark 3.12

The two-point correlation function of the corner lattice fermion coincides with the two-point observable defined in [GHP19], in the special case when $+$ boundary conditions are imposed and no other fields are inserted in the correlations. However, the definition of corner lattice fermion given above makes the nature of the fermion pair (with fixed defect line) as a local field (i.e., as a function of a finite number of spins) transparent and explicit.

Despite the apparent difference of rôle of *c* and *d* in the definition, the fermion pair is antisymmetric.

##### Lemma 3.13

Let ${\lambda }$ be a corner disorder line with corner-ends *c*, *d*. Then we have $\left( {\psi }_{c}{\psi }_{d}\right) _{{\lambda }} +\left( {\psi }_{d}{\psi }_{c}\right) _{{\lambda }} =0$.

##### Proof

It is elementary to check that , and the rest is unchanged. $\square$

Fixing the two corners *c*, *d*, we have that the dependence on ${\lambda }$ of $\left( {\psi }_{c}{\psi }_{d}\right) _{{\lambda }}$ is not due to local factors.

##### Lemma 3.14

Let ${\lambda },{\tilde{{\lambda }}}$ be two corner defect lines sharing the same corner-ends *c*, *d*. Let ${\lambda }\oplus {\tilde{{\lambda }}}$ denote the collection of loops of ${{\mathbb {C}}_{{\delta }}^*}$ made of the symmetric difference of ${\lambda }$ and ${\tilde{{\lambda }}}$. Let $V\subset {{\mathbb {C}}_{{\delta }}}$ be a finite subset. Consider the Ising model on a large enough ${{\Omega }}_{{\delta }}$, with arbitrary boundary conditions. We have

$$\begin{aligned} {\mathbb {E}}\! \left[ { \left( \prod _{v\in V}{\sigma }_{v} \right) \left( {\psi }_{c}{\psi }_{d}\right) _{{\lambda }} } \right] = \left( -1\right) ^{{\mathcal {N}}} \, {\mathbb {E}}\! \left[ { \left( \prod _{v\in V} {\sigma }_{v}\right) \left( {\psi }_{c}{\psi }_{d}\right) _{{\tilde{{\lambda }}}} } \right] , \end{aligned}$$

where ${\mathcal {N}}$ is the number of pairs $\left( v,\ell \right)$ where $v\in V$ and $\ell \in {\lambda }\oplus {\tilde{{\lambda }}}$ surrounds *v*.

##### Proof

The proof uses the same bijection as the proof of Lemma 3.8 (see Fig. 6). It is elementary to check that the  term in the definition of the fermion compensates for the change of the spins adjacent to the corners *c*, *d*. The rest behaves in the same manner, thus yielding the result. $\square$

The following lemma, which allows one to exchange defect lines between four fermions, will be instrumental in our construction:.

##### Lemma 3.15

Let $V\subset {{\mathbb {C}}_{{\delta }}}$ be a finite set and $F :\left\{ \pm 1 \right\} ^{V} \rightarrow {\mathbb {C}}$. Let $c_{1},c_{2},c_{3},c_{4}$ be distinct corners. For $i<j$, let ${\lambda }_{ij}:c_{i}\leftrightarrow c_{j}$ be corner defect lines which are disjoint when the indices have no overlap, i.e., ${\lambda }_{12}\cap {\lambda }_{34} = {\lambda }_{13}\cap {\lambda }_{24} = {\lambda }_{14} \cap {\lambda }_{23} = \emptyset$. If $\left( {\lambda }_{12}\oplus {\lambda }_{34}\right) \oplus \left( {\lambda }_{13} \oplus {\lambda }_{24}\right)$ and $\left( {\lambda }_{12}\oplus {\lambda }_{34}\right) \oplus \left( {\lambda }_{14} \oplus {\lambda }_{23}\right)$ do not contain loops surrounding any point of *V*, then for any large enough ${{\Omega }}_{{\delta }}$ (with arbitrary boundary conditions) we have

$$\begin{aligned} {\mathbb {E}}\! \left[ { F\left( {\sigma }|_{V}\right) \, \left( {\psi }_{c_{1}} {\psi }_{c_{2}}\right) _{{\lambda }_{12}} \left( {\psi }_{c_{3}} {\psi }_{c_{4}}\right) _{{\lambda }_{34}} } \right]&= - {\mathbb {E}}\! \left[ { F\left( {\sigma }|_{V}\right) \, \left( {\psi }_{c_{1}} {\psi }_{c_{3}}\right) _{{\lambda }_{13}} \left( {\psi }_{c_{2}} {\psi }_{c_{4}}\right) _{{\lambda }_{24}}} \right] \\&= {\mathbb {E}}\! \left[ { F\left( {\sigma }|_{V}\right) \, \left( {\psi }_{c_{1}} {\psi }_{c_{4}}\right) _{{\lambda }_{14}} \left( {\psi }_{c_{2}} {\psi }_{c_{3}} \right) _{{\lambda }_{23}} } \right] . \end{aligned}$$

##### Proof

Let us only prove the first equality (the other one is symmetric). For each loop $\ell \in \left( {\lambda }_{12}\oplus {\lambda }_{34} \right) \oplus \left( {\lambda }_{ 13} \oplus {\lambda }_{24}\right)$, which by assumption does not surround points of *V*, define the gauge transform ${\mathsf {S}}_{\ell }$ that flips the spins inside it as before. We can assume that $\ell$ is a loop that includes edges of ${\lambda }_{12}$, ${\lambda }_{34}$, ${\lambda }_{13}$ and ${\lambda }_{24}$: otherwise the gauge transform ${\mathsf {S}}_{\ell }$ just amounts to displacing a piece of an individual defect line, without exchanging the lines endpoints, and this case can be handled by an application of Lemma 3.14. By composing enough gauge transforms which do not affect the left-hand and right-hand side, we can actually assume that $\ell$ is a square surrounding only one spin, with its horizontal edges belonging to ${\lambda }_{12}$ and ${\lambda }_{34}$ and its vertical edges belonging to ${\lambda }_{13}$ and ${\lambda }_{24}$. It is then elementary to check that ${\mathsf {S}}_{\ell }$ affects the fermions in the desired way (see Fig. 7). $\square$

The above lemma generalizes to the following.

##### Proposition 3.16

Let $V\subset {{\mathbb {C}}_{{\delta }}}$ be finite. Let $c_{1},\ldots ,c_{2k}$ be distinct corners. Let

$$\begin{aligned} \Lambda =\left\{ {\lambda }_{1}:c_{1}\leftrightarrow c_{2},\ldots ,{\lambda }_{k}:c_{2k-1}\leftrightarrow c_{2k}\right\} \end{aligned}$$

be a collection of *k* disjoint corner defect lines and let ${\tilde{\Lambda }}$ be a collection of *k* disjoint corner defect lines ${\tilde{{\lambda }}}_{j}: c_{m_{j}}\leftrightarrow c_{n_{j}}$ linking the $c_{1},\ldots ,c_{2k}$ pairwise, with $m_{j}<n_{j}$. Let $\Lambda \oplus {\tilde{\Lambda }}$ the set of loops made of the symmetric difference ${\big (\cup {\lambda }_{j}\big ) \oplus \big (\cup {\tilde{{\lambda }}}_{j}\big )}$, and let ${\mathcal {N}}$ denote the number of pairs $\left( v,\ell \right)$ with $v\in V$ and $\ell \in \Lambda \oplus {\tilde{\Lambda }}$ surrounding *v*. Let ${\mathcal {C}}$ denote the number of crossings of the pair partition $\left\{ \left( m_{j},n_{j}\right) \right\}$ of $\left\{ 1, \ldots , 2k \right\}$, i.e. the number of pairs $j<k$ such that $m_{j}<m_{k}<n_{j}<n_{k}$. Consider the Ising model on a large enough domain ${{\Omega }}_{{\delta }}$ with arbitrary boundary conditions. We have

$$\begin{aligned}&{\mathbb {E}}\! \left[ { \left( \prod _{v\in V}{\sigma }_{v}\right) \big ( {\psi }_{c_{1}} {\psi }_{c_{2}} \big )_{{\lambda }_{1}} \cdots \big ( {\psi }_{c_{2k-1}} {\psi }_{c_{2k}} \big )_{{\lambda }_{k}}} \right] \\&\quad = \left( -1\right) ^{{\mathcal {N}}+{\mathcal {C}}} \; {\mathbb {E}}\! \left[ { \left( \prod _{v\in V} {\sigma }_{v} \right) \big ( {\psi }_{c_{m_{1}}} {\psi }_{c_{n_{1}}} \big )_{{\lambda }_{1}} \cdots \big ({\psi }_{c_{m_{k}}} {\psi }_{c_{n_{k}}} \big )_{{\lambda }_{k}} } \right] . \end{aligned}$$

##### Proof

This follows from Lemmas 3.14 and 3.15, by induction on *k*. $\square$

This then yields the following important proposition, which allows one to avoid specifying the defect paths:

##### Proposition 3.17

Let $V\subset {{\mathbb {C}}_{{\delta }}}$ be a finite and connected set and let ${{\Omega }}_{{\delta }}\supset V$ be a large enough domain. Consider the Ising model on ${{\Omega }}_{{\delta }}$ with arbitrary boundary conditions. Let ${\left[ {{\Omega }}_{{\delta }}, V \right] }$ denote the double cover of ${{\Omega }}_{{\delta }}\setminus V$ ramified around *V*. For $c_{1},\ldots ,c_{2k} \in {\left[ {{\Omega }}_{{\delta }}^{{\mathfrak {c}}}, V \right] }$ and any $F :\{ \pm 1 \}^{V} \rightarrow {\mathbb {C}}$, the correlation

$$\begin{aligned}&{\mathbb {E}}\! \left[ {F \big ( {\sigma }|_{V} \big ) \, {\psi }(c_{1}) \cdots {\psi }(c_{ 2k})} \right] \\&\quad := {\mathbb {E}}\! \left[ { F \big ( {\sigma }|_{V} \big ) \big ( {\psi }_{c_{1}} {\psi }_{c_{2}} \big )_{{\lambda }_{1}} \cdots \big ( {\psi }_{c_{2k-1}} {\psi }_{c_{2k}} \big )_{{\lambda }_{k}} } \right] \end{aligned}$$

is independent of the choice of ${\lambda }_{1}:c_{1}\leftrightarrow c_{2},\ldots ,{\lambda }_{k}:c_{2k-1}\leftrightarrow c_{2k}$, provided the ${\lambda }_{j}$’s stay away from *V* and that $c_{2j-1},c_{2j}$ are on the same sheet of ${\left[ {{\mathbb {C}}_{{\delta }}}, V \right] }$ when going along ${\lambda }_{j}$. The resulting correlation ${\mathbb {E}}\! \left[ { F\big ({\sigma }|_{V}\big ) \, {\psi }(c_{1}) \cdots {\psi }(c_{ 2k}) } \right]$ is totally antisymmetric with respect to permutations of the variables $c_{1},\ldots ,c_{2k}$. It is single-valued as a function of each $c_j$ if *F* is even and has $-1$ monodromy around *V* if *F* is odd.

##### Proof

By the Proposition 3.16, the only dependence on a path such as ${\lambda }_{1}$ is through its lift to the double cover (if we modify ${\lambda }_{1}$ by a symmetric difference of two loops both surrounding *V*, it does not change the correlations). If we modify ${\lambda }_{1}$ by a loop surrounding *V*, the correlation will change sign if *F* is odd and stay constant if *F* is even. The antisymmetry follows from Lemmas 3.13 and 3.14. $\square$

#### Discrete holomorphic fermions

We now introduce the discrete holomorphic fermions, which live on the medial lattice: informally they are simply the averages of the corner fermions taken at the four corners surrounding a medial vertex. At criticality, their correlations are discrete holomorphic (Proposition 3.22).

##### Definition 3.18

Let $w,z\in {{\mathbb {C}}_{{\delta }}^{{\mathfrak {m}}}}$ be medial vertices. We define a *medial defect line* ${\lambda }$ with medial-ends *w*, *z* as the concatenation $\left[ wp\right] \oplus {\varrho }\oplus \left[ qz\right]$ where $p,q \in {{\mathbb {C}}_{{\delta }}^*}$ are adjacent to *w*, *z* and ${\varrho }$ is a simple path on the dual lattice, called the *main part* of ${\lambda }$. We say that two (corner, medial) defect lines *differ only locally*, if their endpoints are either the same or neighbors and the main parts differ by at most the dual edges containing the endpoints.

Let us now introduce the key object: the discrete holomorphic fermion, which lives on the medial lattice (see Fig. 2b).

##### Definition 3.19

For $z \in {{\mathbb {C}}_{{\delta }}^{{\mathfrak {m}}}}$ and $x \in \left\{ \pm 1\pm \mathbb {i} \right\}$, let $z_{x} := z + \frac{{\delta }}{4} x$ denote the corner adjacent to *z* in direction *x*; for $w \in {{\mathbb {C}}_{{\delta }}^{{\mathfrak {m}}}}$ and $y \in \left\{ \pm 1\pm \mathbb {i} \right\}$ write $w_{y} := w+ \frac{{\delta }}{4} y$ analogously. Fix a medial defect line ${\lambda }$ with medial ends *z*, *w*, and let ${\lambda }_{yx}$ denote the corner defect lines with corner ends $w_{y}$ and $z_{x}$ such that the main parts of ${\lambda }_{yx}$ and ${\lambda }$ differ at most by the edges containing *w*, *z*. We define the *discrete holomorphic fermion pair* $\left( {\psi }(w) {\psi }(z) \right) _{{\lambda }}$ by

$$\begin{aligned} \left( {\psi }(w) {\psi }(z) \right) _{{\lambda }} = \frac{\pi }{8\sqrt{2}} \sum _{x,y \in \left\{ \pm 1 \pm \mathbb {i} \right\} } \left( {\psi }_{w_{y}} {\psi }_{z_{x}}\right) _{{\lambda }_{yx}}, \end{aligned}$$

where if $w_{y}=z_{x}$, we interpret  in Definition 3.11.

##### Remark 3.20

The correlations of $\left( {\psi }(w) {\psi }(z) \right) _{{\lambda }}$ taken in a domain with $+$ boundary conditions and without any other fields correspond to the observable $\sum _{\zeta ,\xi } \frac{\sqrt{\zeta }}{\sqrt{\xi }} f_{{{\Omega }}_{{\delta }}} \left( w^{\zeta } , z^{\xi } \right)$ of [Hon10], where the sum is taken over the possible orientations of the edges *e*(*w*) and *e*(*z*) .

The antisymmetry is naturally inherited from the corner-lattice fermion.

##### Lemma 3.21

For a medial defect line ${\lambda }$ with (distinct) medial-ends *w*, *z* we have that

$$\begin{aligned} \left( {\psi }(w) {\psi }(z) \right) _{{\lambda }} = - \left( {\psi }(z) {\psi }(w) \right) _{{\lambda }} . \end{aligned}$$

##### Proof

Straightforward from Lemma 3.13. $\square$

By Lemma 3.14 the correlations of this fermion pair are independent of the choice of the defect line ${\lambda }$, up to a sign. We thus omit the defect line from the notation, and consider the correlations defined on the appropriate double cover. A fundamental property of the correlations of $\left( {\psi }(w) {\psi }(z) \right)$ is their discrete holomorphicity apart from singularities when *w* and *z* coincide.

##### Proposition 3.22

Let ${{\Omega }}_{{\delta }}\subset {{\mathbb {C}}_{{\delta }}}$ be a discrete domain and let $V \subset {{\Omega }}_{{\delta }}$ be a connected set. Consider the Ising model on ${{\Omega }}_{{\delta }}$ with arbitrary boundary conditions. Let ${\left[ {{\Omega }}_{{\delta }}, V \right] }$ denote the double cover of ${{\Omega }}_{{\delta }}\setminus V$ ramified around *V*. Let $W \subset V$, let $w \in {\left[ {{\Omega }}_{{\delta }}^{{\mathfrak {m}}}, V \right] }$ and let $w^{*} \in {\left[ {{\Omega }}_{{\delta }}^{{\mathfrak {m}}}, V \right] }$ share the same base point, on the opposite sheet. Consider the function $H :{\left[ {{\Omega }}_{{\delta }}^{{\mathfrak {m}}}, V \right] } \rightarrow {\mathbb {C}}$ defined by

$$\begin{aligned} H(z) := {\mathbb {E}}\! \left[ { \left( \prod _{x\in W}{\sigma }_{x}\right) {\psi }(w) {\psi }(z) } \right] . \end{aligned}$$

Then for $\zeta \in {\left[ {{\Omega }}_{{\delta }}^{\diamond }, V \right] }$ away from $\partial {{\Omega }}_{{\delta }}, V$, we have

$$\begin{aligned}&{{{\bar{\partial }}}}_{{\delta }}H(\zeta ) = 0 \quad \text { if }\zeta \not \sim w,w^{*} \\&\quad \text {and } \quad {{{\bar{\partial }}}}_{{\delta }}H(\zeta ) = \frac{\pi }{2} \, {\mathbb {E}}\! \left[ { \prod _{x\in W}{\sigma }_{x} } \right] \qquad \text { if }\zeta \sim w. \end{aligned}$$

##### Proof

See Sect. 6.2. $$\square $$

We will mostly use the above result in the following form:

##### Corollary 3.23

Let $W \subset V \subset {{\mathbb {C}}_{{\delta }}}$, $w \in {\left[ {{\Omega }}_{{\delta }}^{{\mathfrak {m}}}, V \right] }$, and $H :{\left[ {{\Omega }}_{{\delta }}^{{\mathfrak {m}}}, V \right] } \rightarrow {\mathbb {C}}$ be as above. If $\gamma$ is a contractible closed contour on the double cover ${\left[ {{\Omega }}_{{\delta }}^{{\mathfrak {c}}}, V \right] }$ such that $\mathrm {Int}[{\gamma }] \supset \left\{ w \pm \frac{{\delta }}{2},w\pm \frac{\mathbb {i}{\delta }}{2} \right\}$ and such that $\mathrm {Int}[{\gamma }] \cap V = \emptyset$ and $f :{\left[ {{\Omega }}_{{\delta }}^{\diamond }, V \right] } \rightarrow {\mathbb {C}}$ is discrete holomorphic on $\mathrm {Int}[{\gamma }]$, then

##### Proof

Using the discrete Stokes’ formula (2.1), the contractible contour $\gamma$ can be deformed to the trivial contour plaquette by plaquette, and only the plaquettes with non-zero ${{{\bar{\partial }}}}_{{\delta }}H$ contribute to the contour integral. Proposition 3.22 states that these plaquettes are exactly the $\zeta \sim w$ and gives the values ${{{\bar{\partial }}}}_{{\delta }}H(\zeta )$. $\square$

##### Remark 3.24

We can say informally that $z \mapsto {\psi }(w) {\psi }(z)$ has four discrete poles of residue $\frac{1}{4}$ at the four diamond vertices next to *w*, as long as we avoid $\partial {{\Omega }}_{{\delta }}$, *V* and $w^{*}$.

## Virasoro Algebra at the Lattice Level

In this section, we implement at the lattice level the Virasoro mode operators ${\mathsf {L}}_{n}$, $n \in {\mathbb {Z}}$, for the discrete Gaussian free field and the Ising model. The idea is to consider the relevant Laurent modes of the discrete holomorphic current and fermion, respectively, to obtain commutation relations and to construct the Virasoro modes from them. At all stages of the construction we need to make sure that we indeed obtain lattice local fields (modulo lattice null fields) and hence work on probabilistic objects rather than just on an abstract notion of fields. It is quite remarkable that this is possible at all, given how rigid the theory of discrete complex analysis is (in particular, the fact that the product of two discrete holomorphic functions is not discrete holomorphic in general), and given how simple our definition of lattice local field is.

Throughout this section, discrete contour integration is performed over (closed counterclockwise) discrete contours $\gamma$ on the corner lattice, as in Sect. 2, and we denote by $\mathrm {Int}[{\gamma }]$ the set of points surrounded by $\gamma$. For a lattice local field ${{\mathcal {O}}}$, we will denote by ${{\mathcal {D}}}_{{{\mathcal {O}}}}$ the smallest disk $D\left( 0,\rho \right)$ centered at the origin such that ${{\mathcal {O}}}(0)$ does not depend on field values outside of $D\left( 0,\rho \right)$. For $k\in {\mathbb {Z}}\cup \left( {\mathbb {Z}}+\frac{1}{2}\right)$, we will denote by $D_{k}$ the smallest disk $D\left( 0,\rho \right)$ such that ${z^{[{k}]}}$ is discrete holomorphic outside of $D\left( 0,\rho \right)$.

### Gaussian free field

We first address the case of the discrete Gaussian free field. Let us denote by ${\mathfrak {F}}^\mathrm {loc}_{{\mathcal {G}}}$ the space of dGFF local fields and ${\mathfrak {F}}^\mathrm {null}_{{\mathcal {G}}}$ the subspace of null fields, as in Definitions 3.1 and 3.3. Our construction of the Laurent mode operators will take place in the space

$$\begin{aligned} {\mathfrak {F}}_{{\mathcal {G}}}= {\mathfrak {F}}^\mathrm {loc}_{{\mathcal {G}}}/ {\mathfrak {F}}^\mathrm {null}_{{\mathcal {G}}}\end{aligned}$$

of correlation equivalence classes of local fields of dGFF.

#### Current modes

**Fig. 8:**
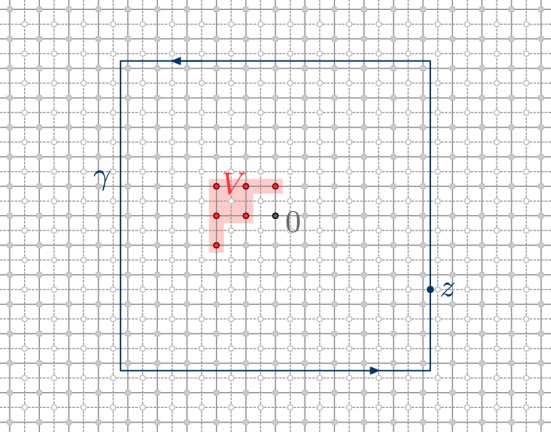
The action of current modes on local fields is by contour integration

Let us recall that we denote by ${J}(z) = \mathbb {i}\, \partial {\varphi }(z)$ the discrete holomorphic current. Below we define its discrete Laurent modes ${\mathsf {J}}_{{k}}$, $k \in {\mathbb {Z}}$, see Fig. 8 for an illustration.

##### Definition 4.1

Let ${{\mathcal {O}}}\in {\mathfrak {F}}^\mathrm {loc}_{{\mathcal {G}}}$ be a lattice local field and let $k\in {\mathbb {Z}}$. Let $\gamma$ be such that $\mathrm {Int}[{\gamma }]\supset {{\mathcal {D}}}_{{{\mathcal {O}}}} \cup D_k$. To define a new local field ${\mathsf {J}}_{{k}}^{\gamma } {{\mathcal {O}}}\in {\mathfrak {F}}^\mathrm {loc}_{{\mathcal {G}}}$, it is enough to specify its value ${\mathsf {J}}_{{k}}^{\gamma }{{\mathcal {O}}}(0)$ at the origin, since local fields are given by a translation invariant rule. We define ${\mathsf {J}}_{{k}}^{\gamma }{{\mathcal {O}}}(0)$ by

##### Lemma 4.2

The following properties hold for ${\mathsf {J}}_{{k}}^{\gamma }{{\mathcal {O}}}$:

- If  ${{\mathcal {O}}}\in {\mathfrak {F}}^\mathrm {loc}_{{\mathcal {G}}}$ is a local field and $\gamma ,{\tilde{\gamma }}$ are two large enough closed counterclockwise contours, then we have ${ {\mathsf {J}}_{{k}}^{\gamma }{{\mathcal {O}}}- {\mathsf {J}}_{{k}}^{{\tilde{\gamma }}}{{\mathcal {O}}}\in {\mathfrak {F}}^\mathrm {null}_{{\mathcal {G}}}}$.
- If ${{\mathcal {O}}}, {\widetilde{{{\mathcal {O}}}}} \in {\mathfrak {F}}^\mathrm {loc}_{{\mathcal {G}}}$ are two local fields such that ${{\mathcal {O}}}- {\widetilde{{{\mathcal {O}}}}} \in {\mathfrak {F}}^\mathrm {null}_{{\mathcal {G}}}$ and $\gamma$ is a large enough closed counterclockwise contour, then we have ${\mathsf {J}}_{{k}}^{\gamma }{{\mathcal {O}}}- {\mathsf {J}}_{{k}}^{\gamma }{\widetilde{{{\mathcal {O}}}}} \in {\mathfrak {F}}^\mathrm {null}_{{\mathcal {G}}}$.

##### Proof

Consider two different contours $\gamma$ and ${\tilde{\gamma }}$. It follows from discrete holomorphicity of ${z_{\diamond }^{[{k}]}}$ (Proposition 2.1(3)), discrete holomorphicity of correlations of ${J}(z_{{\mathfrak {m}}})$ (Lemma 3.5), and the contour deformation property (2.2), that the correlations of ${\mathsf {J}}_{{k}}^{\gamma } {{\mathcal {O}}}(0)$ and ${\mathsf {J}}_{{k}}^{{\tilde{\gamma }}} {{\mathcal {O}}}(0)$ with other insertions at large enough distance are equal. In other words, ${\mathsf {J}}_{{k}}^{{\tilde{\gamma }}} {{\mathcal {O}}}- {\mathsf {J}}_{{k}}^{\gamma } {{\mathcal {O}}}$ is null. This proves (a).

Consider then two different local fields ${{\mathcal {O}}}$ and ${\widetilde{{{\mathcal {O}}}}}$ such that ${{\mathcal {O}}}- {\widetilde{{{\mathcal {O}}}}}$ is null. Provided that the contour $\gamma$ is sufficiently large, then for any $z_{{\mathfrak {m}}}$ on $\gamma$, the correlations of $\big ( {{\mathcal {O}}}(0) - {\widetilde{{{\mathcal {O}}}}}(0) \big ) {J}(z_{{\mathfrak {m}}})$ with other insertions sufficiently far away are vanishing. Therefore, by linearity, the correlations of ${\mathsf {J}}_{{k}}^{\gamma } {{\mathcal {O}}}(0) - {\mathsf {J}}_{{k}}^{\gamma } {\widetilde{{{\mathcal {O}}}}}(0)$ with other insertions are also vanishing, so indeed ${\mathsf {J}}_{{k}}^{\gamma } {{\mathcal {O}}}- {\mathsf {J}}_{{k}}^{\gamma } {\widetilde{{{\mathcal {O}}}}}$ is null. This proves (b). $\square$

This lemma hence allows one to define the current mode action on the space ${\mathfrak {F}}_{{\mathcal {G}}}$ of dGFF local fields modulo null fields.

##### Definition 4.3

For a lattice local field ${{\mathcal {O}}}\in {\mathfrak {F}}^\mathrm {loc}_{{\mathcal {G}}}$ and $k \in {\mathbb {Z}}$, we define ${\mathsf {J}}_{{k}}{{\mathcal {O}}}\in {\mathfrak {F}}_{{\mathcal {G}}}$ as the correlation equivalence class

$$\begin{aligned} {\mathsf {J}}_{{k}}{{\mathcal {O}}}:= {\mathsf {J}}_{{k}}^{\gamma } {{\mathcal {O}}}+ {\mathfrak {F}}^\mathrm {null}_{{\mathcal {G}}}, \end{aligned}$$

which is independent of the choice of a large enough $\gamma$ by Lemma 4.2(b). Moreover, by Lemma 4.2(a) we have that the ${\mathsf {J}}_{{k}}{{\mathcal {O}}}$ only depends on the correlation equivalence class of ${{\mathcal {O}}}$, so this defines operators

$$\begin{aligned} {\mathsf {J}}_{{k}} :{\mathfrak {F}}_{{\mathcal {G}}}\rightarrow {\mathfrak {F}}_{{\mathcal {G}}}. \end{aligned}$$

The following annihilation property for the current modes will be important to define the Virasoro modes below.

##### Lemma 4.4

Let ${{\mathcal {O}}}$ be a lattice local field. There exists $K>0$ such that for any $k\ge K$, ${\mathsf {J}}_{{k}}{{\mathcal {O}}}=0$ modulo ${\mathfrak {F}}^\mathrm {null}_{{\mathcal {G}}}$.

##### Proof

With a large but fixed contour $\gamma$, by Item 7 of Proposition 2.1 we can choose *K* such that for all $z_{\diamond }$ on the contour and $k \ge K$ we have ${z_{\diamond }^{[{k}]}} = 0$. It then follows directly from the definition that ${\mathsf {J}}_{{k}}^{\gamma } {{\mathcal {O}}}= 0$. $\square$

##### Proposition 4.5

The operators $\left( {\mathsf {J}}_{{k}}\right) _{k\in {\mathbb {Z}}}$ form a representation of the Heisenberg algebra on ${\mathfrak {F}}_{{\mathcal {G}}}$, i.e., for all $k,l \in {\mathbb {Z}}$ we have the commutation relation

$$\begin{aligned} \left[ {\mathsf {J}}_{{k}}, {\mathsf {J}}_{{\ell }} \right] = \; k \delta _{k,-\ell } \; \, \mathsf {id}_{{\mathfrak {F}}_{{\mathcal {G}}}} . \end{aligned}$$

##### Proof

**Fig. 9:**
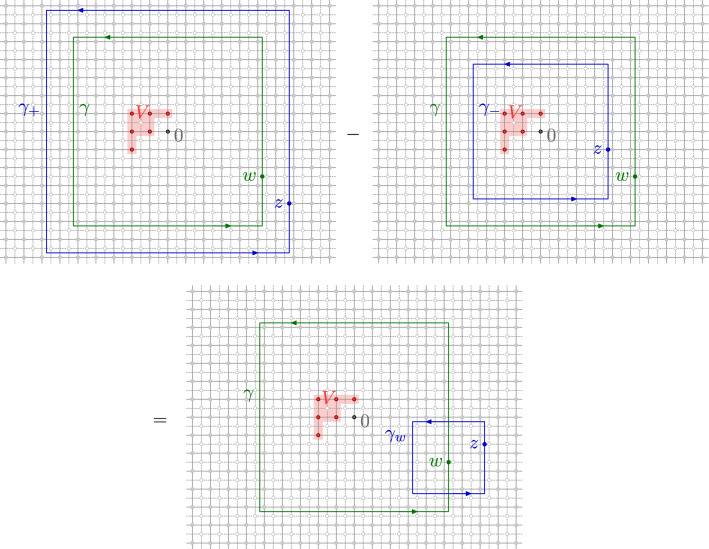
The commutator $\left[ {\mathsf {J}}_{{k}}, {\mathsf {J}}_{{\ell }}\right]$ is expressed as the difference of two integrals where the order of the *z* and *w* contours are exchanged, yielding a ‘satellite’ contour integral over *z* ‘orbiting’ around *w*, which itself orbits around 0 and the finite set *V* of points used to define the local field ${{\mathcal {O}}}$ on which the modes are acting

Let ${{\mathcal {O}}}$ be a lattice local field and let $\gamma ,{\gamma }_-,{\gamma }_+$ be sufficiently large closed counterclockwise contours nested around each other so that $\mathrm {Int}[{{\gamma }_-}] \subset \mathrm {Int}[{\gamma }] \subset \mathrm {Int}[{{\gamma }_+}]$. The local field ${ {\mathsf {J}}_{{k}}^{{\gamma }_+} {\mathsf {J}}_{{\ell }}^{\gamma }{{\mathcal {O}}}- {\mathsf {J}}_{{\ell }}^{\gamma } {\mathsf {J}}_{{k}}^{{\gamma }_-}{{\mathcal {O}}}}$ is by definition a difference of two double contour integrals. The discrete holomorphicity of the current ${J}$ (Lemma 3.5) allows us to deform these contours within correlations. More precisely, if $V \subset {{\mathbb {C}}_{{\delta }}}$ is a finite set located far enough outside the outermost contour ${\gamma }_+$ and $F({\varphi }|_V)$ is a polynomial of the values of the DGFF in *V*, then we can writewhere the *w*-integration is kept intact while for each fixed *w* the difference of the *z*-integrals have been combined and deformed to a “satellite integral” along a part $\gamma _w$ encircling *w* clockwise (see Fig. 9). We then apply Lemma 3.6 to evaluate the inner satellite integral, using also properties of discrete monomials (Proposition 2.1), and we getThis equality shows that up to null fields we have

$$\begin{aligned} {\mathsf {J}}_{{k}}^{{\gamma }_+} {\mathsf {J}}_{{\ell }}^{\gamma } {{\mathcal {O}}}(0) - {\mathsf {J}}_{{\ell }}^{\gamma } {\mathsf {J}}_{{k}}^{{\gamma }_-} {{\mathcal {O}}}(0) \, \equiv \; k \delta _{k,-\ell } \; {{\mathcal {O}}}(0) , \end{aligned}$$

which proves the asserted commutation relation of ${\mathsf {J}}_{{k}}$ and ${\mathsf {J}}_{{\ell }}$. $\square$

#### Gaussian free field Virasoro modes

##### Definition 4.6

We define the operator ${\mathsf {L}}_{n}^{{\mathcal {G}}} :{\mathfrak {F}}_{{\mathcal {G}}}\rightarrow {\mathfrak {F}}_{{\mathcal {G}}}$ by

$$\begin{aligned} {\mathsf {L}}_{n}^{{\mathcal {G}}} = \frac{1}{2} \sum _{k\in {\mathbb {Z}}} {(\overset{\underleftrightarrow { k }}{{{{{\mathsf {J}}_{{n-k}}}}{{{\mathsf {J}}_{{k}}}}}}}) \end{aligned}$$

where we set

$$\begin{aligned} {(\overset{\underleftrightarrow { k }}{{{{A}}{{B}}}}}) = {\left\{ \begin{array}{ll} A B &{} \quad \text { if }k \ge 0 \\ B A &{} \quad \text { if }k < 0 . \end{array}\right. } \end{aligned}$$

##### Remark 4.7

By virtue of Lemma 4.4, the sum above is well-defined as an operator on the space ${\mathfrak {F}}_{{\mathcal {G}}}$ of lattice local fields: there are only finitely many non-null terms, when the sum acts on (the correlation equivalence class of) any given lattice local field.

##### Remark 4.8

Our choice of the above definition is guided by the convenience of the calculations below, but it can also be easily seen to agree with the common definition ${\mathsf {L}}_{n}^{{\mathcal {G}}} = {\frac{1}{2}\sum _{k\in {\mathbb {Z}}}: {\mathsf {J}}_{{n-k}} {\mathsf {J}}_{{k}}:}$ where the normal-ordered product ${: {\mathsf {J}}_{{j}} {\mathsf {J}}_{{k}}:}$ is defined as ${\mathsf {J}}_{{j}} {\mathsf {J}}_{{k}}$ if $j\le k$ and as ${\mathsf {J}}_{{k}} {\mathsf {J}}_{{j}}$ otherwise.

##### Lemma 4.9

For any $n,m,k \in {\mathbb {Z}}$, we have

$$\begin{aligned} \big [{\mathsf {L}}_{n}^{{\mathcal {G}}}, {\mathsf {J}}_{{m}} \big ]&= - m \, {\mathsf {J}}_{{n+m}} \\ \big [ {\mathsf {L}}_{n}^{{\mathcal {G}}}, {(\overset{\underleftrightarrow { k }}{{{{{\mathsf {J}}_{{m-k}}}}{{{\mathsf {J}}_{{k}}}}}}}) \big ]&= - k {(\overset{\underleftrightarrow { k }}{{{{{\mathsf {J}}_{{m-k}}}}{{{\mathsf {J}}_{{n+k}}}}}}}) - \left( m-k\right) {(\overset{\underleftrightarrow { k }}{{{{{\mathsf {J}}_{{n+m-k}}}}{{{\mathsf {J}}_{{k}}}}}}}) . \end{aligned}$$

##### Proof

The second formula follows from the first by using the commutator identity $[A,BC] = B[A,C] + [A,B]C$, so it remains to prove the first formula. Observe first that by the commutator identity $[AB,C] = A[B,C] + [A,C]B$ and Proposition 4.5 we have, when $k + \ell = n$,

$$\begin{aligned}{}[{\mathsf {J}}_{{\ell }} {\mathsf {J}}_{{k}} , {\mathsf {J}}_{{m}}]&= {\mathsf {J}}_{{\ell }} [ {\mathsf {J}}_{{k}} , {\mathsf {J}}_{{m}} ] + [{\mathsf {J}}_{{\ell }} , {\mathsf {J}}_{{m}} ] {\mathsf {J}}_{{k}} \\&= {\mathsf {J}}_{{\ell }} \; k \, \delta _{k+m,0} + \ell \, \delta _{\ell +m,0} \; {\mathsf {J}}_{{k}} = -m \, (\delta _{k,-m} + \delta _{k,n+m}) \, {\mathsf {J}}_{{n+m}} . \end{aligned}$$

Using this, we calculate

$$\begin{aligned} \big [ {\mathsf {L}}_{n}^{{\mathcal {G}}}, {\mathsf {J}}_{{m}} \big ]&= \frac{1}{2} \; \sum _{k \in {\mathbb {Z}}} \big [ {(\overset{\underleftrightarrow { k }}{{{{{\mathsf {J}}_{{n-k}}}}{{{\mathsf {J}}_{{k}}}}}}}) , {\mathsf {J}}_{{m}} \big ] \\&= \frac{1}{2} \; \sum _{k \in {\mathbb {Z}}} \Big ( -m \, (\delta _{k,-m} + \delta _{k,n+m}) \, {\mathsf {J}}_{{n+m}} \Big ) = -m \, {\mathsf {J}}_{{n+m}} , \end{aligned}$$

where by virtue of Proposition 4.4, the sums over *k* again have only finitely many non-zero terms when acting on a given correlation equivalence class of local fields. $\square$

##### Theorem 4.10

The operators $\left( {\mathsf {L}}_{n}^{G}\right) _{n\in {\mathbb {Z}}}$ form a representation of the Virasoro algebra with central charge ${c=1}$, namely

$$\begin{aligned} \left[ {\mathsf {L}}_{n}^{{\mathcal {G}}},{\mathsf {L}}_{m}^{{\mathcal {G}}}\right] = \left( n-m\right) {\mathsf {L}}_{n+m}^{{\mathcal {G}}} + \frac{1}{12} \delta _{n+m,0} \left( n^{3}-n\right) \mathsf {id}_{{\mathfrak {F}}_{{\mathcal {G}}}} . \end{aligned}$$

##### Proof

Let us omit the ${\mathcal {G}}$ superscript. To compute $\left[ {\mathsf {L}}_{n},{\mathsf {L}}_{m}\right]$, write ${\mathsf {L}}_{m} = \frac{1}{2}\sum _{k\in {\mathbb {Z}}} {(\overset{\underleftrightarrow { k }}{{{{{\mathsf {J}}_{{m-k}}}}{{{\mathsf {J}}_{{k}}}}}}})$, use the second formula of Lemma 4.9, and perform a change of variables $\ell = k+n$ in the first part to obtain that

$$\begin{aligned} \left[ {\mathsf {L}}_{n},{\mathsf {L}}_{m}\right]&= \frac{1}{2} \sum _{k\in {\mathbb {Z}}} \big [ {\mathsf {L}}_{n}, {(\overset{\underleftrightarrow { k }}{{{{{\mathsf {J}}_{{m-k}}}}{{{\mathsf {J}}_{{k}}}}}}}) \big ] = \frac{1}{2} \sum _{k\in {\mathbb {Z}}} \Big ( - k {(\overset{\underleftrightarrow { k }}{{{{{\mathsf {J}}_{{m-k}}}}{{{\mathsf {J}}_{{n+k}}}}}}}) - (m-k) {(\overset{\underleftrightarrow { k }}{{{{{\mathsf {J}}_{{n+m-k}}}}{{{\mathsf {J}}_{{k}}}}}}}) \Big ) \\&= \frac{1}{2} \sum _{\ell \in {\mathbb {Z}}} (n-\ell ) {(\overset{\underleftrightarrow { \ell -n }}{{{{{\mathsf {J}}_{{n+m-\ell }}}}{{{\mathsf {J}}_{{\ell }}}}}}}) + \frac{1}{2} \sum _{k\in {\mathbb {Z}}} (k-m) {(\overset{\underleftrightarrow { k }}{{{{{\mathsf {J}}_{{n+m-k}}}}{{{\mathsf {J}}_{{k}}}}}}}) \\&= (n-m) \, {\mathsf {L}}_{n+m} + {\mathcal {A}}_{n,m} , \end{aligned}$$

where

$$\begin{aligned} {\mathcal {A}}_{n,m} := \frac{1}{2}\sum _{\ell \in {\mathbb {Z}}} (n-\ell ) \Big ( {(\overset{\underleftrightarrow { \ell -n }}{{{{{\mathsf {J}}_{{n+m-\ell }}}}{{{\mathsf {J}}_{{\ell }}}}}}}) - {(\overset{\underleftrightarrow { \ell }}{{{{{\mathsf {J}}_{{n+m-\ell }}}}{{{\mathsf {J}}_{{\ell }}}}}}}) \Big ) . \end{aligned}$$

Now note that by Proposition 4.5 we get

$$\begin{aligned} {(\overset{\underleftrightarrow { \ell -n }}{{{{{\mathsf {J}}_{{n+m-\ell }}}}{{{\mathsf {J}}_{{\ell }}}}}}}) - {(\overset{\underleftrightarrow { \ell }}{{{{{\mathsf {J}}_{{n+m-\ell }}}}{{{\mathsf {J}}_{{\ell }}}}}}}) = \;&{\left\{ \begin{array}{ll} \ell \, \delta _{n+m,0} &{} \quad \text {if } 0 \le \ell< n \\ -\ell \, \delta _{n+m,0} &{} \quad \text {if } n \le \ell < 0 \\ 0 &{} \quad \text {otherwise} . \end{array}\right. } \end{aligned}$$

Observing furthermore that $\sum _{\ell =0}^{n} (n-\ell ) \ell = \frac{1}{6}(n^3-n)$ for $n\ge 0$, we can therefore simplify

$$\begin{aligned} {\mathcal {A}}_{n,m}&= \frac{1}{12} \, (n^3-n) \, \delta _{n+m,0}, \end{aligned}$$

and conclude the proof. $\square$

### Ising model

The Ising model involves a number of additional difficulties. First of all, since the fermion field is not local, its modes cannot act on local fields, and we must hence consider modes of fermion pairs. Second, since the fermion is quasi-local with respect to spin-antisymmetric fields, we must take half-integer power Laurent modes of it (while taking integer modes of it when acting on spin-symmetric fields), see Definition 4.14. Third, since the fermion pairs involve defect lines, one must be careful while choosing them and exchanging them to get the right commutation modes.

Recall that we denote by ${\mathfrak {F}}^\mathrm {loc}_{{\mathcal {I}}}$ the space of Ising local fields, ${\mathfrak {F}}^\mathrm {null}_{{\mathcal {I}}}$ the subspace of null fields, and by

$$\begin{aligned} {\mathfrak {F}}_{{\mathcal {I}}}= {\mathfrak {F}}^\mathrm {loc}_{{\mathcal {I}}}/ {\mathfrak {F}}^\mathrm {null}_{{\mathcal {I}}}\end{aligned}$$

the space of correlation equivalence classes of local fields of the Ising model. Note that the space of local fields splits to a direct sum ${\mathfrak {F}}^\mathrm {loc}_{{\mathcal {I}}}= {\mathfrak {F}}^\mathrm {loc}_{{\mathcal {I}}; \mathrm {even}}\oplus {\mathfrak {F}}^\mathrm {loc}_{{\mathcal {I}}; \mathrm {odd}}$ of spin-symmetric fields (fields ${{\mathcal {O}}}_{{\delta }}$ which are even under global spin flip ${\sigma }\mapsto -{\sigma }$, i.e., ${{\mathcal {O}}}_{{\delta }} [-{\sigma }] = {{\mathcal {O}}}_{{\delta }}[{\sigma }]$) and spin-antisymmetric fields (fields ${{\mathcal {O}}}_{{\delta }}$ which are odd under global spin flip, i.e., ${{\mathcal {O}}}_{{\delta }} [-{\sigma }] = - {{\mathcal {O}}}_{{\delta }}[{\sigma }]$). All considerations below will be done separately for these two sectors.

#### Fermion modes

**Fig. 10:**
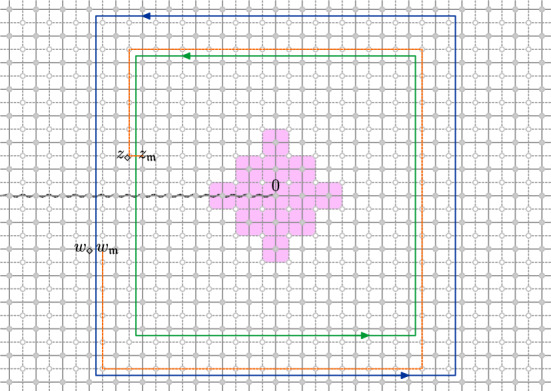
The setup for the integrand of the definition of $\left( {\Psi }_{p}^{\gamma }{\Psi }_{q}^{\alpha }{{\mathcal {O}}}\right) _{ {\lambda }}.$ The set *E* is the shaded area. The contours with arrows are the integration contours $\alpha$ (inner, green) and $\gamma$ (outer, blue) respectively. The marked medial vertices are the $z_{{\mathfrak {m}}}$ and $w_{{\mathfrak {m}}}$ that appear in the integral (respectively on the inner contour and on the outer contour), and on which the fermion ${\psi }$ is defined. The marked diamond vertices are the $z_{\diamond }$ and $w_{\diamond }$ (on the inner and outer contours) on which the monomials are defined. An example of two pairs $z_{{\mathfrak {m}}},z_{\diamond }$ and $w_{{\mathfrak {m}}},w_{\diamond }$ is drawn here. The main part of the corresponding medial defect line is drawn in dashed orange stroke. The defect lines are chosen not to cross the branch cut on ${\mathbb {R}}_{-}$, marked by the wiggly line

We now define fermion Laurent mode pairs in the even and odd sector, separately. In the even sector we use integer powers, but half-integer indices, and in the odd sector we use half-integer powers but integer indices—this convention of indexing is due to half-integer scaling dimension of the fermion field. As before, since lattice local fields are translation invariant in their definition, to define a lattice local field, we only need to give the definition at the origin (Fig. 10).

##### Definition 4.11

Let ${{\mathcal {O}}}\in {\mathfrak {F}}^\mathrm {loc}_{{\mathcal {I}}; \mathrm {even}}$ be a spin-symmetric lattice local field. Let

$$\begin{aligned} p,q\in {\mathbb {Z}}+\frac{1}{2} . \end{aligned}$$

Set $E := D_{p}\cup D_{q}\cup {{\mathcal {D}}}_{{{\mathcal {O}}}}$. Let $\gamma , \alpha$ be counterclockwise closed paths such that $\mathrm {Int}[{\gamma }] \supset \mathrm {Int}[{\alpha }] \supset E$ and let ${\lambda }$ be a choice of medial defect lines that does not cross ${\mathbb {R}}_{-}\cup E$ for all $z,w \in {{\mathbb {C}}_{{\delta }}^{{\mathfrak {m}}}}\setminus E$. We define

##### Definition 4.12

Let ${{\mathcal {O}}}\in {\mathfrak {F}}^\mathrm {loc}_{{\mathcal {I}}; \mathrm {odd}}$ be a spin-antisymmetric lattice local field. Let

$$\begin{aligned} k , \ell \in {\mathbb {Z}}. \end{aligned}$$

Set $E := D_{k} \cup D_{\ell } \cup {{\mathcal {D}}}_{{{\mathcal {O}}}}$. Let $\gamma ,\alpha$ be counterclockwise closed paths such that $\mathrm {Int}[{\gamma }] \supset \mathrm {Int}[{\gamma }] \supset E$ and let ${\lambda }$ be a choice of medial defect lines that does not cross ${\mathbb {R}}_{-} \cup E$ for all $z,w \in {{\mathbb {C}}_{{\delta }}^{{\mathfrak {m}}}}\setminus E$. We definewhere the branches of ${z^{[{k-\frac{1}{2}}]}}$ and ${w^{[{\ell -\frac{1}{2}}]}}$ are the principal branches on ${\mathbb {C}}\setminus {\mathbb {R}}_{-}$ (i.e. the real part is nonnegative on the positive real axis).

The following lemma tells us that the choices of $\alpha$, $\gamma$ and ${\lambda }$ are essentially irrelevant, modulo null fields. Similarly the choice of the branch cut on ${\mathbb {R}}_{-}$ is irrelevant as well.

##### Lemma 4.13

The following properties hold for $\left( {\Psi }_{p}^{\gamma }{\Psi }_{q}^{\alpha } {{\mathcal {O}}}\right) _{{\lambda }}$:

- If ${{\mathcal {O}}}\in {\mathfrak {F}}^\mathrm {loc}_{{\mathcal {I}}; \mathrm {even}}$ is a spin-symmetric local field, and ${\lambda }$ and ${\widetilde{{\lambda }}}$ are two choices of the defect lines as above, then we have for $p,q \in {\mathbb {Z}}+ \frac{1}{2}$
  $$\begin{aligned} \big ( {\Psi }^{\gamma }_{p} {\Psi }^{\alpha }_{q} {{\mathcal {O}}}\big )_{{\lambda }} - \big ( {\Psi }^{\gamma }_{p} {\Psi }^{\alpha }_{q} {{\mathcal {O}}}\big )_{{\widetilde{{\lambda }}}} \in {\mathfrak {F}}^\mathrm {null}_{{\mathcal {I}}; \mathrm {even}}. \end{aligned}$$
  The analogous property also holds for $\big ( {\Psi }^{\gamma }_{k} {\Psi }^{\alpha }_{\ell } {{\mathcal {O}}}\big )_{{\lambda }}$ for any spin-antisymmetric field ${{{\mathcal {O}}}\in {\mathfrak {F}}^\mathrm {loc}_{{\mathcal {I}}; \mathrm {odd}}}$ and $k,\ell \in {\mathbb {Z}}$.
- If ${{\mathcal {O}}}\in {\mathfrak {F}}^\mathrm {loc}_{{\mathcal {I}}; \mathrm {even}}$ is a spin-symmetric local field and $\gamma ,\alpha$ and ${\tilde{\gamma }},{{\tilde{\alpha }}}$ are two pairs of large enough closed counterclockwise contours, then for any defect lines ${\widetilde{{\lambda }}}$ chosen for the contours ${\tilde{\gamma }},{{\tilde{\alpha }}}$, we have
  $$\begin{aligned} \big ( {\Psi }^{\gamma }_{p} {\Psi }^{\alpha }_{q} {{\mathcal {O}}}\big )_{{\lambda }} - \big ( {\Psi }^{{\tilde{\gamma }}}_{p} {\Psi }^{{{\tilde{\alpha }}}}_{q} {{\mathcal {O}}}\big )_{{\widetilde{{\lambda }}}} \in {\mathfrak {F}}^\mathrm {null}_{{\mathcal {I}}; \mathrm {even}}. \end{aligned}$$
  The analogous property also holds for $\big ( {\Psi }^{\gamma }_{k} {\Psi }^{\alpha }_{\ell } {{\mathcal {O}}}\big )_{{\lambda }}$ where ${{{\mathcal {O}}}\in {\mathfrak {F}}^\mathrm {loc}_{{\mathcal {I}}; \mathrm {odd}}}$ is any spin-antisymmetric field and $k,\ell \in {\mathbb {Z}}$.
- If ${{\mathcal {O}}}, {\widetilde{{{\mathcal {O}}}}} \in {\mathfrak {F}}^\mathrm {loc}_{{\mathcal {I}}; \mathrm {even}}$ are two spin-symmetric local fields such that ${{\mathcal {O}}}- {\widetilde{{{\mathcal {O}}}}} \in {\mathfrak {F}}^\mathrm {null}_{{\mathcal {I}}; \mathrm {even}}$ and $\gamma , \alpha$ are two large enough closed counterclockwise contours, then we have for $p,q \in {\mathbb {Z}}+ \frac{1}{2}$
  $$\begin{aligned} \big ( {\Psi }^{\gamma }_{p} {\Psi }^{\alpha }_{q} {{\mathcal {O}}}\big )_{{\lambda }} - \big ( {\Psi }^{\gamma }_{p} {\Psi }^{\alpha }_{q} {\widetilde{{{\mathcal {O}}}}} \big )_{{\lambda }} \in {\mathfrak {F}}^\mathrm {null}_{{\mathcal {I}}; \mathrm {even}}. \end{aligned}$$
  The analogous property also holds for $\big ( {\Psi }^{\gamma }_{k} {\Psi }^{\alpha }_{\ell } {{\mathcal {O}}}\big )_{{\lambda }}$ where ${{{\mathcal {O}}}, {\widetilde{{{\mathcal {O}}}}} \in {\mathfrak {F}}^\mathrm {loc}_{{\mathcal {I}}; \mathrm {odd}}}$ are any spin-antisymmetric fields and $k,\ell \in {\mathbb {Z}}$.

##### Proof

To prove statements modulo null fields, one argues within correlations as in Lemma 4.2. The independence on the choice of defect lines follows from Lemma 3.14. The rest of the proof is similar to that of Lemma 4.2. Part (b) relies on the discrete holomorphicity of the correlations involving two fermion insertions (Proposition 3.22) and and the discrete holomorphicity of the discrete monomials (Propositions 2.1 and 2.3). For the spin-symmetric case, one furthermore needs to observe the single-valuedness of the fermion around spin-symmetric fields Proposition 3.17, whereas for the spin-antisymmetric case one uses Lemma 2.2, observing that both the half-integer monomial and the fermion have monodromy $-1$ around the set *E*. $\square$

This lemma again allows one to define the fermion mode pair action on the spaces ${\mathfrak {F}}_{{\mathcal {I}}; \mathrm {even}}$ and ${\mathfrak {F}}_{{\mathcal {I}}; \mathrm {odd}}$ of Ising local fields modulo null fields.

##### Definition 4.14

For a spin-symmetric lattice local field ${{\mathcal {O}}}\in {\mathfrak {F}}^\mathrm {loc}_{{\mathcal {I}}; \mathrm {even}}$ and $p,q \in {\mathbb {Z}}+ \frac{1}{2}$, we define ${\Psi }_{p} {\Psi }_{q} \, {{\mathcal {O}}}\in {\mathfrak {F}}_{{\mathcal {I}}; \mathrm {even}}$ as the correlation equivalence class

$$\begin{aligned} {\Psi }_{p} {\Psi }_{q} \, {{\mathcal {O}}}:= \big ( {\Psi }^{\gamma }_{p} {\Psi }^{\alpha }_{q} {{\mathcal {O}}}\big )_{{\lambda }} + {\mathfrak {F}}^\mathrm {null}_{{\mathcal {I}}; \mathrm {even}}, \end{aligned}$$

which is independent of the choice of a large enough contours $\gamma$, $\alpha$ and defect lines ${\lambda }$ by Lemma 4.13(a,b). Moreover, by Lemma 4.13(c) we have that the ${\Psi }_{p} {\Psi }_{q} \, {{\mathcal {O}}}$ only depends on the correlation equivalence class of ${{\mathcal {O}}}$, so this defines operators

$$\begin{aligned} {\Psi }_{p} {\Psi }_{q} \; :\; {\mathfrak {F}}_{{\mathcal {I}}; \mathrm {even}}\rightarrow {\mathfrak {F}}_{{\mathcal {I}}; \mathrm {even}}. \end{aligned}$$

Similarly, for a spin-antisymmetric lattice local field ${{\mathcal {O}}}\in {\mathfrak {F}}^\mathrm {loc}_{{\mathcal {I}}; \mathrm {odd}}$ and $k,\ell \in {\mathbb {Z}}$, we define the correlation equivalence class ${\Psi }_{k} {\Psi }_{\ell } \, {{\mathcal {O}}}\in {\mathfrak {F}}_{{\mathcal {I}}; \mathrm {odd}}$ and obtain operators

$$\begin{aligned} {\Psi }_{k} {\Psi }_{\ell } \; :\; {\mathfrak {F}}_{{\mathcal {I}}; \mathrm {odd}}\rightarrow {\mathfrak {F}}_{{\mathcal {I}}; \mathrm {odd}}. \end{aligned}$$

The following annihilation property for the fermion modes will be important to define the Virasoro modes below.

##### Lemma 4.15

Let ${{\mathcal {O}}}\in {\mathfrak {F}}^\mathrm {loc}_{{\mathcal {I}}; \mathrm {even}}$ (resp. ${{\mathcal {O}}}\in {\mathfrak {F}}^\mathrm {loc}_{{\mathcal {I}}; \mathrm {odd}}$) be a spin-symmetric (resp. spin-antisymmetric) lattice local field. Then there exists $K>0$ such that for any $p,q \in {\mathbb {Z}}+ \frac{1}{2}$ with $q\ge K$ (resp. $k,\ell \in {\mathbb {Z}}$ with $\ell \ge K$), we have ${\Psi }_{p} {\Psi }_{q} {{\mathcal {O}}}= 0 \in {\mathfrak {F}}_{{\mathcal {I}}; \mathrm {even}}$ (resp. ${\Psi }_{k} {\Psi }_{\ell } {{\mathcal {O}}}= 0 \in {\mathfrak {F}}_{{\mathcal {I}}; \mathrm {odd}}$).

##### Proof

With large but fixed contours $\gamma$, $\alpha$, by Item 7 of Proposition 2.1 (respectively Item 7 of Proposition 2.3) we can choose *K* such that for all $w_{\diamond }$ on the contour and $q \ge K$ we have ${w_{\diamond }^{[{q}]}} = 0$. It then follows directly from the definition that $\big ( {\Psi }^{\gamma }_{p} {\Psi }^{\alpha }_{q} {{\mathcal {O}}}\big )_{{\lambda }} = 0$. $\square$

##### Proposition 4.16

For $p,q,k,l\in {\mathbb {Z}}$ or $p,q,k,l\in {\mathbb {Z}}+\frac{1}{2}$, we have the following anticommutation relations:

4.1 $$\begin{aligned} {\Psi }_{p}{\Psi }_{q}+{\Psi }_{q}{\Psi }_{p}&= \delta _{p,-q} \; \mathsf {id}\end{aligned}$$

4.2 $$\begin{aligned} \left( {\Psi }_{k}{\Psi }_{p}\right) \left( {\Psi }_{q}{\Psi }_{\ell }\right) + \left( {\Psi }_{k}{\Psi }_{q}\right) \left( {\Psi }_{p}{\Psi }_{\ell }\right)&= \delta _{p,-q} \; {\Psi }_{k}{\Psi }_{\ell } . \end{aligned}$$

##### Proof

The calculations are to be performed modulo null fields, i.e., it is sufficient to prove equalities within correlations as in Proposition 4.5. We will indicate equalities up to null fields by $\equiv$.

Let ${{\mathcal {O}}}$ be a lattice local field. For an integer or half-integer *n*, set $n_{-} := n-\frac{1}{2}$. Set $E := D\left( p\right) \cup D\left( q\right) \cup D\left( {{\mathcal {O}}}\right)$. Let us first prove the first identity (4.1). For $\mathrm {Int}[{\gamma }]\supset \mathrm {Int}[{\alpha }]\supset \mathrm {Int}[{{{\tilde{\gamma }}}}]\supset E$, we have (omitting the defect lines ${\lambda }:z_{{\mathfrak {m}}}\leftrightarrow w_{{\mathfrak {m}}}$ to lighten the notation),We can now use $\left( {\psi }(w) {\psi }(z) \right) = - \left( {\psi }(z) {\psi }(w) \right)$ to rewrite the above (within correlations) as a satellite integral as abovewhere $\gamma (w)$ is a small contour with $\mathrm {Int}[{\gamma }] \supset \left\{ w \pm {\delta }, w \pm \mathbb {i}{\delta } \right\}$ and $\mathrm {Int}[{\gamma }]\cap E=\emptyset$. Now, by Corollary 3.23, we have (within correlations)By Property 10 of Proposition 2.1 and Property 9 of Proposition 2.3 (see also Lemma 5.17 below), we havewhich yields

$$\begin{aligned} \big ( {\Psi }^{\gamma }_{p} {\Psi }^{\alpha }_{q} + {\Psi }^{\alpha }_{q} {\Psi }^{{\tilde{\gamma }}}_{p} \big ) {{\mathcal {O}}}\equiv \delta _{p,-q} \; {{\mathcal {O}}}. \end{aligned}$$

The proof of the second identity (4.2) is similar: we have that the integrand in the definitions ${\Psi }_{k}{\Psi }_{p}{\Psi }_{q}{\Psi }_{\ell }{{\mathcal {O}}}(0)$ and ${\Psi }_{k}{\Psi }_{q}{\Psi }_{p}{\Psi }_{\ell }{{\mathcal {O}}}(0)$ is

$$\begin{aligned} {{\mathcal {O}}}(0) \left( {\psi }(\zeta _{{\mathfrak {m}}}) {\psi }(z_{{\mathfrak {m}}}) \right) _{ \iota :\zeta \leftrightarrow z} \left( {\psi }(w_{{\mathfrak {m}}}) {\psi }\left( \xi _{{\mathfrak {m}}}\right) \right) _{\kappa :w\leftrightarrow \xi } {\zeta _{\diamond }^{[{k_{-}}]}} {z_{\diamond }^{[{p_{-}}]}} {w_{\diamond }^{[{q_{-}}]}} {\xi _{\diamond }^{[{\ell _{-}}]}} \end{aligned}$$

where $\iota$ and $\kappa$ are chosen to avoid ${{\mathcal {D}}}_{{{\mathcal {O}}}}$ and not to cross the negative real axis.

By Lemma 3.15, we can exchange the medial defect lines

$$\begin{aligned}&\big ( {\psi }(\zeta _{{\mathfrak {m}}}) {\psi }(z_{{\mathfrak {m}}}) \big )_{\iota :\zeta \leftrightarrow z} \big ({\psi }(w_{{\mathfrak {m}}}) {\psi }(\xi _{{\mathfrak {m}}}) \big )_{\kappa :w\leftrightarrow \xi } \\&= \big ( {\psi }(\zeta _{{\mathfrak {m}}}) {\psi }(\xi _{{\mathfrak {m}}}) \big )_{ \eta :\zeta \leftrightarrow \xi } \big ( {\psi }(z_{{\mathfrak {m}}}) {\psi }(w_{{\mathfrak {m}}}) \big )_{\lambda :z\leftrightarrow w}, \end{aligned}$$

by choosing medial defect lines $\eta$ and $\lambda$ that avoid ${{\mathcal {D}}}_{{{\mathcal {O}}}}$ and do not cross the negative real axis.

Now, ${\Psi }_{k} {\Psi }_{p} {\Psi }_{q} {\Psi }_{\ell } + {\Psi }_{k} {\Psi }_{q} {\Psi }_{p} {\Psi }_{\ell }$ can be evaluated summing the two quadruple integrals of

$$\begin{aligned} \left( {\psi }(\zeta _{{\mathfrak {m}}}) {\psi }(\xi _{{\mathfrak {m}}}) \right) _{\eta :\zeta \leftrightarrow \xi } \left( {\psi }(z_{{\mathfrak {m}}}) {\psi }(w_{{\mathfrak {m}}}) \right) _{\lambda :z\leftrightarrow w} {\zeta _{\diamond }^{[{k_{-}}]}} {z_{\diamond }^{[{p_{-}}]}} {w_{\diamond }^{[{q_{-}}]}} {\xi _{\diamond }^{[{\ell _{-}}]}} \end{aligned}$$

with order of the variables *z* and *w* swapped. We obtain that the result of the second and third integrals can be written as the same satellite integral as for the first identity, yielding the same result. What is left is hence easily seen to be equal (up to null fields) to

$$\begin{aligned} \delta _{p,-q} \; {\Psi }_{k}{\Psi }_{\ell }{{\mathcal {O}}}(0) , \end{aligned}$$

thus concluding the proof. $\square$

#### Ising Virasoro modes

For the next definition, let us introduce the notation

$$\begin{aligned} {(\pm \overset{\underleftrightarrow { {k} }}{{{{A}}{{B}}}}}) = {\left\{ \begin{array}{ll} A B &{} \quad \text { if }k \ge 0 \\ - B A &{} \quad \text { if }k < 0 . \end{array}\right. } \end{aligned}$$

##### Definition 4.17

For $n \in {\mathbb {Z}}$, we define ${\mathsf {L}}_{n}^{\mathrm {even}} :{\mathfrak {F}}_{{\mathcal {I}}; \mathrm {even}}\rightarrow {\mathfrak {F}}_{{\mathcal {I}}; \mathrm {even}}$ and ${\mathsf {L}}_{n}^{\mathrm {odd}} :{\mathfrak {F}}_{{\mathcal {I}}; \mathrm {odd}}\rightarrow {\mathfrak {F}}_{{\mathcal {I}}; \mathrm {odd}}$ by

4.3 $$\begin{aligned} {\mathsf {L}}_{n}^{\mathrm {even}}&:= \frac{1}{2} \sum _{k\in {\mathbb {Z}}+\frac{1}{2}} k \, {(\pm \overset{\underleftrightarrow { {k} }}{{{{{\Psi }_{n-k}}}{{{\Psi }_{k}}}}}}) \end{aligned}$$

4.4 $$\begin{aligned} {\mathsf {L}}_{n}^{\mathrm {odd}}&:= \frac{1}{2} \sum _{k\in {\mathbb {Z}}} k \, {(\pm \overset{\underleftrightarrow { {k} }}{{{{{\Psi }_{n-k}}}{{{\Psi }_{k}}}}}}) + \frac{1}{16} \, \delta _{n,0} \; \mathsf {id}. \end{aligned}$$

##### Remark 4.18

By virtue of Lemma 4.15, the sums above are well-defined as operators on the spaces ${\mathfrak {F}}_{{\mathcal {I}}; \mathrm {even}}$ and ${\mathfrak {F}}_{{\mathcal {I}}; \mathrm {odd}}$ of lattice local fields: there are only finitely many non-null terms, when the sums act on (the correlation equivalence class of) any given lattice local field.

##### Remark 4.19

Using Lemma 4.20 below, the above definitions can easily be checked to equivalently give the following commonly used formulas involving normal orderings

$$\begin{aligned} {\mathsf {L}}_{n}^{\mathrm {even}}&={\left\{ \begin{array}{ll} \frac{1}{2}\sum _{k\in {\mathbb {Z}}+\frac{1}{2}} \left( k+\frac{1}{2}\right) :{\Psi }_{n-k }{\Psi }_{k}: &{}\quad \text {if }n\ne 0\\ \sum _{k=1}^{\infty }k{\Psi }_{-k}{\Psi }_{k} &{}\quad \text {if }n=0 \end{array}\right. }\\ {\mathsf {L}}_{n}^{\mathrm {odd}}&= {\left\{ \begin{array}{ll} \frac{1}{2}\sum _{k\in {\mathbb {Z}}}\left( k+\frac{1}{2}\right) :{\Psi }_{n-k }{\Psi }_{k}: &{} \quad \text {if }n\ne 0\\ \frac{1}{16} \; \mathsf {id}+\sum _{k=1}^{\infty }k{\Psi }_{-k}{\Psi }_{k} &{} \quad \text {if } n=0 \end{array}\right. } \end{aligned}$$

where $:{\Psi }_{j}{\Psi }_{k}:$ is defined as ${\Psi }_{j}{\Psi }_{k}$ if $j\le k$ and as $-{\Psi }_{k}{\Psi }_{j}$ otherwise.

##### Lemma 4.20

For any $s\in {\mathbb {Z}}$, we have

$$\begin{aligned} \sum _{k\in {\mathbb {Z}}+ \frac{1}{2}} {(\pm \overset{\underleftrightarrow { {k} }}{{{{{\Psi }_{s-k}}}{{{\Psi }_{k}}}}}}) = 0 \qquad \text { and } \qquad \sum _{k\in {\mathbb {Z}}}{(\pm \overset{\underleftrightarrow { {k} }}{{{{{\Psi }_{s-k}}}{{{\Psi }_{k}}}}}}) = \frac{1}{2} \delta _{s,0} \; \mathsf {id}. \end{aligned}$$

##### Proof

Rewriting the sum in terms of two equal pieces and changing variables to $k' = s-k$ in one of the two, we get

$$\begin{aligned} \sum _{k} {(\pm \overset{\underleftrightarrow { {k} }}{{{{{\Psi }_{s-k}}}{{{\Psi }_{k}}}}}}) = \frac{1}{2} \sum _{k} {(\pm \overset{\underleftrightarrow { {k} }}{{{{{\Psi }_{s-k}}}{{{\Psi }_{k}}}}}}) + \frac{1}{2} \sum _{k'} {(\pm \overset{\underleftrightarrow { {s-k'} }}{{{{{\Psi }_{k'}}}{{{\Psi }_{s-k'}}}}}}) . \end{aligned}$$

We conclude the asserted formulas by the following cancellations among the two pieces

$$\begin{aligned} {(\pm \overset{\underleftrightarrow { {k} }}{{{{{\Psi }_{s-k}}}{{{\Psi }_{k}}}}}}) + {(\pm \overset{\underleftrightarrow { {s-k} }}{{{{{\Psi }_{k}}}{{{\Psi }_{s-k}}}}}}) = {\left\{ \begin{array}{ll} \mathsf {id}&{} \quad \text {if }s=k=0 \\ 0 &{} \quad \text {otherwise}. \end{array}\right. } \end{aligned}$$

The case $s=k=0$ here is simply the anticommutation relation $2 \, {\Psi }_{0} {\Psi }_{0} = \mathsf {id}$ of Proposition 4.16. In the cases where $k, s-k$ have the same sign, the cancellation follows from the anticommutation relation ${\Psi }_{s-k} {\Psi }_{k} + {\Psi }_{k} {\Psi }_{s-k} = 0$. In the cases where $k, s-k$ have different signs, the cancellation follows from the definition of ${(\pm \overset{\underleftrightarrow { {k} }}{{{{A}}{{B}}}}})$. $\square$

##### Lemma 4.21

For any $n\in {\mathbb {Z}}$, if $j,k\in {\mathbb {Z}}+\frac{1}{2}$ we have

$$\begin{aligned} \Big [ {\mathsf {L}}_{n}^{\mathrm {even}}, {(\pm \overset{\underleftrightarrow { {k} }}{{{{{\Psi }_{j}}}{{{\Psi }_{k}}}}}}) \Big ] = - \big ( j+\frac{n}{2} \big ) {(\pm \overset{\underleftrightarrow { {k} }}{{{{{\Psi }_{n+j}}}{{{\Psi }_{k}}}}}}) - \big ( k+\frac{n}{2} \big ) {(\pm \overset{\underleftrightarrow { {k} }}{{{{{\Psi }_{j}}}{{{\Psi }_{n+k}}}}}}) . \end{aligned}$$

Also, for any $n,j,k\in {\mathbb {Z}}$, we have

$$\begin{aligned} \Big [ {\mathsf {L}}_{n}^{\mathrm {odd}} , {(\pm \overset{\underleftrightarrow { {k} }}{{{{{\Psi }_{j}}}{{{\Psi }_{k}}}}}}) \Big ] = - \big ( j+\frac{n}{2} \big ) {(\pm \overset{\underleftrightarrow { {k} }}{{{{{\Psi }_{n+j}}}{{{\Psi }_{k}}}}}}) - \big ( k+\frac{n}{2} \big ) {(\pm \overset{\underleftrightarrow { {k} }}{{{{{\Psi }_{j}}}{{{\Psi }_{n+k}}}}}}) . \end{aligned}$$

##### Proof

It is sufficient to prove the statements without the reordering ${(\pm \overset{\underleftrightarrow { {k} }}{{{{\cdot }}{{\cdot }}}}})$, since possible reordering only amounts to interchanging *j* and *k* and changing signs. Moreover, the proofs of both statements are completely similar, so we only detail the calculation for the first one. Using Definition 4.17 and the anticommutation relations of Proposition 4.16, and the identity

$$\begin{aligned} AB CD - CD AB= & {} A (BC+CB) D - (AC+CA) B D + C A (BD+DB) \\&- C (AD+DA) B , \end{aligned}$$

we get

$$\begin{aligned} \Big [ {\mathsf {L}}_{n}^{\mathrm {even}}, {\Psi }_{j} {\Psi }_{k} \Big ]&= \frac{1}{2} \sum _{\ell \in {\mathbb {Z}}+\frac{1}{2}} \ell \, \Big ( {(\pm \overset{\underleftrightarrow { {\ell } }}{{{{{\Psi }_{n-\ell }}}{{{\Psi }_{\ell }}}}}}) {\Psi }_{j} {\Psi }_{k} - {\Psi }_{j} {\Psi }_{k} {(\pm \overset{\underleftrightarrow { {\ell } }}{{{{{\Psi }_{n-\ell }}}{{{\Psi }_{\ell }}}}}}) \Big ) \\&= \frac{1}{2} \sum _{\ell \in {\mathbb {Z}}+\frac{1}{2}} \ell \, \Big ( \delta _{\ell +j,0} \, {\Psi }_{n - \ell } {\Psi }_{k} - \delta _{n-\ell +j,0} \, {\Psi }_{\ell } {\Psi }_{k} \\&\quad + \delta _{\ell +k,0} \, {\Psi }_{j} {\Psi }_{n - \ell } - \delta _{n-\ell +k,0} \, {\Psi }_{j} {\Psi }_{\ell } \Big ) \\&= \frac{1}{2} \Big ( (-j) \, {\Psi }_{n - \ell } {\Psi }_{k} - (n+j) \, {\Psi }_{\ell } {\Psi }_{k} + (-k) \, {\Psi }_{j} {\Psi }_{n - \ell }- (n+k) \, {\Psi }_{j} {\Psi }_{\ell } \Big ) \\&= - \big ( j+\frac{n}{2} \big ) \, {\Psi }_{n+j}{\Psi }_{k} - \big ( k+\frac{n}{2} \big ) \, {\Psi }_{j}{\Psi }_{n+k} . \end{aligned}$$

$$\square $$

##### Theorem 4.22

The operators $\left( {\mathsf {L}}_{n}^{\mathrm {even}}\right) _{n\in {\mathbb {Z}}}$ form a representation of the Virasoro algebra with central charge ${c=\frac{1}{2}}$.

##### Proof

Using the definition of the Virasoro mode ${\mathsf {L}}_{m}^{\mathrm {even}}$ and Lemma 4.21, we calculate

$$\begin{aligned}{}[{\mathsf {L}}_{n}^{\mathrm {even}} , {\mathsf {L}}_{m}^{\mathrm {even}}]&= \frac{1}{2} \sum _{k\in {\mathbb {Z}}+\frac{1}{2}} k \, \big [ {\mathsf {L}}_{n}^{\mathrm {even}} , {(\pm \overset{\underleftrightarrow { {k} }}{{{{{\Psi }_{m-k}}}{{{\Psi }_{k}}}}}}) \big ] \\&= \frac{-1}{2} \sum _{k\in {\mathbb {Z}}+\frac{1}{2}} \Big ( k \big ( m-k+\frac{n}{2} \big ) {(\pm \overset{\underleftrightarrow { {k} }}{{{{{\Psi }_{n+m-k}}}{{{\Psi }_{k}}}}}}) + k \big ( k+\frac{n}{2} \big ) {(\pm \overset{\underleftrightarrow { {k} }}{{{{{\Psi }_{m-k}}}{{{\Psi }_{n+k}}}}}}) \Big ) . \end{aligned}$$

Performing the change of variables $\ell = k+n$ in the second part of the sum and then combining the two parts, and using Lemma 4.20, we get

$$\begin{aligned}{}[{\mathsf {L}}_{n}^{\mathrm {even}} , {\mathsf {L}}_{m}^{\mathrm {even}}]&= -\frac{1}{2} \sum _{k\in {\mathbb {Z}}+\frac{1}{2}} k \big ( m-k+\frac{n}{2} \big ) {(\pm \overset{\underleftrightarrow { {k} }}{{{{{\Psi }_{n+m-k}}}{{{\Psi }_{k}}}}}}) \\&\quad - \frac{1}{2} \sum _{\ell \in {\mathbb {Z}}+\frac{1}{2}} (\ell - n) \big ( \ell -\frac{n}{2} \big ){(\pm \overset{\underleftrightarrow { {\ell -n} }}{{{{{\Psi }_{n+m-\ell }}}{{{\Psi }_{\ell }}}}}}) \\&= -\frac{1}{2} \sum _{k\in {\mathbb {Z}}+\frac{1}{2}} \Big ( km-kn+\frac{n^2}{2} \Big ) {(\pm \overset{\underleftrightarrow { {k} }}{{{{{\Psi }_{n+m-k}}}{{{\Psi }_{k}}}}}}) + {\mathcal {B}}_{n,m} \\&= (n-m) \; \frac{1}{2} \sum _{k\in {\mathbb {Z}}+\frac{1}{2}} k \, {(\pm \overset{\underleftrightarrow { {k} }}{{{{{\Psi }_{n+m-k}}}{{{\Psi }_{k}}}}}}) + {\mathcal {B}}_{n,m} \\&= (n-m) \; {\mathsf {L}}_{n+m}^{\mathrm {even}} + {\mathcal {B}}_{n,m} , \end{aligned}$$

where

$$\begin{aligned} {\mathcal {B}}_{n,m}&= - \frac{1}{2} \sum _{\ell \in {\mathbb {Z}}+\frac{1}{2}} (\ell - n) \big ( \ell -\frac{n}{2} \big ) \Big ( {(\pm \overset{\underleftrightarrow { {\ell -n} }}{{{{{\Psi }_{n+m-\ell }}}{{{\Psi }_{\ell }}}}}}) - {(\pm \overset{\underleftrightarrow { {\ell } }}{{{{{\Psi }_{n+m-\ell }}}{{{\Psi }_{\ell }}}}}}) \Big ) . \end{aligned}$$

The theorem will be proven, if we show that ${\mathcal {B}}_{n,m} = \frac{n^3-n}{24} \delta _{n+m,0} \, \mathsf {id}$. Note that in the sum over $\ell$ which defines ${\mathcal {B}}_{n,m}$, whenever either $\ell , \ell -n \ge 0$ or $\ell , \ell -n < 0$ the two terms directly cancel. The finitely many remaining terms are simplified using Proposition 4.16 to constant multiples of the identity. For example for $n > 0$, we get

$$\begin{aligned} {\mathcal {B}}_{n,m}&= - \frac{1}{2} \sum _{\begin{array}{c} \ell \in {\mathbb {Z}}+\frac{1}{2} \\ 0< \ell< n \end{array}} (\ell - n) \big ( \ell -\frac{n}{2} \big ) \Big ( \underbrace{ - {\Psi }_{n+m-\ell }{\Psi }_{\ell } - {\Psi }_{n+m-\ell }{\Psi }_{\ell } }_{ = - \delta _{n+m,0} \, \mathsf {id}} \Big ) \\&= + \frac{1}{2} \Big ( \sum _{\begin{array}{c} \ell \in {\mathbb {Z}}+\frac{1}{2} \\ 0< \ell < n \end{array}} (\ell - n) \big ( \ell -\frac{n}{2} \big ) \Big ) \; \delta _{n+m,0} \; \mathsf {id}\; = \; \frac{n^3-n}{24} \delta _{n+m,0} \, \mathsf {id}. \end{aligned}$$

The case $n<0$ is similar. $\square$

##### Theorem 4.23

The operators $\left( {\mathsf {L}}_{n}^{\mathrm {odd}}\right) _{n\in {\mathbb {Z}}}$ form a representation of the Virasoro algebra with central charge ${c=\frac{1}{2}}$.

##### Proof

As in the proof of Theorem 4.22, we need to show that

$$\begin{aligned} \big [ {\mathsf {L}}_{n}^{\mathrm {odd}} , {\mathsf {L}}_{m}^{\mathrm {odd}} \big ] - (n-m) \, {\mathsf {L}}_{n+m}^{\mathrm {odd}} \; = \; \frac{n^3-n}{24} \, \delta _{n,-m} \; \mathsf {id}. \end{aligned}$$

If $n,m\ne 0$ and $n+m\ne 0$, the proof that the left hand side vanishes is exactly the same as in Theorem 4.22 (the formulas for ${\mathsf {L}}_{n}^{\mathrm {odd}}, {\mathsf {L}}_{m}^{\mathrm {odd}}, {\mathsf {L}}_{n+m}^{\mathrm {odd}}$ are the same, and moreover the anticommutation result of Proposition 4.16, the commutation result of Lemma 4.21 and the cancellation result of Lemma 4.20 apply in exactly the same way). If $n=m=0$, the result is trivial. If either *n* or *m* is zero, simply observe that the extra term $\frac{1}{16} \, \mathsf {id}$ commutes with anything, and hence that there is no difference.

The only non-trivial case to check is hence $n>0$ and $m=-n$. We calculate, using now the second statement in Lemma 4.20,

$$\begin{aligned} {[}{\mathsf {L}}_{n}^{\mathrm {odd}} , {\mathsf {L}}_{-n}^{\mathrm {odd}}]&= \frac{1}{2} \sum _{k\in {\mathbb {Z}}} k \, \big [ {\mathsf {L}}_{n}^{\mathrm {odd}} , {(\pm \overset{\underleftrightarrow { {k} }}{{{{{\Psi }_{-n-k}}}{{{\Psi }_{k}}}}}}) \big ] \\&= \frac{-1}{2} \sum _{k\in {\mathbb {Z}}} \Big ( k \big ( -k-\frac{n}{2} \big ) {(\pm \overset{\underleftrightarrow { {k} }}{{{{{\Psi }_{-k}}}{{{\Psi }_{k}}}}}}) + k \big ( k+\frac{n}{2} \big ) {(\pm \overset{\underleftrightarrow { {k} }}{{{{{\Psi }_{-n-k}}}{{{\Psi }_{n+k}}}}}}) \Big ) \\&= -\frac{1}{2} \sum _{k\in {\mathbb {Z}}} k \big ( -k-\frac{n}{2} \big ) {(\pm \overset{\underleftrightarrow { {k} }}{{{{{\Psi }_{-k}}}{{{\Psi }_{k}}}}}}) - \frac{1}{2} \sum _{\ell \in {\mathbb {Z}}} (\ell - n) \big ( \ell -\frac{n}{2} \big ) {(\pm \overset{\underleftrightarrow { {\ell -n} }}{{{{{\Psi }_{-\ell }}}{{{\Psi }_{\ell }}}}}}) \\&= -\frac{1}{2} \sum _{k\in {\mathbb {Z}}} \Big ( -2 k n + \frac{n^2}{2} \Big ) {(\pm \overset{\underleftrightarrow { {k} }}{{{{{\Psi }_{-k}}}{{{\Psi }_{k}}}}}}) + {\mathcal {B}}'_{n} \\&= 2n \; \frac{1}{2} \sum _{k\in {\mathbb {Z}}} k \, {(\pm \overset{\underleftrightarrow { {k} }}{{{{{\Psi }_{-k}}}{{{\Psi }_{k}}}}}}) - \frac{n^2}{8} \, \mathsf {id}+ {\mathcal {B}}'_{n} \\&= 2n \; {\mathsf {L}}_{0}^{\mathrm {odd}} - \frac{n}{8} \, \mathsf {id}- \frac{n^2}{8} \, \mathsf {id}+ {\mathcal {B}}'_{n} , \end{aligned}$$

where

$$\begin{aligned} {\mathcal {B}}'_{n}&= - \frac{1}{2} \sum _{\ell \in {\mathbb {Z}}} (\ell - n) \big ( \ell -\frac{n}{2} \big ) \Big ( {(\pm \overset{\underleftrightarrow { {\ell -n} }}{{{{{\Psi }_{-\ell }}}{{{\Psi }_{\ell }}}}}}) - {(\pm \overset{\underleftrightarrow { {\ell } }}{{{{{\Psi }_{-\ell }}}{{{\Psi }_{\ell }}}}}}) \Big ) \\&= - \frac{1}{2} \sum _{\ell = 0}^{n-1} (\ell - n) \big ( \ell -\frac{n}{2} \big ) \Big ( - {\Psi }_{\ell }{\Psi }_{-\ell } - {\Psi }_{-\ell }{\Psi }_{\ell } \Big ) \\&= \Big ( \frac{1}{2} \sum _{\ell = 0}^{n-1} (\ell - n) \big ( \ell -\frac{n}{2} \big ) \Big ) \; \mathsf {id}\; = \; \frac{1}{24} \big ( n^3 + 3 n^2 + 2 n \big ) \; \mathsf {id}. \end{aligned}$$

Now simplifying, we obtain the desired form of the commutator,

$$\begin{aligned} {[}{\mathsf {L}}_{n}^{\mathrm {odd}} , {\mathsf {L}}_{-n}^{\mathrm {odd}}] \; = \; 2n \, {\mathsf {L}}_{0}^{\mathrm {odd}} + \frac{n^3-n}{24} \, \mathsf {id}. \end{aligned}$$

$$\square $$

We can now construct the Virasoro representation on the Ising model local fields, by putting together the even and odd sectors treated above.

##### Theorem 4.24

Setting ${\mathsf {L}}_{n}^{{\mathcal {I}}} := {\mathsf {L}}_{n}^{\mathrm {even}} \oplus {\mathsf {L}}_{n}^{\mathrm {odd}}$, we have that the operators $\left( {\mathsf {L}}_{n}^{{\mathcal {I}}}\right) _{n \in {\mathbb {Z}}}$ form a representation of the Virasoro algebra with central charge ${c=\frac{1}{2}}$ on ${\mathfrak {F}}_{{\mathcal {I}}}$, the space of Ising lattice local fields modulo null fields.

##### Proof

This is immediate from Theorems 4.22 and 4.23. $\square$

## Discrete Complex Analysis Proofs

### Integer monomial construction

The construction of the positive integer monomials is quite straightforward:

#### Lemma 5.1

There exists a unique family of functions ${z^{[{p}]}} :{{\mathbb {C}}_{{\delta }}^{\diamond }}\cup {{\mathbb {C}}_{{\delta }}^{{\mathfrak {m}}}}\rightarrow {\mathbb {C}}$ for $p\ge 0$ such that $z^{\left[ 0\right] }\equiv 1$, $z^{[1]}={\delta }^{-1}z$, ${\partial }_{{\delta }}{z^{[{p}]}} = p {z^{[{p-1}]}}$, ${0^{[{p}]}} = 0$ for all $p\ge 1$, and $\left( \pm \frac{{\delta }}{2}\right) ^{[p]}=\left( \pm \frac{\mathbb {i}{\delta }}{2}\right) ^{[p]}$ for all $p\ge 2$. These functions satisfy the properties 1, 2, 3, 4, 7, 8 of Proposition 2.1.

#### Proof

It is not hard to check by induction on *p*, using discrete integration, that the functions $${z_{{\mathfrak {m}}}^{[{p}]}}$$ and $${z_{\diamond }^{[{p}]}}$$ are both discrete holomorphic and satisfy the relevant symmetry properties, and are uniquely determined by the values near 0. The convergence as $${\delta }\rightarrow 0$$ follows by discrete integration. The set of medial points where $${z^{[{p+1}]}}$$ vanishes is easily shown to be the set of neighbors of the diamond points where $${z^{[{p}]}} $$ vanishes (and vice versa), thus showing that the set of points where $${z^{[{p}]}} $$ vanishes grows with *p*. $$\square $$

The construction of the negative integer monomials is quite simple, too.

#### Lemma 5.2

There exists a unique family of functions ${z^{[{p}]}} :{{\mathbb {C}}_{{\delta }}^{\diamond }}\cup {{\mathbb {C}}_{{\delta }}^{{\mathfrak {m}}}}\rightarrow {\mathbb {C}}$ for $p<0$ such that ${\partial }_{{\delta }}{z^{[{p}]}} = p {z^{[{p-1}]}}$, ${0^{[{p}]}} = 0$ for all $p<0$ and with ${{{\bar{\partial }}}}_{{\delta }}{z_{{\mathfrak {m}}}^{[{-1}]}} = {2\pi } {\mathbf {1}}_{\left\{ 0 \right\} }$ and , such that ${z^{[{-1}]}} \rightarrow 0$ as $z \rightarrow \infty$. These functions satisfy the Properties 1, 6, and 8 of Proposition 2.1.

#### Proof

Recall that the discrete Cauchy kernel ${\mathsf {k}}:{{\mathbb {C}}_{{\delta }}^{{\mathfrak {m}}}}\rightarrow {\mathbb {C}}$ with a discrete pole at 0 is the unique function ${\mathsf {k}}:{{\mathbb {C}}_{{\delta }}^{{\mathfrak {m}}}}\rightarrow {\mathbb {C}}$ such that ${{{\bar{\partial }}}}_{{\delta }}{\mathsf {k}}= {\mathbf {1}}_{\left\{ 0 \right\} }$ and ${\mathsf {k}}$ decays at infinity [ChSm11, Ken00]—it can be constructed as the ${\partial }_{{\delta }}$-derivative of the random walk potential kernel ${\mathsf {a}}:{{\mathbb {C}}_{{\delta }}}\rightarrow {\mathbb {R}}$ (see [LaLi10]), extended as zero on the dual ${{\mathbb {C}}_{{\delta }}^*}$. The function ${z_{{\mathfrak {m}}}^{[{-1}]}}$ is then defined as $2 \pi {\mathsf {k}}$ on ${{\mathbb {C}}_{{\delta }}^{{\mathfrak {m}}}}$. Given this, the function ${z_{\diamond }^{[{-1}]}}$ can be constructed by ${z^{[{-1}]}} =\frac{1}{4} \sum {z_{x}^{[{-1}]}}$ where the sum is over the four medial neighbors $z_{x} \in {{\mathbb {C}}_{{\delta }}^{{\mathfrak {m}}}}$ to $z \in {{\mathbb {C}}_{{\delta }}^{\diamond }}$; it is easy to check that they satisfy Properties 1 (symmetry) and 6 (decay at infinity), and Property 8 (convergence) is given by standard discrete complex analysis techniques [ChSm11]. The uniqueness for $p=-1$ follows from the maximum principle. The other functions are readily determined by repeated ${\partial }_{{\delta }}$-differentiation, and it is elementary to check their properties. $\square$

### Construction of discrete square roots

In this subsection, we construct the discrete analogues ${z_{{\mathfrak {m}}}^{[{-\frac{1}{2}}]}}, {z_{{\mathfrak {m}}}^{[{\frac{1}{2}}]}} :{\left[ {{\mathbb {C}}_{{\delta }}^{{\mathfrak {m}}}},0 \right] } \rightarrow {\mathbb {C}}$ of the functions $z \mapsto \frac{1}{\sqrt{z}}$ and $z \mapsto \sqrt{z}$ that we will need for our construction. For this, we rely on constructions introduced in [CHI15, GHP19], and modifications thereof.

Let us denote by ${{\mathbb {C}}_{{\delta }}^{{\mathfrak {c}}{\mathfrak {m}}}}:= {{\mathbb {C}}_{{\delta }}^{{\mathfrak {c}}}}\cup {{\mathbb {C}}_{{\delta }}^{{\mathfrak {m}}}}$ the lattice formed by the corners and midpoints of edges of ${{\mathbb {C}}_{{\delta }}}$. We say that a function $F :{{\mathbb {C}}_{{\delta }}^{{\mathfrak {c}}{\mathfrak {m}}}}\rightarrow {\mathbb {C}}$ is *s-holomorphic* if for any $c\in {{\mathbb {C}}_{{\delta }}^{{\mathfrak {c}}}}$ adjacent to $z \in {{\mathbb {C}}_{{\delta }}^{{\mathfrak {m}}}}, v \in {{\mathbb {C}}_{{\delta }}}, f \in {{\mathbb {C}}_{{\delta }}^*}$ we have

5.1 $$\begin{aligned} F(c) =\frac{1}{2}\left( F(z) +\overline{{\nu }(c)} {\bar{F}}(z) \right) , \end{aligned}$$

where ${\nu }(c) =\left( v-f\right) /\left| v-f\right|$. This requirement will be called a “projection relation”, since it can be interpreted as stating that the value of the function at a corner *c* is the projection of the complex value at an adjacent midedge *z* to the line $\sqrt{{\bar{{\nu }}}(c)} \, {\mathbb {R}}\subset {\mathbb {C}}$ in the complex plane.

**Fig. 11:**
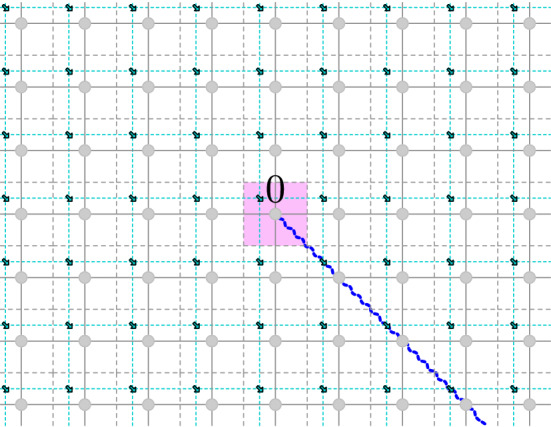
The lattice of those corners whose direction is ${\nu }(c) = \varrho$. The wiggly line denotes the branch cut $\varrho {\mathbb {R}}_+$, and the corner immediately on the North-West of 0 is $\frac{-1+\mathbb {i}}{4}{\delta }$. The shaded square is *Q*

We extend the notion of s-holomorphicity to functions defined on ${\left[ {{\mathbb {C}}_{{\delta }}^{{\mathfrak {c}}{\mathfrak {m}}}}, 0 \right] }$ by defining adjacent as adjacent and on the same sheet (Fig. 11).

#### Discrete inverse square root

In this paragraph, we introduce the discrete analogue of the function $z^{-\frac{1}{2}}$ on the medial lattice.

##### Lemma 5.3

Set $\kappa := e^{\pi \mathbb {i}/8}$, and $\varrho := e^{-\pi \mathbb {i}/4}$. There exists a unique s-holomorphic function $K_{{\delta }} :{\left[ {{\mathbb {C}}_{{\delta }}}^{{\mathfrak {c}}{\mathfrak {m}}}, 0 \right] } \rightarrow {\mathbb {C}}$ with $-1$ monodromy around 0 such that $K_{{\delta }}\left( \frac{-1+\mathbb {i}}{4}{\delta }\right) =\kappa$, such that $K_{{\delta }}(c) =0$ for all corners with ${\nu }(c) =\varrho$ on $\varrho {\mathbb {R}}_{+}$ and that decays at infinity.

##### Proof

Set $\Lambda _{{\delta }} := \left\{ c \in {\left[ {{\mathbb {C}}_{{\delta }}^{{\mathfrak {c}}}}, 0 \right] } : {\nu }(c) = \varrho \right\}$ and let $\Lambda _{{\delta }}^{\pm }$ denote the two sheets of $\Lambda _{{\delta }}\setminus \varrho {\mathbb {R}}_{+}$. We construct $K_{{\delta }}$ on $\Lambda _{{\delta }}^{\pm }$ as $\pm \kappa H_{{\delta }}$, where $H_{{\delta }}(c)$ is the (Beurling-type) probability that a simple random walk on $\Lambda _{{\delta }}$ starting from *c* hits the “tip” $\frac{-1+\mathbb {i}}{4}{\delta }$ before the slit $\varrho {\mathbb {R}}_{+}$. The function $K_{{\delta }}$ is clearly discrete harmonic on $\Lambda _{{\delta }}$, and also on the cut $\varrho {\mathbb {R}}_{+}$; indeed, the value on the cut is zero by definition, as is the average of the values of $H_{{\delta }}$ on the two sheets, since they are of opposite sign, by symmetry. That it decays at infinity is standard. The uniqueness follows from the maximum principle using the decay at infinity. Given this, we can then use the same strategy as the proof of Lemma 2.14 (Sect. 3.2.2 and Remark 3.1) of [CHI15], to uniquely reconstruct $K_{{\delta }}$ from values on $\Lambda _{{\delta }}$, by enforcing a $-1$ mondromy around 0.^6^

An important difference with the situation of [CHI15] is that there is no s-holomporphic singularity (failure of s-holomorphicity) of $K_{{\delta }}$ anywhere. Indeed, the point where the discrete harmonicity of $H_{{\delta }}$ fails is $\frac{-1+\mathbb {i}}{4}{\delta }$, which is contained within the (dual lattice) square *Q* of sidelength ${\delta }$ centered at 0. An s-holomorphic singularity occurs at a corner when two conflicting values for a given corner are “suggested” by the two adjacent medial vertices through the projection relation (5.1). In the case of [CHI15], the singularity occurs at a corner which is outside the square *Q*, and the two values suggested by the adjacent medial vertices are opposite (they come from two sheets); in our case, thanks to the fact that branching is at the center of the square *Q*, the s-holomorphicity is preserved: the two values that come naturally from both sheets do not conflict, since the corners for which they “suggest” a value are actually different (i.e. on opposite sheets). Note that this situation is exactly the same as in the proof of Theorem 2.20 in [CHI15] (see in particular the paragraph before that proof); see also Remark 2.6 in [GHP19], where it is explained that discrete holomorphicity may exist despite the failure of harmonicity. $\square$

Define the constant $c_{*} := \sqrt{\frac{\sqrt{2}}{\pi }}$.

##### Lemma 5.4

We have that $\frac{1}{c_{*}\sqrt{{\delta }}}K_{{\delta }}(z) \rightarrow \mathbb {i}z^{-\frac{1}{2}}$ as ${\delta }\rightarrow 0$, uniformly for *z* away from 0.

##### Proof

The convergence technology of the proof of Lemma 2.14 in [CHI15] leads to the proof of convergence of $\frac{1}{\vartheta \left( {\delta }\right) }K_{{\delta }} \rightarrow \mathbb {i}z^{-\frac{1}{2}}$, for a function $\vartheta \left( {\delta }\right) \asymp \sqrt{{\delta }}$ as ${\delta }\rightarrow 0$. But the proof of Lemma 5.15 of [GHP19] gives the stronger estimate that $\vartheta \left( {\delta }\right) \sim \sqrt{\frac{\sqrt{2}{\delta }}{\pi }} = c_{*} \sqrt{{\delta }}$ as ${\delta }\rightarrow 0$ (note that the lattice square side length in those papers is denoted by $\sqrt{2}\,\delta$ whereas lattice square side length in our paper is ${\delta }$). $\square$

We can now define $z_{{\mathfrak {m}}}^{\left[ -\frac{1}{2}\right] }$ on ${\left[ {{\mathbb {C}}_{{\delta }}^{{\mathfrak {m}}}}, 0 \right] }$.

##### Definition 5.5

We define $z_{{\mathfrak {m}}}^{\left[ -\frac{1}{2}\right] }$ on ${\left[ {{\mathbb {C}}_{{\delta }}^{{\mathfrak {m}}}}, 0 \right] }$ by symmetrizing $K_{{\delta }}$ as follows:

$$\begin{aligned} z_{{\mathfrak {m}}}^{\left[ -\frac{1}{2}\right] } := \frac{1}{8 \mathbb {i}c_{*}} \sum _{k=0}^{7} e^{\mathbb {i}k \pi / 4 } \, K_{{\delta }}\left( \mathbb {i}^{k}z\right) . \end{aligned}$$

##### Remark 5.6

One can show that the function $z_{{\mathfrak {m}}}^{\left[ -\frac{1}{2}\right] }$ is the unique function on ${\left[ {{\mathbb {C}}_{{\delta }}^{{\mathfrak {m}}}}, 0 \right] }$ that is discrete holomorphic on ${\left[ {{\mathbb {C}}_{{\delta }}^{{\mathfrak {m}}}}, 0 \right] }$ except at 0, that decays at infinity and shares the same 90 degree rotation symmetries as $z\mapsto 1/\sqrt{z}$ and is purely real on the positive axis.

##### Lemma 5.7

We have that the values of $z_{{\mathfrak {m}}}^{\left[ -\frac{1}{2}\right] }$ at $e^{\frac{\pi \mathbb {i}}{2}k}\frac{{\delta }}{2}$ for $k=0,\ldots ,7$ are given by $Ce^{-\frac{\pi \mathbb {i}}{4}k}$ for some constant $C>0$.

##### Proof

This follows from a direct calculation and symmetry. $\square$

#### Discrete square root

For the construction of the discrete square root, we rely on a discrete analogue of the real part of the square root function (on the 45 degree-rotated and factor $\sqrt{2}$ scaled square lattice $(\delta + \mathbb {i}\delta ) {\mathbb {Z}}^2 = \sqrt{2} \varrho \, {{\mathbb {C}}_{{\delta }}}$), denoted by $G_{{\left[ {{\mathbb {C}}_{{\delta }}}, 0 \right] }}$, defined in [GHP19], following [CHI15]. It is constructed by essentially integrating the harmonic measure used to define the discrete version of $z^{-1/2}$. In this section, we first extend $G_{{\left[ {{\mathbb {C}}_{{\delta }}}, 0 \right] }}$ to an s-holomorphic function (on the medial and corner lattices of $(\delta + \mathbb {i}\delta ) {\mathbb {Z}}^2$, with the same notation and conventions as in  [GHP19]), and then define our function $G_{{\delta }}$ on ${\left[ {{\mathbb {C}}_{{\delta }}^{{\mathfrak {m}}}}, 0 \right] }$. While there are a few differences (e.g., the choice of the lattice), our function is otherwise very similar.

Let us start by the extension of $G_{{\left[ {{\mathbb {C}}_{{\delta }}}, 0 \right] }}$ to the double cover ${\left[ {{\mathbb {C}}_{{\delta }}}, 0 \right] }$ (with the notation and 45-degree rotated lattice of  [GHP19]) into an s-holomorphic function.

Define the constant ${\tilde{c}}_{*}=\frac{1}{2 \sqrt{\pi }}$.

##### Lemma 5.8

With the notation and conventions of  [GHP19], the function $G_{{\left[ {{\mathbb {C}}_{{\delta }}}, 0 \right] }}$ defined on ${\mathcal {V}}_{{\left[ {{\mathbb {C}}_{{\delta }}}, 0 \right] }}^{1}$ admits a unique s-holomorphic extension with $-1$ monodromy to ${\mathcal {V}}_{{\left[ {{\mathbb {C}}_{{\delta }}}, 0 \right] }}^{\mathrm {{\mathfrak {c}}{\mathfrak {m}}}}$, which vanishes on ${\mathcal {V}}_{{\left[ {{\mathbb {C}}_{{\delta }}}, 0 \right] }}^{\mathbb {i}}\cap {\mathbb {R}}_{+}$ and on ${\mathcal {V}}_{{\left[ {{\mathbb {C}}_{{\delta }}}, 0 \right] }}^{1} \cap {\mathbb {R}}_{-}$. We have that $\frac{1}{{\tilde{c}}_{*}\sqrt{{\delta }}} G_{{\left[ {{\mathbb {C}}_{{\delta }}}, 0 \right] }}(z) \rightarrow \sqrt{z}$ as ${\delta }\rightarrow 0$ uniformly on the compact subsets of ${\left[ {{\mathbb {C}}_{{\delta }}}, 0 \right] }$.

##### Proof

This follows from [GHP19]. See Definition 3.24 in Sect. 3.2.4 (‘Auxiliary Functions’) for the construction, Remark 3.25 in Sect. 3.2.4 for the proof of s-holomorphicity, and Lemma 5.16 in Sect. 5.4.1 (‘Convergence of the Full-plane Observable’) for the proof of convergence. The fact that the function vanishes on ${\mathcal {V}}_{{\left[ {{\mathbb {C}}_{{\delta }}}, 0 \right] }}^{\mathbb {i}} \cap {\mathbb {R}}_{+}$ and on ${\mathcal {V}}_{{\left[ {{\mathbb {C}}_{{\delta }}}, 0 \right] }}^{1} \cap {\mathbb {R}}_{-}$ comes directly from the construction. $\square$

We can now define the function $G_{{\delta }}$ appropriate for our setup, as a 45-degree rotated version of $G_{{\left[ {{\mathbb {C}}_{{\delta }}}, 0 \right] }}$:

##### Definition 5.9

We define $G_{{\delta }} :{\left[ {{\mathbb {C}}_{{\delta }}^{{\mathfrak {m}}}}, 0 \right] } \rightarrow {\mathbb {C}}$ by $G_{{\delta }}(z) := e^{\frac{\pi \mathbb {i}}{8}} G_{{\left[ {{\mathbb {C}}_{{\delta }}}, 0 \right] }} (\sqrt{2}\varrho z)$.^7^

The most important features of $G_{{\delta }}$ are summarized in the following.

##### Proposition 5.10

The function $G_{{\delta }}$ is s-holomorphic on ${\left[ {{\mathbb {C}}_{{\delta }}^{{\mathfrak {m}}}}, 0 \right] }$, vanishes at $\frac{1-\mathbb {i}}{4} \, {\delta }$, and is “discrete holomorphic at 0”, i.e.,

$$\begin{aligned} G_{{\delta }} \Big (\frac{{\delta }}{2} e^{\mathbb {i}0} \Big ) - G_{{\delta }} \Big (\frac{{\delta }}{2} e^{\mathbb {i}\pi } \Big ) + \mathbb {i}\, G_{{\delta }} \Big (\frac{{\delta }}{2} e^{\mathbb {i}\pi / 2} \Big ) - \mathbb {i}\, G_{{\delta }} \Big (\frac{{\delta }}{2} e^{\mathbb {i}3 \pi / 2} \Big ) = 0 . \end{aligned}$$

Moreover, we have that $\frac{1}{2^{1/4} {\tilde{c}}_{*} \sqrt{{\delta }}}G_{{\delta }}(z) \rightarrow \sqrt{z}$ as $z \rightarrow \infty$ uniformly on the compact subsets of ${\left[ {\mathbb {C}},0 \right] }$.

##### Proof

The vanishing of $G_{{\delta }}$ at $\frac{1-\mathbb {i}}{4}{\delta }$ is direct from its construction based on $G_{{\left[ {{\mathbb {C}}_{{\delta }}}, 0 \right] }}$. The discrete holomorphicity at 0 follows from this: a failure of holomorphicity at 0 would come from an incompatibility between the opposite values coming from the different sheets at a given corner; here, since one of the corner values is zero here, this problem is absent. The convergence follows from Lemma 5.8. $\square$

We are now in position to define $z_{{\mathfrak {m}}}^{\left[ \frac{1}{2}\right] }$:

##### Definition 5.11

We define $z_{{\mathfrak {m}}}^{\left[ \frac{1}{2}\right] }$ on ${\left[ {{\mathbb {C}}_{{\delta }}^{{\mathfrak {m}}}}, 0 \right] } \rightarrow {\mathbb {C}}$ by

$$\begin{aligned} z^{\left[ \frac{1}{2}\right] } := \frac{1}{8 \, 2^{1/4} \, {\tilde{c}}_*} \sum _{k=0}^{7} e^{-\mathbb {i}k \pi / 4} \, G_{{\delta }}\left( \mathbb {i}^{k}z\right) . \end{aligned}$$

##### Lemma 5.12

Suppose a function $f :{\left[ {{\mathbb {C}}_{{\delta }}^{{\mathfrak {m}}}}, 0 \right] } \rightarrow {\mathbb {C}}$ has the same 90 degree rotational symmetry as $z \mapsto z^{m/2}$ for *m* odd (and in particular $-1$ monodromy) and is “discrete holomorphic at 0” (in the sense of Proposition 5.10). Then *f* vanishes at each of the medial lattice points adjacent to 0, i.e., at $\frac{{\delta }}{2} \, e^{\mathbb {i}\frac{\pi }{2} k}$ for $k=0,1,\ldots ,7$.

##### Proof

The assumed symmetry implies that $f \big ( \frac{{\delta }}{2} \, e^{\mathbb {i}\frac{\pi }{2} k} \big ) = e^{\mathbb {i}\frac{\pi }{4} m k} \, f \big ( \frac{{\delta }}{2} \big )$. The vanishing of the discrete ${{{\bar{\partial }}}}$-derivative at 0 then amounts to

$$\begin{aligned} 0&= \frac{1}{2} \left( f \big ( \frac{{\delta }}{2} \big ) - f\big ( \frac{{\delta }}{2} \, e^{\mathbb {i}\pi } \big ) \right) + \frac{\mathbb {i}}{2} \left( f\big ( \frac{{\delta }}{2} \, e^{\mathbb {i}\frac{\pi }{2}} \big ) - f\big ( \frac{{\delta }}{2} \, e^{\mathbb {i}\frac{3\pi }{2}} \big ) \right) \\&= \frac{1}{2} \left( 1 + e^{\mathbb {i}\frac{\pi }{4}(2m+4)} + e^{\mathbb {i}\frac{\pi }{4}(m+2)} + e^{\mathbb {i}\frac{\pi }{4}(3m+6)} \right) \; f \big ( \frac{{\delta }}{2} \big ) \, = \,\frac{1}{1-e^{\mathbb {i}\frac{\pi }{4}(m+2)}} \; f \big ( \frac{{\delta }}{2} \big ) . \end{aligned}$$

The constant in front of $f \big ( \frac{{\delta }}{2} \big )$ above is non-zero, so we conclude that *f* vanishes at $\frac{{\delta }}{2}$, and then also at all the points of the form $\frac{{\delta }}{2} \, e^{\mathbb {i}\frac{\pi }{2} k}$. $\square$

##### Proposition 5.13

The function $z_{{\mathfrak {m}}}^{\left[ \frac{1}{2}\right] }$ is discrete holomorphic on ${\left[ {{\mathbb {C}}_{{\delta }}^{{\mathfrak {m}}}}, 0 \right] }$, has the same 90 degree rotation symmetry as $z\mapsto \sqrt{z}$, and therefore vanishes at $\frac{{\delta }}{2} \, e^{\mathbb {i}\frac{\pi }{2} k}$ for $k=0,1,\ldots ,7$. Moreover, we have that $\frac{1}{\sqrt{{\delta }}}z_{{\mathfrak {m}}}^{\left[ \frac{1}{2}\right] } \rightarrow \sqrt{z}$ as ${\delta }\rightarrow 0$, uniformly on compact subsets of $\left[ {\mathbb {C}},0\right]$.

##### Proof

The symmetry is obvious by construction and the discrete holomorphicity at 0 is inherited from that of $G_{{\left[ {{\mathbb {C}}_{{\delta }}}, 0 \right] }}$. The vanishing at the medial lattice points adjacent to 0 then follows from Lemma 5.12. The convergence follows from Proposition 5.10. $\square$

### Half-integer powers

In the previous subsection, we defined the functions $z_{{\mathfrak {m}}}^{\left[ -\frac{1}{2}\right] }$ and $z_{{\mathfrak {m}}}^{\left[ \frac{1}{2}\right] }$. Provided these functions can be differentiated and integrated in the space of functions with a $-1$ monodromy around 0, they uniquely determine the functions $z_{{\mathfrak {m}}}^{\left[ k\right] }$ and $z_{\diamond }^{\left[ k\right] }$ for all $k\in {\mathbb {Z}}+\frac{1}{2}$: as usual, differentiating or integrating shifts the power by 1 and switches the lattice between ${\left[ {{\mathbb {C}}_{{\delta }}^{{\mathfrak {m}}}}, 0 \right] }$ and ${\left[ {{\mathbb {C}}_{{\delta }}^{\diamond }}, 0 \right] }$. The relevant 90 degree symmetries are automatically inherited from those of $z_{{\mathfrak {m}}}^{\left[ -\frac{1}{2}\right] },z_{{\mathfrak {m}}}^{\left[ \frac{1}{2}\right] }$.

#### Lemma 5.14

There is a unique family of functions $${z^{[{p}]}} $$ for $$p\in {\mathbb {Z}}+\frac{1}{2}$$ satisfying the conditions 1–6 and 8 of Proposition 2.3.

#### Proof

As explained above, the negative powers on ${\left[ {{\mathbb {C}}_{{\delta }}^{{\mathfrak {m}}\diamond }}, 0 \right] }$ are obtained by differentiating $z_{{\mathfrak {m}}}^{\left[ -\frac{1}{2}\right] }$ and $z_{{\mathfrak {m}}}^{\left[ \frac{1}{2}\right] }$ and interpreting $0^{[p]}=0$ for all *p*. The decay at infinity of $z_{\diamond }^{\left[ -\frac{1}{2}\right] }$ follows from Harnack type estimates on the derivatives of discrete holomorphic functions [LaLi10], and for $p<-\frac{1}{2}$, the same argument applies. The rest of the properties for $p<0$ are straightforward to check.

The positive powers are obtained by integrating in the space of functions with $$-1$$ monodromy. The discrete holomorphicity of the $${z^{[{p}]}} $$ for all $$p\ge \frac{1}{2}$$ follows by induction from the next lemma. $$\square $$

#### Lemma 5.15

**Fig. 12:**
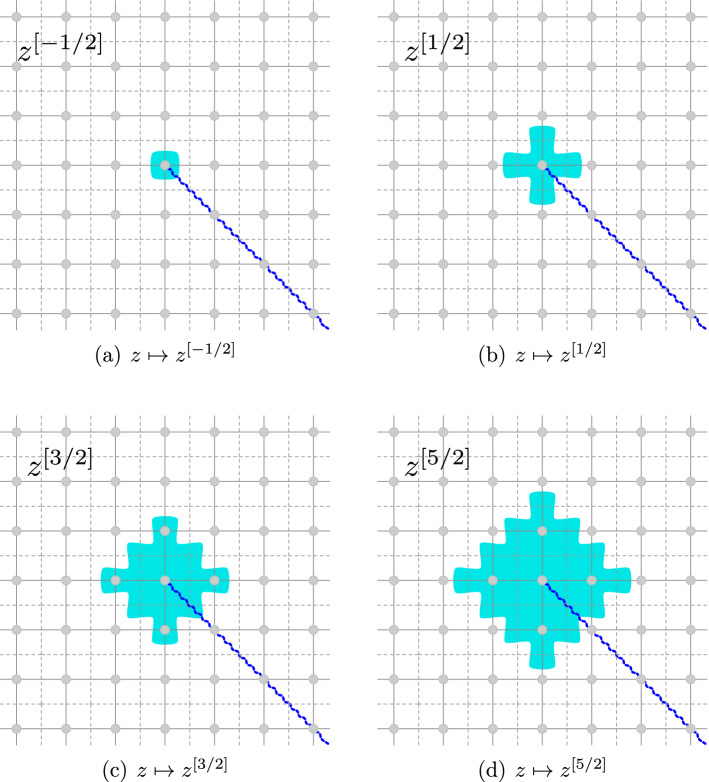
The sets of vertices of ${\left[ {{\mathbb {C}}_{{\delta }}^{{\mathfrak {m}}\diamond }},0 \right] }$ where discrete half-integer monomials vanish are illustrated by the shaded area

We have the following (see Fig. 12):

1. The values of the function $z_{\diamond }^{\left[ \frac{1}{2}\right] }$ are given by $\frac{C \sqrt{\sqrt{2}+1}}{2 }\sqrt{x/{\delta }}$ for *x* living above one of the four dual vertices $\left\{ \frac{\pm 1 \pm \mathbb {i}}{2} {\delta } \right\}$ and by $\frac{C}{2} \sqrt{x/{\delta }}$ for *x* living above one of the four vertices $\pm {\delta }, \pm \mathbb {i}{\delta }$, where *C* is the value of the function $z_{{\mathfrak {m}}}^{[-\frac{1}{2}]}$ at $\frac{{\delta }}{2}$.
2. The functions $z_{{\mathfrak {m}}}^{\left[ \frac{1}{2}\right] }, z_{{\mathfrak {m}}}^{\left[ \frac{3}{2}\right] }$ vanish on the four medial vertices $\left\{ \pm \frac{{\delta }}{2} , \pm \frac{\mathbb {i}{\delta }}{2} \right\}$.
3. The functions $z_{\diamond }^{\left[ \frac{3}{2}\right] } , z_{\diamond }^{\left[ \frac{5}{2}\right] }$ vanish on the nine diamond vertices $\left\{ 0, \frac{\pm 1 \pm \mathbb {i}}{2} {\delta }, \pm {\delta }, \pm \mathbb {i}{\delta } \right\}$.
4. In general, for half-integer $k\ge \frac{1}{2}$, if $z_{\diamond }^{\left[ k\right] }$ vanishes on a neighborhood $\Lambda _{\diamond }$ of the origin, then $z_{{\mathfrak {m}}}^{\left[ k+1\right] }$ vanishes on the set of medial vertices at distance $\frac{{\delta }}{2}$ from $\Lambda _{\diamond }$, and conversely, if $z_{{\mathfrak {m}}}^{\left[ k\right] }$ vanishes on a neighborhood $\Lambda _{{\mathfrak {m}}}$ of the origin, then $z_{\diamond }^{\left[ k+1\right] }$ vanishes on the set of diamond vertices at distance $\frac{{\delta }}{2}$ from $\Lambda _{{\mathfrak {m}}}$.

#### Proof

Item 1 follows from explicit computation and the definition of $z_{{\mathfrak {m}}}^{\left[ -\frac{1}{2}\right] }$. Then Lemma 5.12 gives item 2, and the rest from integration together with Lemma 5.12 again (also keeping in mind that the choice of the antiderivative is made unique by the constraint that the monodromy is $-1$). $\square$

### Contour integrals

#### Lemma 5.16

For any $k,\ell \in {\mathbb {Z}}$ and any large enough contour $\gamma$, we have

#### Proof

If $k+\ell \le 2$, we have the following three sub-cases:

- If $k+\ell <-1$, then since $z_{{\mathfrak {m}}}^{\left[ k\right] }z_{\diamond }^{\left[ \ell \right] } =O\left( \left| z\right| ^{k+\ell }\right) =O\left( \left| z\right| ^{-2}\right)$ we can take the integration contour to be very large, containg $O\left( \left| z\right| \right)$ terms a show that the integral must vanish.
- If $k+\ell =-1$, we can use that $\delta ^{k+\ell }z^{\left[ k\right] }z^{\left[ \ell \right] } \rightarrow z^{k+\ell }$ as ${\delta }\rightarrow 0$ and multiply by ${\delta }$ to make the discrete contour integral converge to a continuous integral, hence yielding the result: the discrete contour integral has the ‘right scaling’ to pass to the limit without renormalization.
- If $k+\ell =0,1,2$, by contour deformation it suffices to consider a contour $\gamma$ which forms the boundary of a large square of the form $\left[ -R,R\right] ^{2}$. For such contours, we can use symmetry to show that the integral must vanish: if $k+\ell =0,2$, we have $z^{\left[ k\right] }z^{\left[ \ell \right] }=\left( -z\right) ^{\left[ k\right] } \left( -z\right) ^{\{\ell \}}$ and hence antipodal contributions will cancel each other (due to the integration element), and if $k+\ell =1$, we have $\left( \mathbb {i}z\right) ^{\left[ k\right] }\left( \mathbb {i}z\right) ^{\left[ \ell \right] } =\mathbb {i}z^{\left[ k\right] }z^{\left[ \ell \right] }$ and hence $\frac{\pi }{4}$ symmetric contributions will cancel each other (due to the integration element being multiplied by $\mathbb {i}$, too).

Let us now look at the case $k+\ell \ge 3$:

- If $k,\ell \ge 0$, the functions are discrete holomorphic everywhere and we conclude readily.
- If $k=-1$, then $\ell \ge 4$, and we can use Stokes’ formula and the fact that $z^{\left[ \ell \right] }$ vanishes at the points where $z^{\left[ k\right] }$ is not discrete holomorphic.
- If $k<-1$, we can use integration by parts (2.3) to raise the power of $z^{\left[ k\right] }$ while decreasing the power of $z^{\left[ \ell \right] }$, until we get back to the previous case.
- The case $\ell \le -1$ is symmetric to the previous two cases.

$$\square $$

#### Lemma 5.17

If either $k,\ell \in {\mathbb {Z}}$ or $k,\ell \in {\mathbb {Z}}+\frac{1}{2}$, denoting ${z_{{\mathfrak {m}}}^{\{{\ell }\}}} = \frac{1}{4}\sum _{x\in \left\{ \pm 1,\pm \mathbb {i}\right\} } { ( z-\frac{x{\delta }}{2} )^{[{\ell }]}}$, then for any large enough contour $\gamma$, we have

#### Proof

The proofs of the cases $k+\ell \le 2$ are exactly the same as the ones of the cases $k+\ell \le 2$ in the proof of Lemma 5.16, even if $k,\ell$ are half-integer: all that is used is the decay at infinity and the 90 degree symmetries. Let us now look at the cases $k+\ell \ge 3$, and show that the integral vanishes. We cover this in three separate cases:

- If $k\ge 1$ and $\ell \ge 0$, to show that the integral vanishes, we distinguish the integer and half-integer cases.
  - For integer $k,\ell$ this is straightforward: the functions ${z_{\diamond }^{[{k}]}}$ and ${z_{{\mathfrak {m}}}^{\{{\ell }\}}}$ are discrete holomorphic everywhere, and hence the contour integral vanishes.
  - For half-integer $k,\ell$, we have that
    - ${z_{\diamond }^{[{k}]}}$ vanishes (at least) on $\left\{ 0,\frac{\pm 1\pm \mathbb {i}}{2}{\delta },\pm {\delta },\pm \mathbb {i}{\delta }\right\}$ and is discrete holomorphic everywhere (since $k\ge \frac{3}{2}$)
    - ${z_{{\mathfrak {m}}}^{\{{\ell }\}}}$ is (at least) discrete holomorphic outside of the nine diamond vertices $\left\{ 0,\frac{\pm 1\pm \mathbb {i}}{2}{\delta },\pm {\delta },\pm \mathbb {i}{\delta }\right\}$: this is inherited from the properties of the function ${z_{\diamond }^{[{\ell }]}}$, shifted by a lattice spacing.
    We can hence deduce that
    $$\begin{aligned} \sum _{z_{{\mathfrak {m}}} \in {\left[ {{\mathbb {C}}_{{\delta }}^{{\mathfrak {m}}}}, 0 \right] }} {z_{{\mathfrak {m}}}^{\{{\ell }\}}} \, {{{\bar{\partial }}}}_{{\delta }}{z_{\diamond }^{[{k}]}}&= 0 \\ \sum _{z_{\diamond }\in {\left[ {{\mathbb {C}}_{{\delta }}^{\diamond }}, 0 \right] }} {z_{\diamond }^{[{k}]}} \, {{{\bar{\partial }}}}_{{\delta }}{z_{{\mathfrak {m}}}^{\{{\ell }\}}}&=0 \end{aligned}$$
    and conclude that the contour integral vanishes.
- If $k<1$, we again distinguish the integer and half-integer cases.
  - For integer $k,\ell$ with $k+\ell \ge 3$ there are three cases:
    - If $k,\ell \ge 0$, then the vanishing of the contour integral is clear by discrete holomorphicity of ${z_{\diamond }^{[{k}]}}$ and ${z_{{\mathfrak {m}}}^{\{{\ell }\}}}$.
    - For $k=-1$ and $k=-2$ and any $\ell \ge 3-k$ we have that ${z_{{\mathfrak {m}}}^{\{{\ell }\}}}$ vanishes on each of the medial vertices where ${{{\bar{\partial }}}}_{{\delta }}{z_{\diamond }^{[{k}]}}$ is non-zero, so the vanishing of the contour integral follows from Stokes’ formula.
    - For $k<-2$ we can integrate by parts an even number of times to reduce to the previous case.
  - For half-integer $k,\ell$, we can use two integrations by parts (on a large enough contour, where both $z_{\diamond }^{\left[ k\right] }$ at $z_{{\mathfrak {m}}}^{\{\ell \} }$ are discrete holomorphic) to raise the power of *k* by 2 units while decreasing the power of $\ell$ by 2 units, keeping $k+\ell$ constant and $\ell \ge 0$ until $k\ge 1$, and reduce this case to the previous one (the reason we use two integrations by parts is to keep the functions on the same lattices: ${\partial }_{{\delta }}{\partial }_{{\delta }}z_{\diamond }^{\left[ k+2\right] } = (k+2) (k+1) z_{\diamond }^{\left[ k\right] }$ and ${\partial }_{{\delta }}{\partial }_{{\delta }}z_{{\mathfrak {m}}}^{\{\ell +2\}} = (\ell +2) (\ell +1) z_{{\mathfrak {m}}}^{\{\ell \} }$).
- The $\ell <1$ case is symmetric to the previous one. $\square$

### Proof of Propositions 2.1 and 2.3

We are now in position to conclude the proofs of the two key propositions about integer and half-integer monomials.

#### Proof of Proposition 2.1

Properties 1–8 were proven in Lemmas 5.1 and 5.2. Property 9 follows from Lemma5.16 and Property 10 follows from Lemma 5.17. $\square$

#### Proof of Proposition 2.3

Properties 1–6 and 8 follow from Lemma 5.14. Property 7 follows from 5.15. Property 10 follows from Lemma 5.17 and the proof of Property 9 is similar but easier. $\square$

## Lattice Fermion Proofs

In this section, we give the proofs of the key properties of the fermions stated in Sect. 3.3.

### Fermions as parafermions

In this subsection, we review the low-temperature expansion of the Ising fermion, which yields to the family of parafermionic observables (see [Smi10b] for instance). This representation is useful to reveal a number of symmetries (it is more canonical in some sense, as it does not depend on a choice of defect line) and it is decisive for the boundary value problem analysis of Ising correlations (see [CHI15, HoSm13, Hon10] for instance).

#### Definition 6.1

Let ${{{\mathscr {C}}}}^{{{{\Omega }}_{{\delta }}}}$ denote the set of even subgraphs^8^ of the dual graph ${{\Omega }}_{{\delta }}^*$, i.e., subsets ${\vartheta }$ of edges of ${{\Omega }}_{{\delta }}^*$ such that every dual vertex $p \in {{\Omega }}_{{\delta }}^*$ is incident to an even number of edges of ${\vartheta }$. For the Ising model with $+$ boundary conditions on ${{\Omega }}_{{\delta }}$, spin configurations ${\sigma }\in \left\{ \pm 1 \right\} ^{{{\Omega }}_{{\delta }}}$ bijectively correspond with even subgraphs ${\vartheta }\in {{{\mathscr {C}}}}^{{{{\Omega }}_{{\delta }}}}$ of the dual graph ${{\Omega }}_{{\delta }}^*$ by the condition that edges of ${\vartheta }$ separate spins with opposite values. For $V \subset {{\Omega }}_{{\delta }}$, we denote by ${\sigma }_{{\vartheta }}^{V}$ the value of the product $\prod _{x\in V}{\sigma }_{x}$ of spins in *V* in the spin configuration ${\sigma }$ corresponding to ${\vartheta }$.

#### Remark 6.2

The Boltzmann weight $e^{-{{\beta }}{\mathcal {H}}[{\sigma }]}$ of a spin configuration ${\sigma }$ is proportional to $e^{-2{{\beta }}\left| {\vartheta }\right| }$, where $|{\vartheta }|$ is the number of edges in the corresponding even subgraph ${\vartheta }$. It follows that we have

$$\begin{aligned} {\mathbb {E}}\! \left[ { \prod _{x\in V}{\sigma }_{x} } \right] = \frac{1}{{\mathcal {Z}}}\sum _{{\vartheta }\in {{{\mathscr {C}}}}^{{{{\Omega }}_{{\delta }}}}} e^{ -2{{\beta }}\left| {\vartheta }\right| }{\sigma }_{{\vartheta }}^{V} , \end{aligned}$$

where

$$\begin{aligned} {\mathcal {Z}} := \sum _{{\vartheta }\in {{{\mathscr {C}}}}^{{{{\Omega }}_{{\delta }}}}}e^{-2{{\beta }}\left| {\vartheta }\right| } . \end{aligned}$$

Let ${\lambda }_{c_{1},c_{2}}$ be a corner defect line in ${{\Omega }}_{{\delta }}$ and, denoting the symmetric difference by $\oplus$, set

$$\begin{aligned} {{{\mathscr {C}}}}_{{c_{1},c_{2}}}^{{{{\Omega }}_{{\delta }}}} := \left\{ {\lambda }_{c_{1},c_{2}} \oplus {\vartheta }\; \Big | \; {\vartheta }\in {{{\mathscr {C}}}}^{{{{\Omega }}_{{\delta }}}} \right\} \end{aligned}$$

which only depends on $c_{1}$ and $c_{2}$, and not on the defect line ${\lambda }_{c_{1},c_{2}}$. An element ${\gamma }\in {{{\mathscr {C}}}}_{{c_{1},c_{2}}}^{{{{\Omega }}_{{\delta }}}}$ will be called a $(c_1,c_2)$-subgraph: it has an even number of edges or corner ends adjacent to every dual vertex, but has one corner end adjacent to both of the the marked corners $c_{1}$ and $c_{2}$. For ${\gamma }\in {{{\mathscr {C}}}}_{{c_{1},c_{2}}}^{{{{\Omega }}_{{\delta }}}}$, we denote (by a slight abuse of notation) by ${\mathbf {W}}\left( {\gamma }\right) \in {\mathbb {R}}/4\pi {\mathbb {Z}}$ the winding of the path in ${\gamma }$ which goes from $c_{1}$ to $c_{2}$, as defined in, e.g., [HoSm13]: if ${\gamma }$ is a simple path, ${\mathbf {W}}\left( {\gamma }\right)$ is defined as in Sect. 3.3.2, and if ‘ambiguities’ arise (i.e. going from $c_{1}$ to $c_{2}$, one has three choices about how to continue the path, left, right or straight) one either turns right or left, but does not go straight, and ${\mathbf {W}}\left( {\gamma }\right)$ modulo $4\pi$ is independent of the choice of the path in ${\gamma }$, as shown in Lemma 1.4 of [HoSm13]. We use the convention that the length $|{\gamma }|$ of ${\gamma }\in {{{\mathscr {C}}}}_{{c_{1},c_{2}}}^{{{{\Omega }}_{{\delta }}}}$ counts only the edges on the dual lattice ${{\Omega }}_{{\delta }}^*$ and not the two corner ends connecting dual vertices to the corners $c_1 , c_2$.

The following lemma connects the two-fermion correlations with the observables of [Hon10, HoSm13, CHI15]. Recall that for a corner $c \in {{\mathbb {C}}_{{\delta }}^{{\mathfrak {c}}}}$ adjacent to vertex $x \in {{\mathbb {C}}_{{\delta }}}$ and dual vertex $p \in {{\mathbb {C}}_{{\delta }}^*}$ we denote ${\nu }(c) := \frac{x-p}{|x-p|}$.

#### Lemma 6.3

Consider the Ising model on a discrete domain ${{\Omega }}_{{\delta }}$ with $+$ boundary conditions. For any corners $c_{1},c_{2}$, we have

#### Proof

**Fig. 13:**
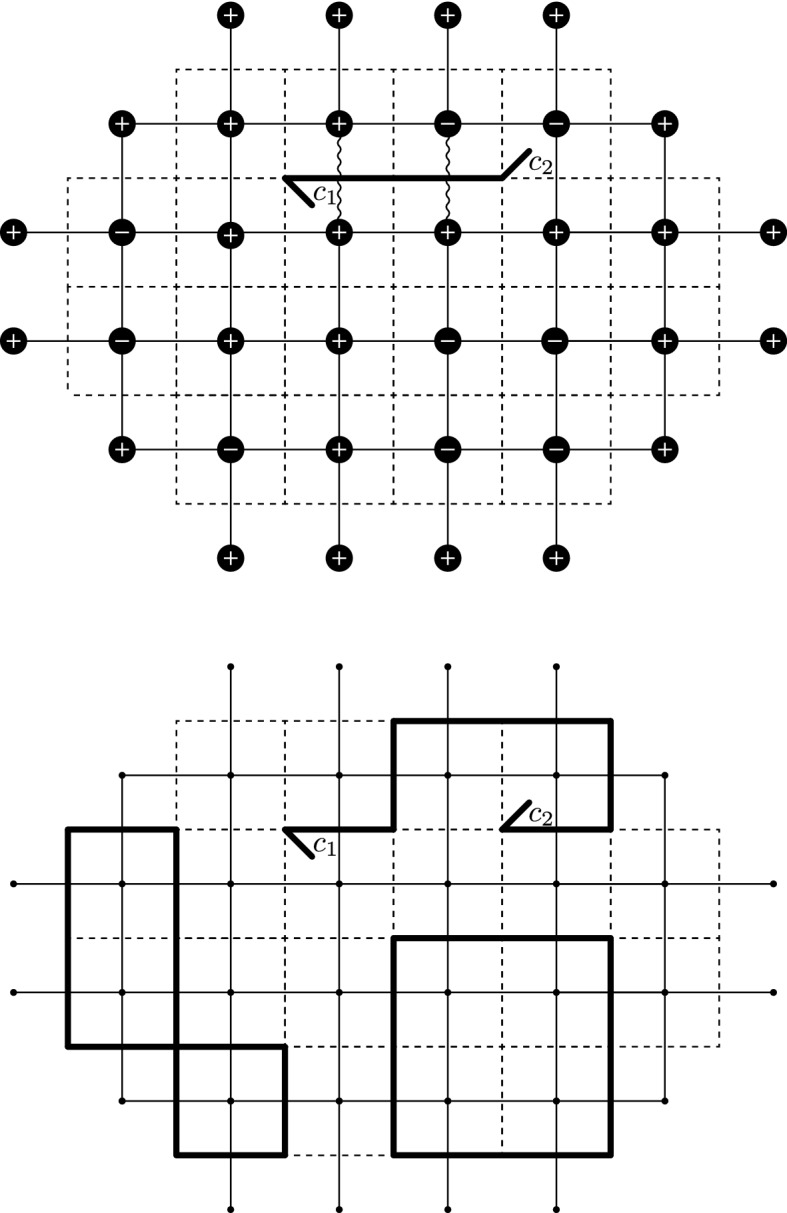
Spin configuration with disorder (top picture) and the associated ${\gamma }$ configuration (bottom picture)

Pick a disorder line ${\varrho }$ between the dual vertices $p_1$ and $p_2$ adjacent to corners $c_1$ and $c_2$, respectively. Represent any Ising configuration ${\sigma }\in \left\{ \pm 1 \right\} ^{{{\Omega }}_{{\delta }}}$ by the following collection ${\gamma }$ of dual-edges: for any edge $e=xy$ of ${{\Omega }}_{{\delta }}$, its dual edge $e^{*}$ belongs to ${\gamma }$ either if $e^{*}\notin {\varrho }$ and ${\sigma }_{x}\ne {\sigma }_{y}$ or if $e^{*}\in {\varrho }$ and ${\sigma }_{x}={\sigma }_{y}$ (see Fig. 13).^9^ It is elementary to check that the effective Boltzmann weight $e^{-{{\beta }}{\mathcal {H}}[{\sigma }] - 2 {{\beta }}{{\mathcal {E}}}_{{\varrho }} [{\sigma }]}$ of a spin configuration ${\sigma }$ in the presence of the disorder line ${\varrho }$ is proportional to $e^{-2{{\beta }}\left| {\gamma }\right| }$ (with the same proportionality factor as in Remark 6.2). Letting $x_{1},x_{2} \in {{\Omega }}_{{\delta }}$ be the vertices adjacent to $c_{1},c_{2}$, it is again elementary to check that the value of ${\sigma }_{x_{1}}{\sigma }_{x_{2}}$ is determined by ${\gamma }$ and equals . The proof follows readily. $\square$

The above lemma generalizes to multiple spin insertions, if this time we keep track of the defect line.

#### Lemma 6.4

Consider the Ising model on a discrete domain ${{\Omega }}_{{\delta }}$ with $+$ boundary conditions. Let $V\subset {{\Omega }}_{{\delta }}$. For any corners $c_{1},c_{2}$, we have6.1

#### Proof

The proof is very similar to that of Lemma 6.3: we define the same contour ${\gamma }$ to describe the configuration. The only additional thing to check is that, denoting as before $x_{1},x_{2}$ the vertices adjacent to $c_{1},c_{2}$, we have . This can be done by induction on the number of vertices in *V*. $\square$

### Discrete holomorphicity

In this subsection, we give the proof of Proposition 3.22, which is central to the lattice Virasoro construction.

The proposition concerns the discrete holomorphicity and singularities of a certain function *H*. We first prove intermediate results about two related functions, defined as follows. Consider the critical Ising model on a domain ${{\Omega }}_{{\delta }}$ with arbitrary boundary conditions. The following will be fixed throughout below:

- Let $W \subset V \subset {{\Omega }}_{{\delta }}$ be two subsets of vertices, and assume that *V* is connected.
- Let $c_1 \in {\left[ {{\Omega }}_{{\delta }}^{{\mathfrak {c}}}, V \right] }$ be a corner on the double cover of ${{\Omega }}_{{\delta }}$ ramified around *V*, whose base point $\underline{c}_1 \in {{\Omega }}_{{\delta }}^{{\mathfrak {c}}}$ on the original lattice is not adjacent to *V*. Denote the adjacent vertex and dual vertex to the base point corner $\underline{c}_1$ by $v_1 \in {{\Omega }}_{{\delta }}$ and $p_1 \in {{\Omega }}_{{\delta }}^*$. We will also occasionally denote by $c_1^* \in {\left[ {{\Omega }}_{{\delta }}^{{\mathfrak {c}}}, V \right] }$ the corner with the same base point $\underline{c}_1$ but on the opposite sheet of the double cover.

We define a function

$$\begin{aligned} H^{\mathfrak {c}}:{\left[ {{\Omega }}_{{\delta }}^{{\mathfrak {c}}}, V \right] } \setminus \left\{ c_1, c_1^* \right\} \rightarrow {\mathbb {C}}\end{aligned}$$

on the corners of the double cover graph by the following formula: for $c_2 \in {\left[ {{\Omega }}_{{\delta }}^{{\mathfrak {c}}}, V \right] } \setminus \left\{ c_1, c_1^* \right\}$ set

6.2 $$\begin{aligned} H^{\mathfrak {c}}( c_{2} ) = H^{\mathfrak {c}}( c_{1} ; c_{2} )&:= {\mathbb {E}}\! \left[ { \left( \prod _{u \in W} {\sigma }_{u} \right) {\psi }_{c_{1}} {\psi }_{c_{2}}} \right] . \end{aligned}$$

The fact that this is well defined on the double cover is a consequence of Proposition 3.17. In concrete terms, if $v_2 \in {{\Omega }}_{{\delta }}$ and $p_2 \in {{\Omega }}_{{\delta }}^*$ are the vertex and dual vertex adjacent to the base point  $\underline{c}_2$, this definition can be unraveled to the formwhere the disorder line ${\varrho }$ is chosen so that its lift to the double cover connects $c_1$ to $c_2$ (when augmented by the two corner ends), and where as in Sect. 3.3.2 we denote $\varvec{\nu }(c_1) = \frac{v_1-p_1}{|v_1-p_1|}$.

We define a related function

$$\begin{aligned} H^{\mathfrak {m}}:{\left[ {{\Omega }}_{{\delta }}^{{\mathfrak {m}}}, V \right] } \rightarrow {\mathbb {C}}\end{aligned}$$

on the medial lattice by the following formula: for $z \in {\left[ {{\Omega }}_{{\delta }}^{{\mathfrak {m}}}, V \right] }$ we set

6.3 $$\begin{aligned} H^{\mathfrak {m}}(z) = H^{\mathfrak {m}}( c_{1} ; z ) := \frac{1}{2} \sum _{x\in \left\{ \pm 1 \pm \mathbb {i} \right\} } {\mathbb {E}}\! \left[ { \left( \prod _{u \in W}{\sigma }_{u}\right) {\psi }_{c_{1}} {\psi }_{z_{x}} } \right] , \end{aligned}$$

where $z_{x} := z + \frac{{\delta }}{4} x \in {\left[ {{\Omega }}_{{\delta }}^{{\mathfrak {c}}}, V \right] }$ denotes a corner adjacent to the medial vertex *z*, in the direction specified by *x*, and in the case $z_{x}=c_{1}$ (resp. $z_{x}=c_{1}^*$), we interpret ${\psi }_{c_{1}} {\psi }_{z_{x}} = \frac{\overline{z}-\overline{w}}{\left| z-w\right| }$ (resp. ${\psi }_{c_{1}} {\psi }_{z_{x}} = -\frac{\overline{z}-\overline{w}}{\left| z-w\right| }$) where *w* is the other medial vertex besides *z*, which is adjacent to $c_{1}$ (resp. $c_{1}^*$).

#### Lemma 6.5

Let *z* be a medial vertex of the double cover ${\left[ {{\Omega }}_{{\delta }}^{{\mathfrak {m}}}, V \right] }$. If *z* is not adjacent to the corners $c_{1}, c_{1}^*$, i.e., $z_{x} \ne c_{1}, c_{1}^*$ for each $x\in \left\{ \pm 1\pm \mathbb {i} \right\}$, then we have

6.4 $$\begin{aligned} H^{\mathfrak {c}}\left( z_{x}\right) + H^{\mathfrak {c}}\left( z_{-x}\right) = H^{\mathfrak {c}}\left( z_{\mathbb {i}x}\right) + H^{\mathfrak {c}}\left( z_{-\mathbb {i}x}\right) . \end{aligned}$$

If $z_{x}=c_{1}$ for some $x\in \left\{ \pm 1\pm \mathbb {i} \right\}$, and $w \in {\left[ {{\Omega }}_{{\delta }}^{{\mathfrak {m}}}, V \right] }$ is the other medial edge adjacent to $c_{1}$, then we have

6.5 $$\begin{aligned} \frac{\overline{z}-\overline{w}}{\left| z-w\right| } \; {\mathbb {E}}\! \left[ { \prod _{x\in V} {\sigma }_{x} } \right] + H^{\mathfrak {c}}\left( z_{-x}\right) = H^{\mathfrak {c}}\left( z_{\mathbb {i}x}\right) + H^{\mathfrak {c}}\left( z_{-\mathbb {i}x}\right) . \end{aligned}$$

in

#### Proof

Let us first assume that we have $+$ boundary conditions. Let *e* be the dual edge whose midpoint is *z* and for each $x\in \left\{ \pm 1 \pm \mathbb {i} \right\}$, let $e_{x}$ denote the corner end between $z_x$ and a dual vertex adjacent to *z*.

Let us first assume that $z_{x} \ne c_{1}, c_{1}^*$ for all *x*. Let us write the values of $H^{\mathfrak {c}}\left( z_{x}\right)$ in terms of the low-temperature expansion (Lemma 6.4), choosing defect lines ${\lambda }_{x}:c_{1}\leftrightarrow z_{x}$ which differ only locally, and let ${{\mathscr {C}}}_{x} := {{\mathscr {C}}}_{c_{1},z_{x}}$ denote the relevant sets of $(c_1 , z_x)$-subgraphs. Let $W_{x}$ denote the weight , where $\alpha = e^{-2{{\beta }}_{\mathrm {cr.}}}=\sqrt{2}-1$.

**Fig. 14:**
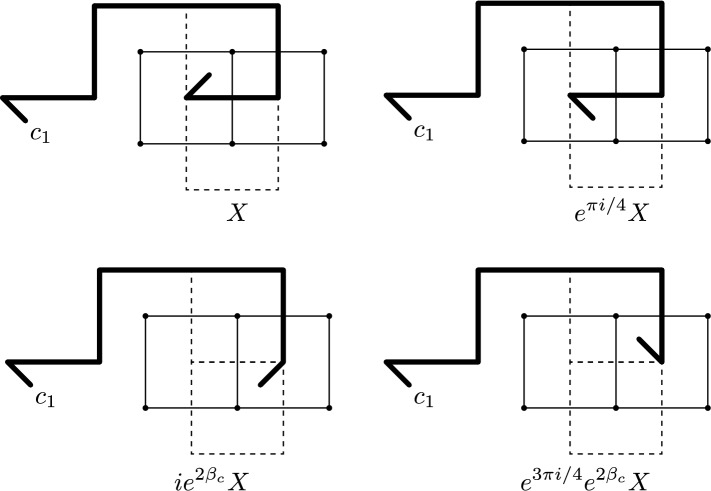
Bijective correspondence between the contour sets corresponding to the corners around a medial vertex, and relative weights of the configuration

The $(c_1 , z_x)$-subgraphs ${\gamma }_{x}\in {{\mathscr {C}}}_{x}$ for different *x* can be put in bijective correspondence (see Fig. 14): the map ${{\mathscr {C}}}_{x} \rightarrow {{\mathscr {C}}}_{-x}$ can be defined by taking the symmetric difference ${\gamma }_{x}\mapsto {\gamma }_{x}\oplus e_{x}\oplus e_{-x}\oplus e$, for instance, and if ${{\mathscr {C}}}_{x}$ and ${{\mathscr {C}}}_{\mathbb {i}x}$, the bijection share the same dual vertex, ${\gamma }_{x}\mapsto {\gamma }_{x}\oplus e_{x}\oplus e_{\mathbb {i}x}$. It is elementary to check that for any $\left( {\gamma }_{x},{\gamma }_{\mathbb {i}x},{\gamma }_{-x},{\gamma }_{-\mathbb {i}x}\right)$ thus put in correspondence, we have

$$\begin{aligned} W_{x}+W_{-x}=W_{\mathbb {i}x}+W_{-\mathbb {i}x}. \end{aligned}$$

Summing over all contours, we obtain (6.4), as desired.

**Fig. 15:**
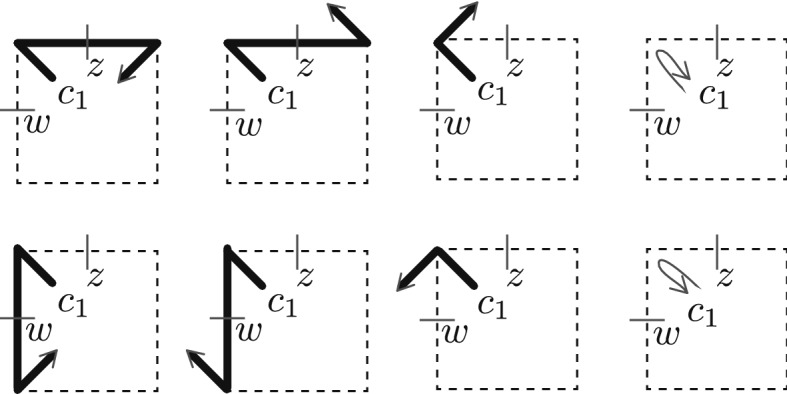
Source of the discrete singularity when *z* is near the point $c_{1}$: performing the bijection as before, we obtain two ‘conflicting’ values of ${\mathbf {W}}\left( {\gamma }\right) =-\pi$ (top) and ${\mathbf {W}}\left( {\gamma }\right) =\pi$ (bottom) on the last column

If $z_{x}=c_{1}$ for some $x \in \left\{ \pm 1 \pm \mathbb {i} \right\}$, then $H^{\mathfrak {c}}\left( z_{x}\right)$ is not defined. However, if we would set $H^{\mathfrak {c}}\left( z_{x}\right)$ equal to $\frac{\overline{z}-\overline{w}}{|z-w|} {\mathbb {E}}\! \left[ { \prod _{x\in V} {\sigma }_{x} } \right]$ (this choice is one of the two “conflicting values” addressed below the proof), and represent the latter in terms of even subgraphs in ${{\mathscr {C}}}_{x} = {{\mathscr {C}}}$ (there is no path in such even subgraphs, just loops), the bijective correspondence works in exactly the same way as above (see Fig. 15). This hence yields (6.5).

For more general boundary conditions, simply notice that they can be implemented by (a linear combination of) spin insertions on the boundary, and hence can be included in *V*. $\square$

In the proof we temporarily used a certain value of the function $H^{\mathfrak {c}}$ also at the special corner $c_1$. However, the value depended on which of the two medial edges *z* adjacent to $c_1$ was under consideration—the other adjacent medial edge would have required us to use the opposite value. These “conflicting values” are the source of the singularity of our discrete holomorphic function.

The following lemma, combined with the previous one (Lemma 6.5), allows one to say that (at criticality) the values of the function $H^{\mathfrak {c}}$ that appear in Equation (6.4) are actually the projection of an extension $H^{\mathfrak {m}}$ of $H^{\mathfrak {c}}$ to the medial vertices (as will Lemma 6.7 below show): indeed the values given by opposite corners around a medial vertex live on orthogonal lines of the complex plane (see [Smi10a] for a reference about such considerations).

As usual, we set $\lambda := e^{\pi \mathbb {i}/4}$.

#### Lemma 6.6

**Fig. 16:**
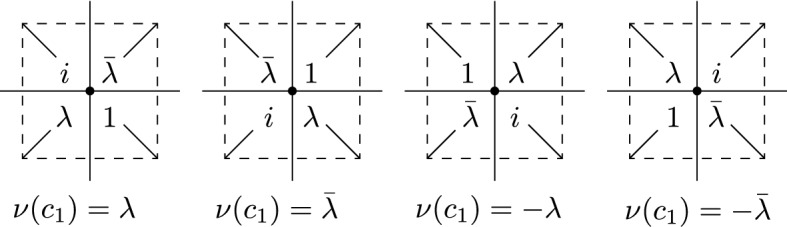
The directions of the lines $\ell \left( c_{1},c_{2}\right)$ in ${\mathbb {C}}$ on which $H^{\mathfrak {c}}\left( c_{2}\right)$ lives, for the four corners around a primal vertex

For any $c_2 \in {\left[ {{\Omega }}_{{\delta }}^{{\mathfrak {c}}}, V \right] } \setminus \left\{ c_1, c_1^* \right\}$, we have that $H^{\mathfrak {c}}\left( c_{2}\right) \in \ell \left( c_{1},c_{2}\right)$, where $\ell \left( c_{1},c_{2}\right) := \mathbb {i}\sqrt{{\bar{{\nu }}}(c_{1}) {\bar{{\nu }}}(c_{2})} {\mathbb {R}}$ (see Fig. 16).

#### Proof

This is elementary from the definition of $H^{\mathfrak {c}}$ and of the corner fermion pair $({\psi }_{c_1}{\psi }_{c_2})$.

$$\square $$

We are now in position to state the following discrete holomorphicity lemma for $H^{\mathfrak {m}}$. Set $\lambda := e^{\pi \mathbb {i}/4}$ and $\eta := e^{\pi \mathbb {i}/8}$ as usual.

#### Lemma 6.7

If $\zeta \in {\left[ {{\Omega }}_{{\delta }}^{\diamond }, V \right] }$ is away from *V*, $\partial {{\Omega }}_{{\delta }}$, and $c_{1}, c_{1}^{*}$ then we have ${{{\bar{\partial }}}}_{{\delta }}H^{\mathfrak {m}}(\zeta ) =0$. If $\zeta$ is the vertex or the dual vertex next to $c_{1}$, then we have ${{{\bar{\partial }}}}_{{\delta }}H^{\mathfrak {m}}(\zeta ) = \sqrt{2} \, {\mathbb {E}}\! \left[ { \prod _{x\in W} {\sigma }_{x} } \right]$.

#### Proof

Consider a medial vertex *z* and the corners $z_x$ adjacent to it, for $x \in \left\{ \pm 1 \pm \mathbb {i} \right\}$. If *z* is not adjacent to $c_1 , c_1^*$, then by Lemmas 6.5 and 6.6, we have that the values of $H^{\mathfrak {c}}(z_{x})$ at these corners are given by the projections of the value of $H^{\mathfrak {m}}(z)$ on certain lines. The line $\ell (c_1, z_x) = \xi _{c_1 , z_x} \, {\mathbb {R}}$ is specified by a complex number $\xi _{c_1 , z_x}$ of unit modulus as in Fig. 16, and the projection relation explicitly reads

6.6 $$\begin{aligned} H^{\mathfrak {c}}(z_{x}) = \mathrm {pr}_{\ell (c_1, z_x)} \big [ H^{\mathfrak {m}}(z) \big ] = \frac{1}{2} \Big ( H^{\mathfrak {m}}(z) + \xi _{c_1 , z_x}^2 \, \overline{H^{\mathfrak {m}}(z)} \Big ) . \end{aligned}$$

When $z_{x} = c_{1}$, then the corresponding projection relation holds with one of the two opposite “conflicting values” for ${\psi }_{c_{1}} {\psi }_{c_1}$.

Let us consider the case when ${\nu }(c_{1}) = \lambda$ in detail; the other cases are symmetric. Set $\Xi := {\mathbb {E}}\! \left[ { \prod _{x\in W} {\sigma }_{x} } \right]$. We have the following four cases:

1. If $\zeta$ is a primal vertex away from $c_{1}, c_{1}^{*}$, denoting by *E*, *N*, *W*, *S* the values of $H^{\mathfrak {m}}$ at the four medial vertices next to $\zeta$ in the directions $1,\mathbb {i},-1,-\mathbb {i}$, the projection relations (6.6) read:
  $$\begin{aligned} N-\mathbb {i}{\bar{N}}&=E-\mathbb {i}{\bar{E}}\\ S+{\bar{S}}&=E+{\bar{E}}\\ S+\mathbb {i}{\bar{S}}&=W+\mathbb {i}{\bar{W}}\\ N-{\bar{N}}&=W-{\bar{W}}. \end{aligned}$$
  By taking an appropriate linear combination of the four equations to eliminate the complex conjugate values, one gets $E-W+\mathbb {i}\left( N-S\right) =0$, which shows ${{{\bar{\partial }}}}_{{\delta }}H^{\mathfrak {m}}(\zeta ) = 0$ as desired.
2. If $\zeta = v_1$ is the primal vertex adjacent to $c_{1}$, plugging in the two “conflicting” values given by Equation (6.5) of Lemma 6.5 (and reading them as projection relations again), and using the same notation as in the previous item, one gets
  $$\begin{aligned} S + \mathbb {i}{\bar{S}}&= 2 \lambda \, \Xi \\ - 2 \lambda \, \Xi&= W + \mathbb {i}{\bar{W}}\\ S + {\bar{S}}&= E + {\bar{E}}\\ N - \mathbb {i}{\bar{N}}&= E - \mathbb {i}{\bar{E}}\\ N - {\bar{N}}&= W - {\bar{W}}. \end{aligned}$$
  By again taking an appropriate linear combination of these five equations, one gets $E - W + \mathbb {i}\left( N-S\right) = 2 \sqrt{2} \, \Xi$ and hence ${{{\bar{\partial }}}}_{{\delta }}H^{\mathfrak {m}}(v_1) = \sqrt{2} \, \Xi$, as desired.
3. If $\zeta$ is a dual vertex away from $c_{1} ,c_{1}^{*}$, and if we denote by ${\mathcal {E}},{\mathcal {N}},{\mathcal {W}},{\mathcal {S}}$ the values of $H^{\mathfrak {m}}$ at the medial vertices around $\zeta$ directions $1,\mathbb {i},-1,-\mathbb {i}$, the projection relations (6.6) read
  $$\begin{aligned} {\mathcal {N}}+\bar{{\mathcal {N}}}&={\mathcal {W}}+\bar{{\mathcal {W}}}\\ {\mathcal {S}}-\mathbb {i}\bar{{\mathcal {S}}}&={\mathcal {W}}-\mathbb {i}\bar{{\mathcal {W}}}\\ {\mathcal {S}}-\bar{{\mathcal {S}}}&={\mathcal {E}}-\bar{{\mathcal {E}}}\\ {\mathcal {N}}+\mathbb {i}\bar{{\mathcal {N}}}&={\mathcal {E}}+\mathbb {i}\bar{{\mathcal {E}}}. \end{aligned}$$
  As in the first item, an appropriate linear combination of these yields ${{{\bar{\partial }}}}_{{\delta }}H^{\mathfrak {m}}(\zeta ) = 0$.
4. Finally, if $\zeta =p_1$ is the dual vertex adjacent to $c_{1}$, using the same notation as in the previous item, one gets
  $$\begin{aligned} 2\lambda \, \Xi&={\mathcal {E}} + \mathbb {i}\bar{{\mathcal {E}}}\\ {\mathcal {N}} + \mathbb {i}\bar{{\mathcal {N}}}&= - 2 \lambda \, \Xi \\ {\mathcal {N}} + \bar{{\mathcal {N}}}&= {\mathcal {W}} + \bar{{\mathcal {W}}}\\ {\mathcal {S}} - \mathbb {i}\bar{{\mathcal {S}}}&= {\mathcal {W}} - \mathbb {i}\bar{{\mathcal {W}}}\\ {\mathcal {S}} - \bar{{\mathcal {S}}}&= {\mathcal {E}} - \bar{{\mathcal {E}}}, \end{aligned}$$
  and one concludes ${{{\bar{\partial }}}}_{{\delta }}H^{\mathfrak {m}}(p_1) = \sqrt{2} \, \Xi$ as in the second item.

$$\square $$

The function

$$\begin{aligned} H :{\left[ {{\Omega }}_{{\delta }}^{{\mathfrak {m}}}, V \right] } \rightarrow {\mathbb {C}}\end{aligned}$$

considered in Proposition 3.22 is straightforwardly reconstructed from $H^{\mathfrak {m}}$. For its definition, $c_1$ is no longer fixed, but we average over the four corners $c_1$ next to a fixed medial vertex $w \in {\left[ {{\mathbb {C}}_{{\delta }}^{{\mathfrak {m}}}}, V \right] }$. The function *H* is defined as

$$\begin{aligned} H(z) = H(w; z)&:= {\mathbb {E}}\! \left[ { \left( \prod _{u\in W}{\sigma }_{u}\right) {\psi }(w) {\psi }(z) } \right] \\&= \frac{\pi }{8 \sqrt{2}} \sum _{x,y \in \left\{ \pm 1 \pm \mathbb {i} \right\} } {\mathbb {E}}\! \left[ { \left( \prod _{u\in W}{\sigma }_{u}\right) {\psi }_{w_y} {\psi }_{z_x} } \right] \end{aligned}$$

Up to a multiplicative constant $\frac{\pi }{4\sqrt{2}}$, this is just the sum of $H^{\mathfrak {m}}(z)=H^{\mathfrak {m}}(c_1;z)$ over the four corners $c_1 = w_y$ adjacent to *w*. We now recall and prove Proposition 3.22 concerning the function *H*.

#### Proposition

(Proposition 3.22) For $\zeta \in {\left[ {{\Omega }}_{{\delta }}^{\diamond }, V \right] }$ away from $\partial {{\Omega }}_{{\delta }}$, *V*, we have ${{{\bar{\partial }}}}_{{\delta }}H(\zeta ) =0$ if $\zeta \not \sim w, w^{*}$ and we have

$$\begin{aligned} {{{\bar{\partial }}}}_{{\delta }}H(\zeta ) = \frac{\pi }{2} \, {\mathbb {E}}\! \left[ { \prod _{x\in W} {\sigma }_{x} } \right] \end{aligned}$$

if $$\zeta \sim w$$.

#### Proof of Proposition 3.22

This property of *H* follows directly from Lemma 6.7 about $H^{\mathfrak {m}}$. $\square$

## Footnotes

1 In the functional integral picture, *T* is defined via the variation of the action with respect to a change of metric; informally the insertion of *T* hence represents a change of measure (à la Radon-Nikodym derivatives) corresponding to an infinitesimal change of metric.

2 Again, this should be interpreted within correlations only.

3 Since a field is merely an object one can take correlations of, this indeed defines a field. Also, since the correlations of *T*(*z*) are holomorphic, one can just take $\epsilon$ small enough. (But how small $\epsilon$ needs to be depends on the locations of the other inserted fields in the correlation.)

4 The idea is that various limits will be of interest, which might involve renormalizing by certain powers of ${\delta }_{k}$ (depending on ${{\mathcal {O}}}^{{\delta }}$ in particular) as $k \rightarrow \infty$.

5 This is also the point of view which was chosen as the primary point of view in an earlier version of the present paper.

6 Note that compared to our paper, the lattice is rotated by 45 degrees in [CHI15], that the the lattice square side length is denoted there by $\sqrt{2}\,\delta$ that the primal graph there corresponds to the dual graph.

7 The square lattice used in [GHP19] is $(1+\mathbb {i}) {\delta }\, {\mathbb {Z}}^2$, and correspondingly the side-length of the squares is $\sqrt{2}{\delta }$ instead of ${\delta }$, and the orientation differs by 45 degrees.

8 Informally, an even subgraph ${\vartheta }$ of ${{\Omega }}_{{\delta }}^*$ forms a collection of loops on the dual graph. Even subgraphs are also called *contours* in the literature, but we have chosen to avoid this term because it is already used in the context of discrete integrals.

9 In other words, ${\gamma }$ represents the ‘frustrated pairs’ of adjacent spins
